# Supplementary material for: Species responses to weather anomalies depend on local adaptation and range position
Source: Commun Biol. 2025 Apr 24;8:660. doi: 10.1038/s42003-025-08032-9 (PMC12022152; doi:10.1038/s42003-025-08032-9)
Supplement: Supplementary file 1 — Supplementary Material [file 42003_2025_8032_MOESM1_ESM.pdf]

Species responses to weather anomalies depend on local adaptation and range position

Yolanda Melero, Luke C. Evans, Mikko Kuussaari, Reto Schmucki, Constantí Stefanescu, David B. Roy, Tom H. Oliver

Supplementary Note: Glossary of terms

Distributional range position: The position of a population within a species distributional range, the latter defined as the geographic area over which a species is found. The population position in the geographic range can be defined relative to several axis e.g. north south, or through geometric measures e.g. population distance from the centroid of the distribution.

Bioclimatic Niche: The set of climatic conditions that determine the environmental space in which a species can maintain stable populations. In the manuscript, the bioclimatic niche is a single axis calculated by mapping the range of climatic values of the variable most affecting the species (temperature, precipitation or aridity) where the species is detected, and standardizing these values between -1 (minimum) and 1 (maximum) to account for variations across different sites and species, i.e. for a given species a score of 1 would be the population experiencing the highest average temperatures and -1 the population experiencing the lowest. For ectotherms, the bioclimatic niche aligns closely with their distributional range due to the significant influence of temperature on their physiological processes[1–3], although it can be different due to factors such as altitude.

Performance Curve: Describes the relationship between a species population growth rate and the surrounding environmental conditions. This curve illustrates how well a species performs (in terms of growth or survival) under varying levels of specific environmental factors. For ectotherms, temperature is often the key environmental factor considered (e.g., thermal performance curves)[4]. However, other factors, such as moisture or resource availability, may also shape these curves, influencing a species' capacity to thrive under different conditions.

Degree of local adaptation: There are several aspects related to local adaption (e.g. genetic, morphological, behavioural variation), here we focus on physiological (thermal performance) adaptation through a quantitative measurement of a species sensitivity (in terms of interannual population change) to climatic anomalies (deviations from the average conditions) at the local scale. Our score of local adaptation is calculated based on the difference in explanatory power (R^2) of models using local versus global climatic anomalies, indicating the relative influence of local climate on population dynamics. Value range [-1, 1][5]. A higher value suggests greater sensitivity to local conditions, while lower or negative values indicate a stronger response to broader, globally averaged climatic trends. This metric helps assess how well species are adapted to local environmental

conditions, i.e. to the local climate, compared to the conditions experienced across their entire bioclimatic niche space.

Locally Adapted Species: Species whose population dynamics are more strongly influenced by climatic anomalies at a local scale (e.g., deviations from average conditions at specific sites). These species exhibit a higher sensitivity to changes in local climate conditions, with their interannual population changes being best explained by variations in local climatic factors (temperature, precipitation or aridity). This adaptation suggests that the species performance is optimized at the typical conditions of their local area, but falls off away from the typical conditions. This is observed via a higher degree of local adaptation measured by the relative explanatory power (R^2) of the local climate models. Locally adapted species show degree of local adaptation > 0, with a maximum at 1.

Globally Adapted Species: Species whose population dynamics are more influenced by broader-scale climatic anomalies, such as deviations from average conditions calculated across their entire bioclimatic niche space. These species show higher sensitivity to global climate patterns rather than site-specific conditions, meaning that their population changes align more closely with a broader response centred on the global climate averages. Their adaptation allows them to maintain stable populations across varying local conditions, reflecting a lower degree of local adaptation and a greater reliance on global climatic factors for survival and reproduction. Globally adapted species show degree of local adaptation < 0, with maximum value at -1.

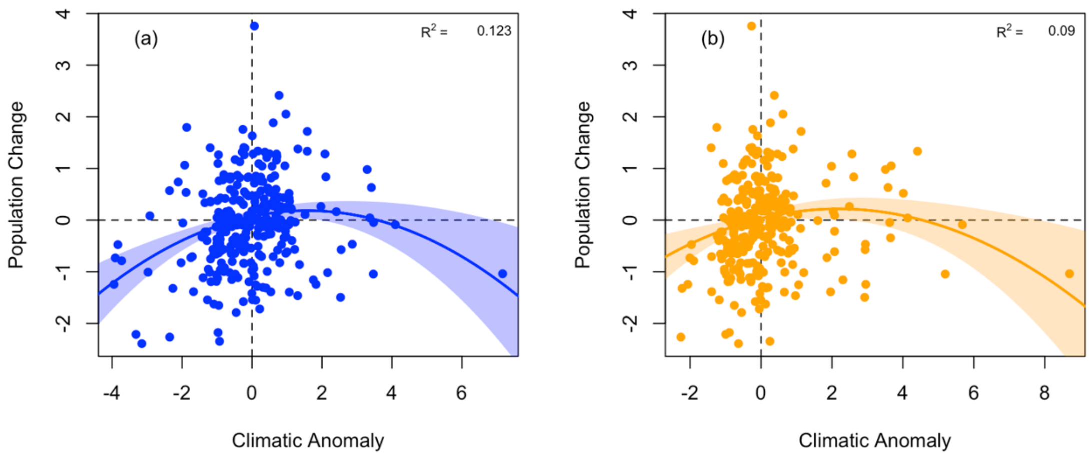

Supplementary Figure 1. Population change in relation to anomalies for (a) local and (b) global responses respectively for *Argynnis adippe*, a species best adapted to local climatic anomalies in precipitation during the pre-flight period of the previous year (*t-1*) of their adult stage (n = 1346). Local climatic anomalies refers to the deviations from the average conditions at the population site. Global climatic anomalies refers to the deviations from the average conditions across the species range. Colours indicate spatial scale (blue, local; orange, global), circles indicate raw data. R^2 values are provided. The degree of local adaptation dla = 0.033, with positive values indicating local adaptation. Adapted from Melero et al. 2022[5].

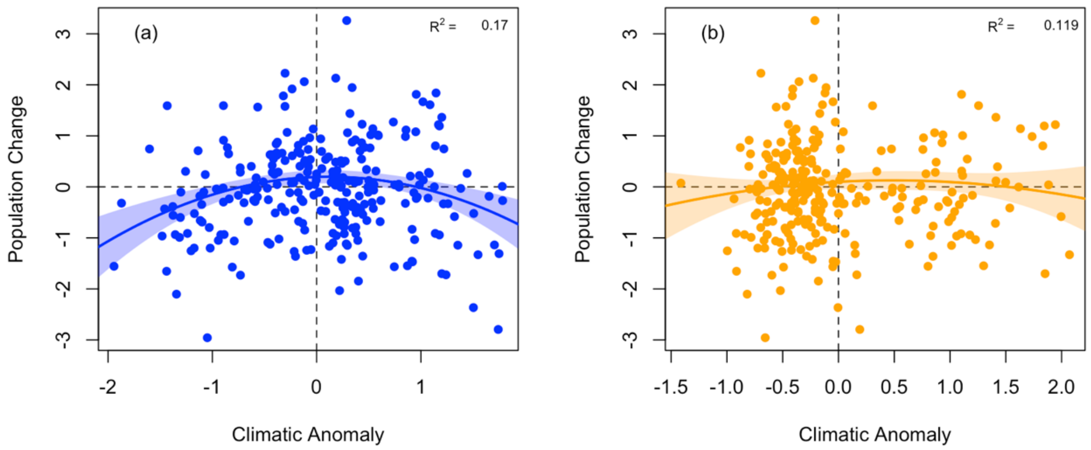

Supplementary Figure 2. Population change in relation to anomalies for (a) local and (b) global responses respectively for *Aririca agestis*, a species best adapted to local climatic anomalies in temperature during the flight period of the previous year (*t-1*) of their adult stage (n = 5648). Local climatic anomalies refers to the deviations from the average conditions at the population site. Global climatic anomalies refers to the deviations from the average conditions across the species range. Colours indicate spatial scale (blue, local; orange, global), circles indicate raw data. R^2 values are provided. The degree of local adaptation dla = 0.051, with positive values indicating local adaptation. Adapted from Melero et al. 2022[1].

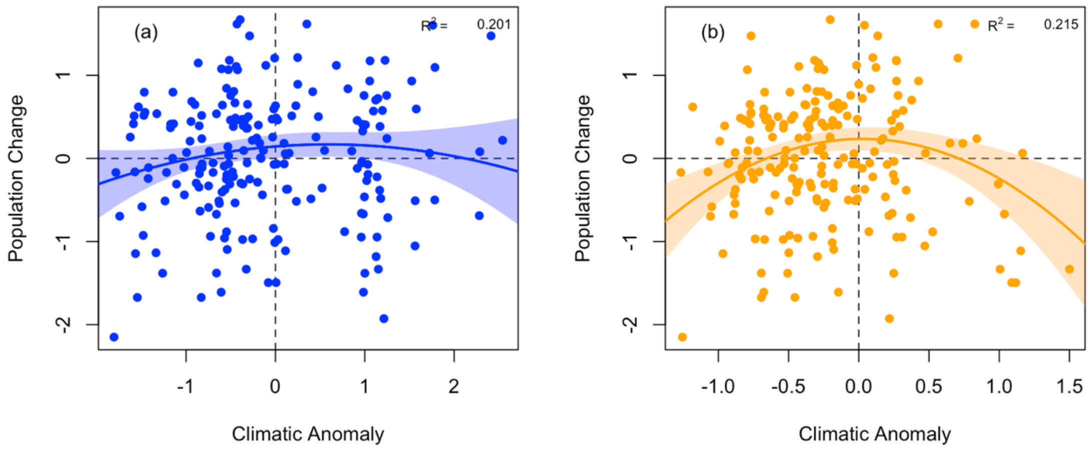

Supplementary Figure 3. Population change in relation to anomalies for (a) local and (b) global responses respectively for *Aricia artaxerxes*, a species best adapted to global climatic anomalies in temperature during the pre-flight period of the year (*t*) of their adult stage (n = 1080). Local climatic anomalies refers to the deviations from the average conditions at the population site. Global climatic anomalies refers to the deviations from the average conditions across the species range. Colours indicate spatial scale (blue, local; orange, global), circles indicate raw data. R^2 values are provided. The degree of local adaptation dla = -0.014, with negative values indicating global adaptation. Adapted from Melero et al. 2022[1].

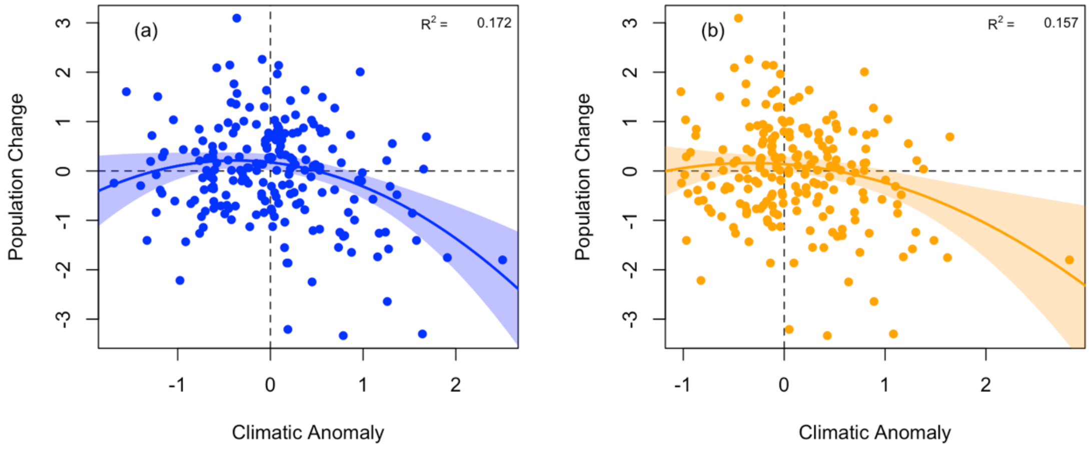

Supplementary Figure 4. Population change in relation to anomalies for (a) local and (b) global responses respectively for *Boloria dia*, a species best adapted to local climatic anomalies in precipitation during the post flight period of the previous year (*t-1*) of their adult stage (n = 334). Local climatic anomalies refers to the deviations from the average conditions at the population site. Global climatic anomalies refers to the deviations from the average conditions across the species range. Colours indicate spatial scale (blue, local; orange, global), circles indicate raw data. R^2 values are provided. The degree of local adaptation dla = 0.015, with positive values indicating local adaptation. Adapted from Melero et al. 2022[1].

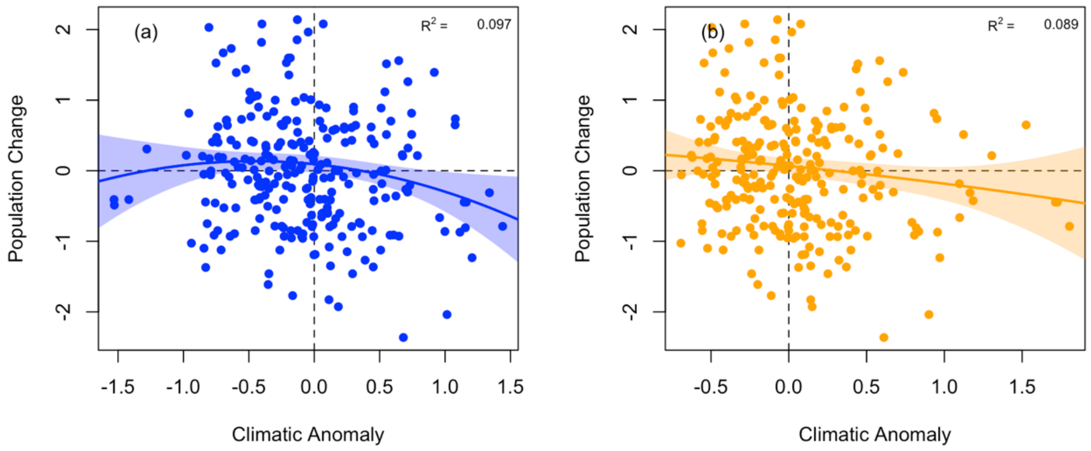

Supplementary Figure 5. Population change in relation to anomalies for (a) local and (b) global responses respectively for *Boloria euphrosyne*, a species best adapted to local climatic anomalies in precipitation during the flight period of the year (*t*) of their adult stage (n = 2026). Local climatic anomalies refers to the deviations from the average conditions at the population site. Global climatic anomalies refers to the deviations from the average conditions across the species range. Colours indicate spatial scale (blue, local; orange, global), circles indicate raw data. R^2 values are provided. The degree of local adaptation dla = 0.008, with positive values indicating local adaptation. Adapted from Melero et al. 2022[1].

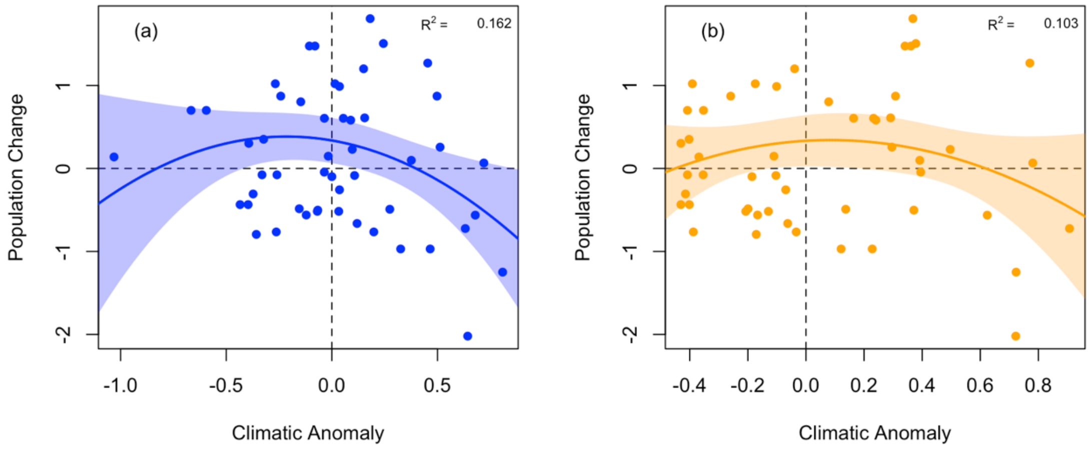

Supplementary Figure 6. Population change in relation to local and global climatic anomalies for (a) local and (b) global responses respectively *Brenthis daphne*, a species best adapted to local climatic anomalies in precipitation during the flight period of the year (*t*) of their adult stage (n = 95). Local climatic anomalies refers to the deviations from the average conditions at the population site. Global climatic anomalies refers to the deviations from the average conditions across the species range. Colours indicate spatial scale (blue, local; orange, global), circles indicate raw data. R^2 values are provided. The degree of local adaptation dla = 0.059, with positive values indicating local adaptation. Adapted from Melero et al. 2022[1].

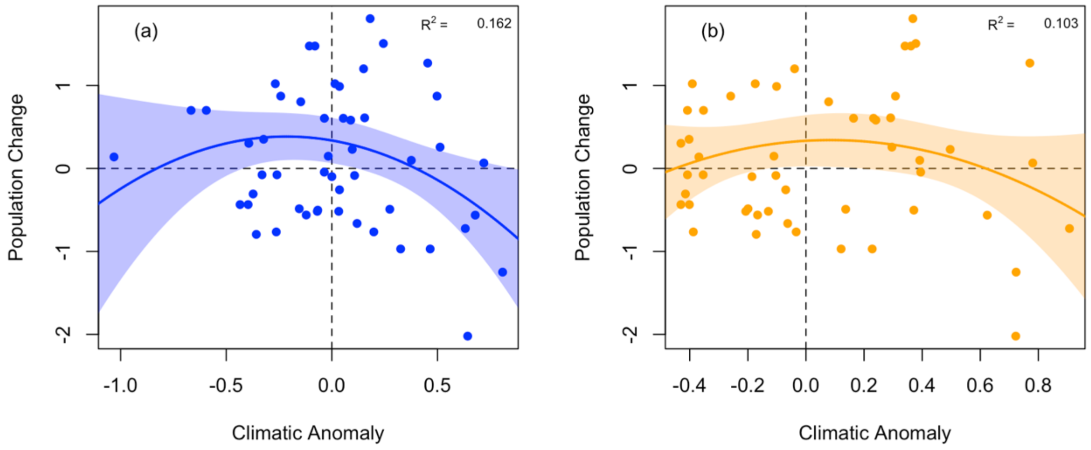

Supplementary Figure 7. Population change in relation to local and global climatic anomalies for (a) local and (b) global responses respectively for *Brenthis ino*, a species best adapted to local climatic anomalies in temperature during the overwintering of the previous year (*t-1*) of their adult stage (n = 734). Local climatic anomalies refers to the deviations from the average conditions at the population site. Global climatic anomalies refers to the deviations from the average conditions across the species range. Colours indicate spatial scale (blue, local; orange, global), circles indicate raw data. R^2 values are provided. The degree of local adaptation dla = 0.062, with positive values indicating local adaptation. Adapted from Melero et al. 2022[1].

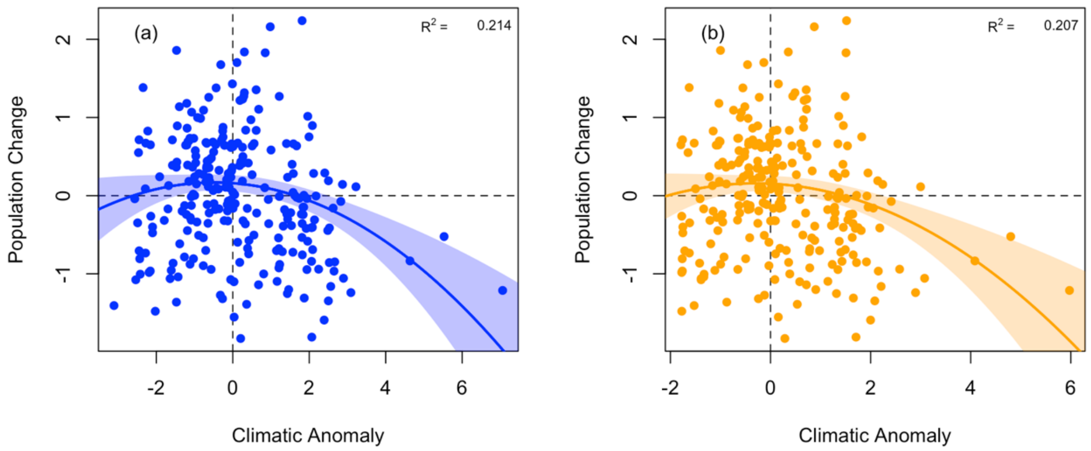

Supplementary Figure 8. Population change in relation to local and global climatic anomalies for (a) local and (b) global responses respectively for *Charaxes jasius*, a species best adapted to local climatic anomalies in precipitation during the pre-flight period of the previous year (*t-1*) of their adult stage (n = 387). Local climatic anomalies refers to the deviations from the average conditions at the population site. Global climatic anomalies refers to the deviations from the average conditions across the species range. Colours indicate spatial scale (blue, local; orange, global), circles indicate raw data. R^2 values are provided. The degree of local adaptation dla = 0.008, with positive values indicating local adaptation. Adapted from Melero et al. 2022[1].

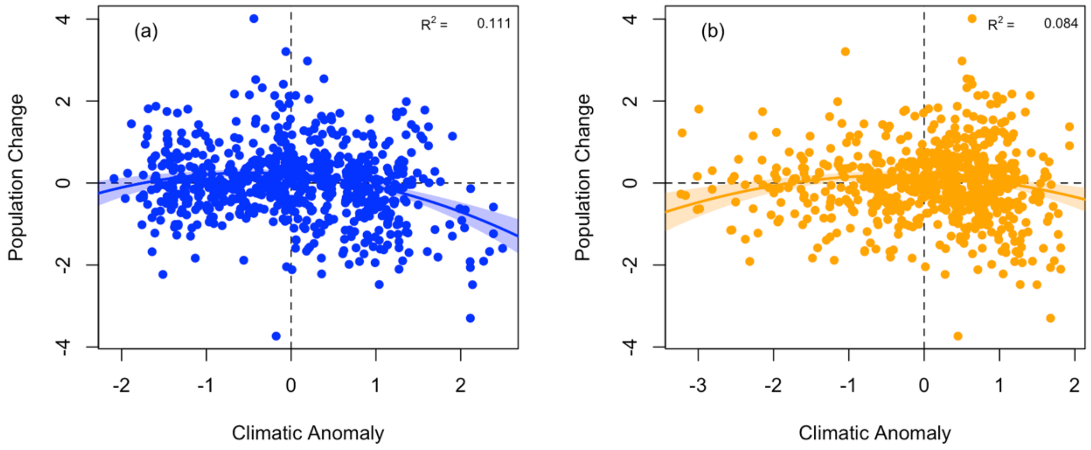

Supplementary Figure 9. Population change in relation to local and global climatic anomalies for (a) local and (b) global responses respectively for *Colias croceus*, a species best adapted to local climatic anomalies in temperature during the post flight period of the previous year (*t-1*) of their adult stage (n = 5822). Local climatic anomalies refers to the deviations from the average conditions at the population site. Global climatic anomalies refers to the deviations from the average conditions across the species range. Colours indicate spatial scale (blue, local; orange, global), circles indicate raw data. R^2 values are provided. The degree of local adaptation dla = 0.026, with positive values indicating local adaptation. Adapted from Melero et al. 2022[1].

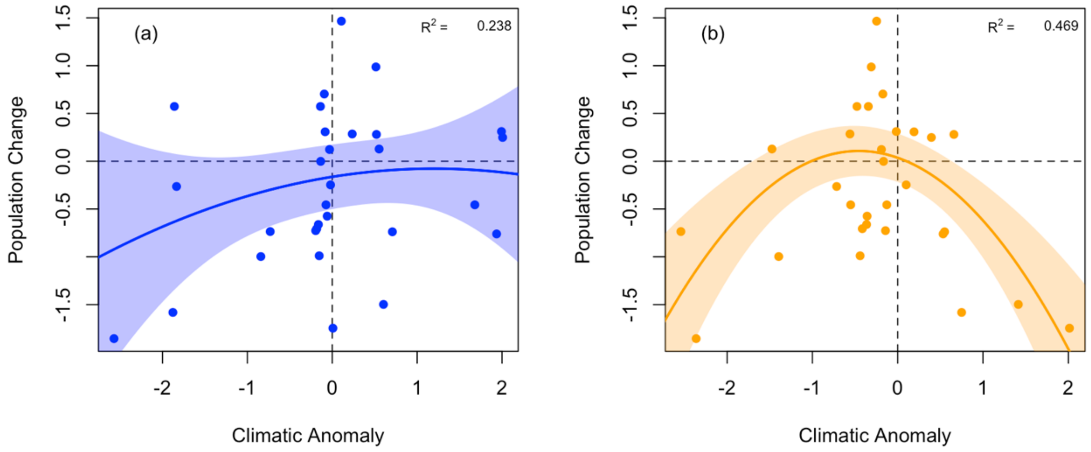

Supplementary Figure 10. Population change in relation to local and global climatic anomalies for (a) local and (b) global responses respectively for *Cupido osiris*, a species best adapted to global climatic anomalies in aridity during the pre-flight period of the year (*t*) of their adult stage (n = 61). Local climatic anomalies refers to the deviations from the average conditions at the population site. Global climatic anomalies refers to the deviations from the average conditions across the species range. Colours indicate spatial scale (blue, local; orange, global), circles indicate raw data. R^2 values are provided. The degree of local adaptation dla = -0.23, with negative values indicating global adaptation. Adapted from Melero et al. 2022[1].

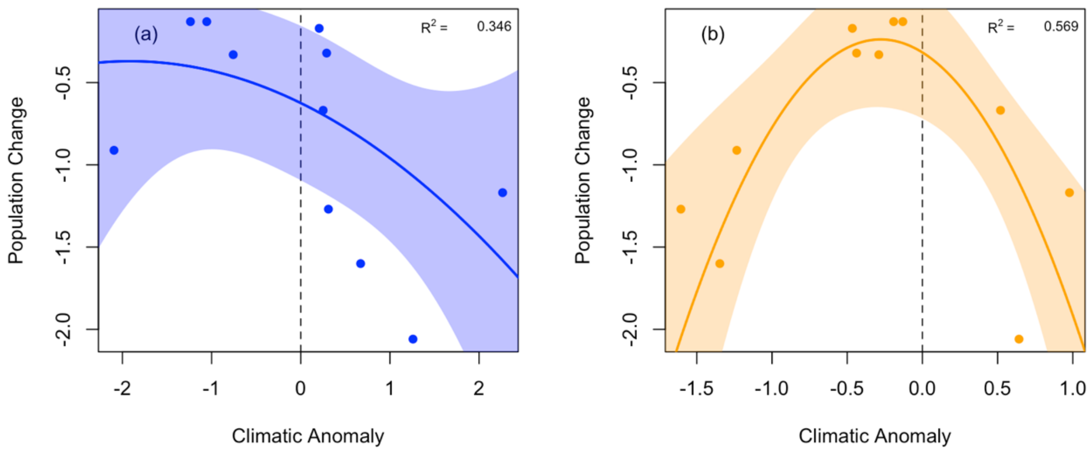

Supplementary Figure 11. Population change in relation to local and global climatic anomalies for (a) local and (b) global responses respectively for *Laeosopis roboris*, a species best adapted to global climatic anomalies in temperature during the flight period of the year (*t*) of their adult stage (n = 33). Local climatic anomalies refers to the deviations from the average conditions at the population site. Global climatic anomalies refers to the deviations from the average conditions across the species range. Colours indicate spatial scale (blue, local; orange, global), circles indicate raw data. R^2 values are provided. The degree of local adaptation dla = -0.22, with negative values indicating global adaptation. Adapted from Melero et al. 2022[1].

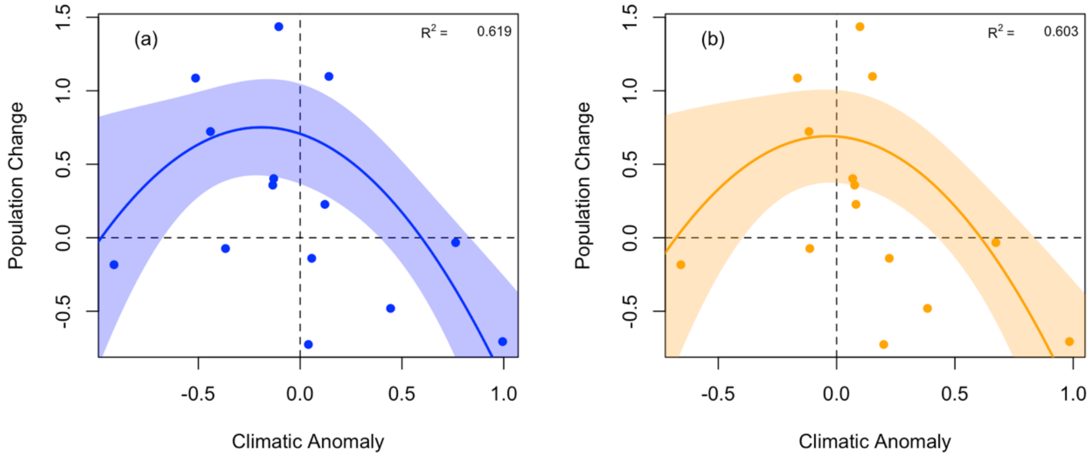

Supplementary Figure 12. Population change in relation to local and global climatic anomalies for (a) local and (b) global responses respectively for *Leptidea juvernica*, a species best adapted to local climatic anomalies in precipitation during the flight period of the previous year (*t-1*) of their adult stage (n = 61). Local climatic anomalies refers to the deviations from the average conditions at the population site. Global climatic anomalies refers to the deviations from the average conditions across the species range. Colours indicate spatial scale (blue, local; orange, global), circles indicate raw data. R^2 values are provided. The degree of local adaptation dla = 0.016, with positive values indicating local adaptation. Adapted from Melero et al. 2022[1].

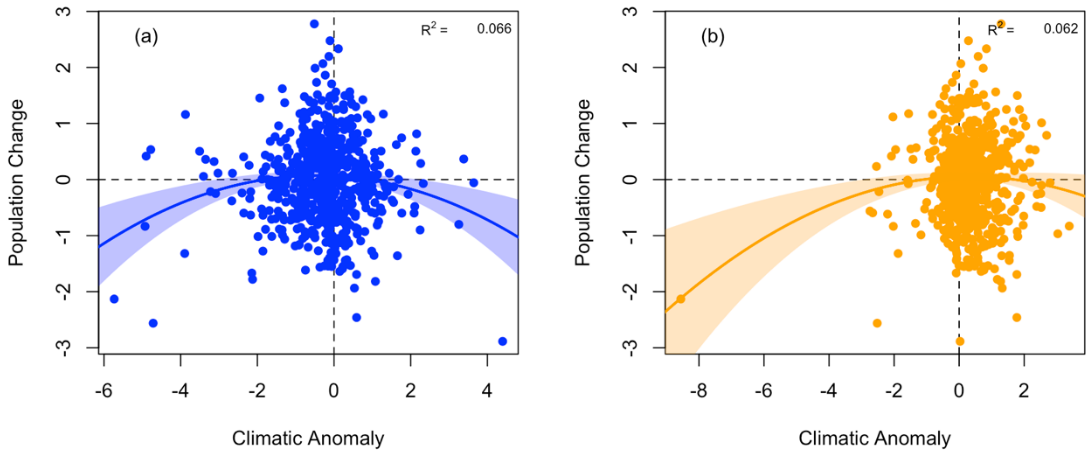

Supplementary Figure 13. Population change in relation to local and global climatic anomalies for (a) local and (b) global responses respectively for *Leptidea sinapis*, a species best adapted to local climatic anomalies in aridity during the post flight period of the previous year (*t-1*) of their adult stage (n = 1732). Local climatic anomalies refers to the deviations from the average conditions at the population site. Global climatic anomalies refers to the deviations from the average conditions across the species range. Colours indicate spatial scale (blue, local; orange, global), circles indicate raw data. R^2 values are provided. The degree of local adaptation dla = 0.004, with positive values indicating local adaptation. Adapted from Melero et al. 2022[1].

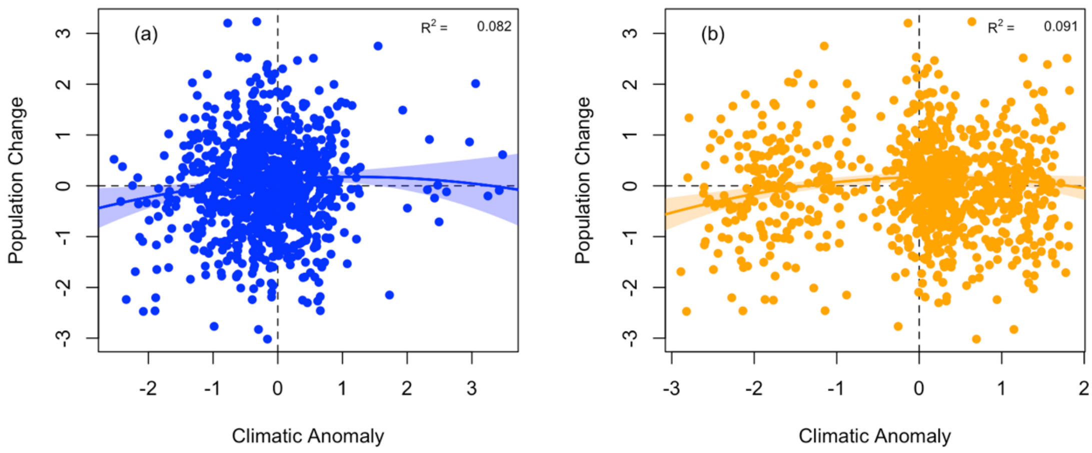

Supplementary Figure 14. Population change in relation to local and global climatic anomalies for (a) local and (b) global responses respectively for *Lycaena phlaeas*, a species best adapted to global climatic anomalies in temperature during the pre-flight period of the previous year (*t-1*) of their adult stage (n = 10049). Local climatic anomalies refers to the deviations from the average conditions at the population site. Global climatic anomalies refers to the deviations from the average conditions across the species range. Colours indicate spatial scale (blue, local; orange, global), circles indicate raw data. R^2 values are provided. The degree of local adaptation dla = -0.009, with negative values indicating global adaptation. Adapted from Melero et al. 2022[1].

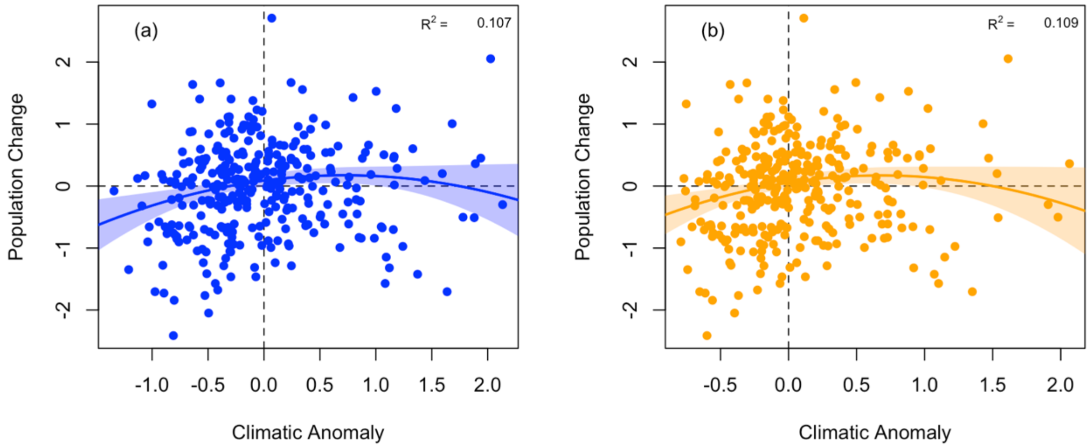

Supplementary Figure 15. Population change in relation to local and global climatic anomalies for (a) local and (b) global responses respectively for *Lycaena virgaureae*, a species best adapted to global climatic anomalies in precipitation during the flight period of the year (*t*) of their adult stage (n = 670). Local climatic anomalies refers to the deviations from the average conditions at the population site. Global climatic anomalies refers to the deviations from the average conditions across the species range. Colours indicate spatial scale (blue, local; orange, global), circles indicate raw data. R^2 values are provided. The degree of local adaptation dla = -0.002, with negative values indicating global adaptation. Adapted from Melero et al. 2022[1].

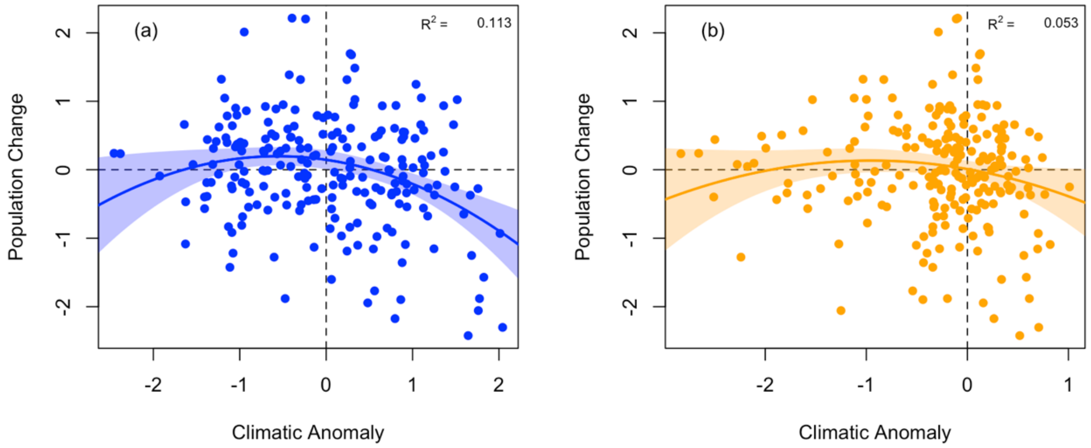

Supplementary Figure 16. Population change in relation to local and global climatic anomalies for (a) local and (b) global responses respectively for *Lysandra coridon*, a species best adapted to local climatic anomalies in temperature during the post flight period of the previous year (*t-1*) of their adult stage (n = 2323). Local climatic anomalies refers to the deviations from the average conditions at the population site. Global climatic anomalies refers to the deviations from the average conditions across the species range. Colours indicate spatial scale (blue, local; orange, global), circles indicate raw data. R^2 values are provided. The degree of local adaptation dla = 0.06, with positive values indicating local adaptation. Adapted from Melero et al. 2022[1].

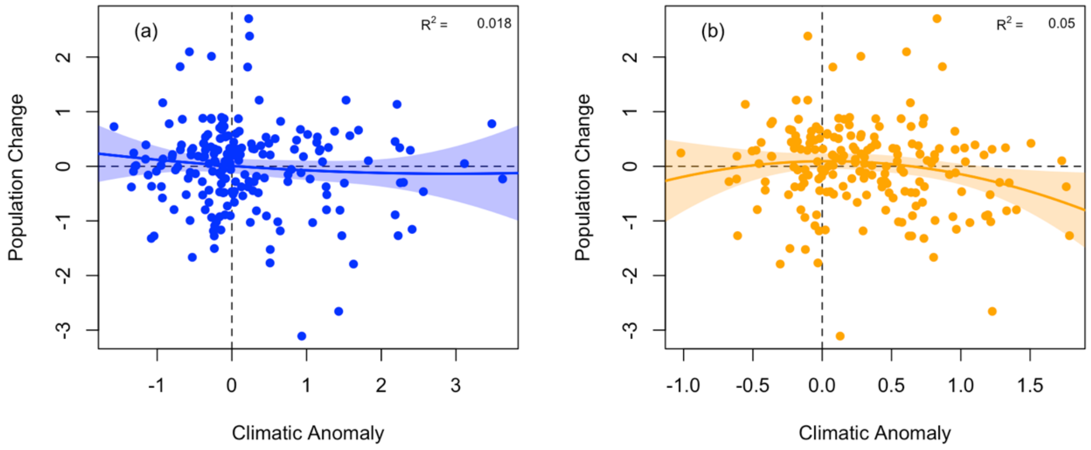

Supplementary Figure 17. Population change in relation to local and global climatic anomalies for (a) local and (b) global responses respectively for *Lysandra hispana*, a species best adapted to global climatic anomalies in aridity during the overwintering of the year (*t*) of their adult stage (n = 246). Local climatic anomalies refers to the deviations from the average conditions at the population site. Global climatic anomalies refers to the deviations from the average conditions across the species range. Colours indicate spatial scale (blue, local; orange, global), circles indicate raw data. R^2 values are provided. The degree of local adaptation dla = -0.032, with negative values indicating global adaptation. Adapted from Melero et al. 2022[1].

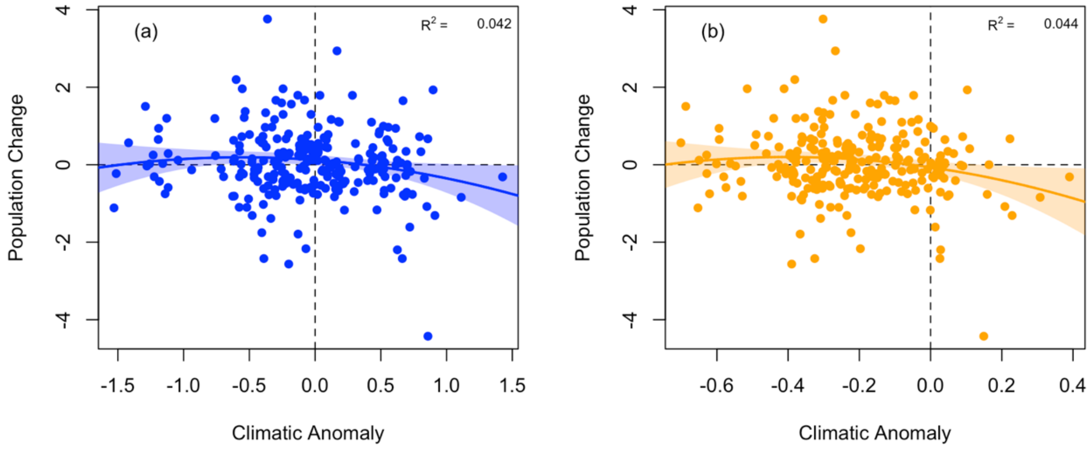

Supplementary Figure 18. Population change in relation to local and global climatic anomalies for (a) local and (b) global responses respectively for *Melanargia galathea*, a species best adapted to global climatic anomalies in temperature during the pre-flight period of the year (*t*) of their adult stage (n = 5948). Local climatic anomalies refers to the deviations from the average conditions at the population site. Global climatic anomalies refers to the deviations from the average conditions across the species range. Colours indicate spatial scale (blue, local; orange, global), circles indicate raw data. R^2 values are provided. The degree of local adaptation dla = -0.002, with negative values indicating global adaptation. Adapted from Melero et al. 2022[1].

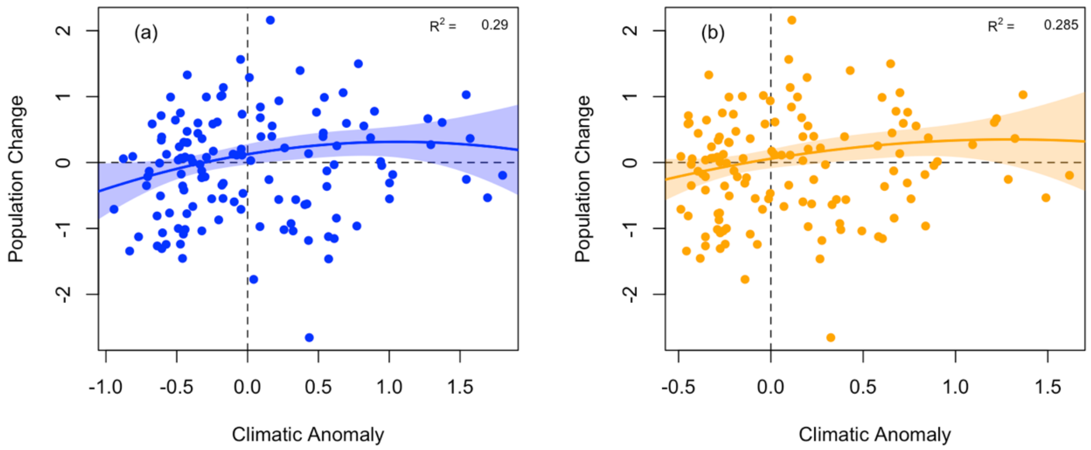

Supplementary Figure 19. Population change in relation to local and global climatic anomalies for (a) local and (b) global responses respectively for *Nymphalis polychloros*, a species best adapted to local climatic anomalies in precipitation during the pre-flight period of the previous year (*t-1*) of their adult stage (n = 350). Local climatic anomalies refers to the deviations from the average conditions at the population site. Global climatic anomalies refers to the deviations from the average conditions across the species range. Colours indicate spatial scale (blue, local; orange, global), circles indicate raw data. R^2 values are provided. The degree of local adaptation dla = 0.005, with positive values indicating local adaptation. Adapted from Melero et al. 2022[1].

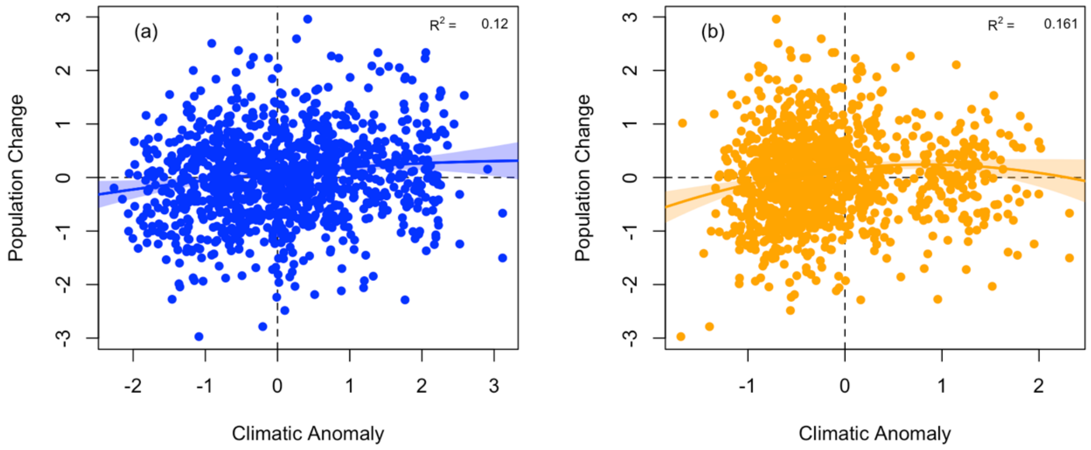

Supplementary Figure 20. Population change in relation to local and global climatic anomalies for (a) local and (b) global responses respectively for *Ochlodes sylvanus*, a species best adapted to global climatic anomalies in temperature during the flight period of the previous year (*t-1*) of their adult stage (n = 10070). Local climatic anomalies refers to the deviations from the average conditions at the population site. Global climatic anomalies refers to the deviations from the average conditions across the species range. Colours indicate spatial scale (blue, local; orange, global), circles indicate raw data. R^2 values are provided. The degree of local adaptation dla = -0.042, with negative values indicating global adaptation. Adapted from Melero et al. 2022[1].

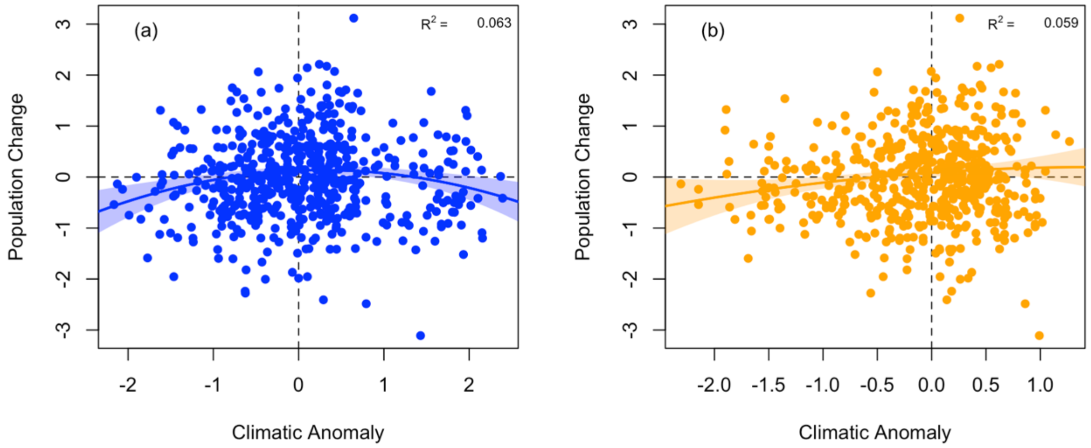

Supplementary Figure 21. Population change in relation to local and global climatic anomalies for (a) local and (b) global responses respectively for *Papilio machaon*, a species best adapted to local climatic anomalies in temperature during the flight period of the year (*t*) of their adult stage (n = 856). Local climatic anomalies refers to the deviations from the average conditions at the population site. Global climatic anomalies refers to the deviations from the average conditions across the species range. Colours indicate spatial scale (blue, local; orange, global), circles indicate raw data. R^2 values are provided. The degree of local adaptation dla = 0.004, with positive values indicating local adaptation. Adapted from Melero et al. 2022[1].

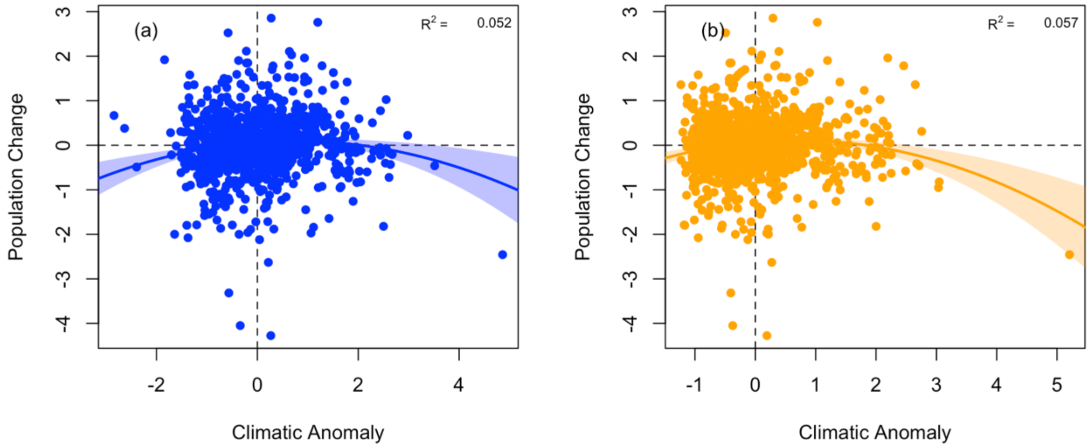

Supplementary Figure 22. Population change in relation to local and global climatic anomalies for (a) local and (b) global responses respectively for *Pararge aegeria*, a species best adapted to global climatic anomalies in precipitation during the post flight period of the previous year (*t-1*) of their adult stage (n = 10070). Local climatic anomalies refers to the deviations from the average conditions at the population site. Global climatic anomalies refers to the deviations from the average conditions across the species range. Colours indicate spatial scale (blue, local; orange, global), circles indicate raw data. R^2 values are provided. The degree of local adaptation dla = -0.005, with negative values indicating global adaptation. Adapted from Melero et al. 2022[1].

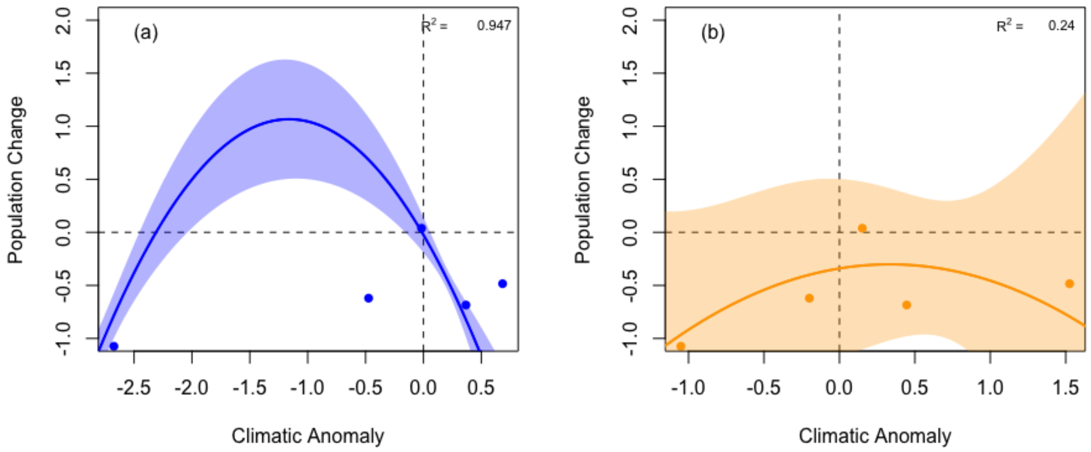

Supplementary Figure 23. Population change in relation to local and global climatic anomalies for (a) local* and (b) global responses respectively for *Parnassius apollo*, a species best adapted to local climatic anomalies in precipitation during the pre-flight period of the previous year (*t-1*) of their adult stage (n = 12). Local climatic anomalies refers to the deviations from the average conditions at the population site. Global climatic anomalies refers to the deviations from the average conditions across the species range. Colours indicate spatial scale (blue, local; orange, global), circles indicate raw data. R^2 values are provided. The degree of local adaptation dla = 0.707, with positive values indicating local adaptation. Adapted from Melero et al. 2022[1]. *Resulting model fit is due to the shrinkage to the average estimate of the climatic anomaly and its quadratic. All models in MS were also tested without *Parnassius apollo.*

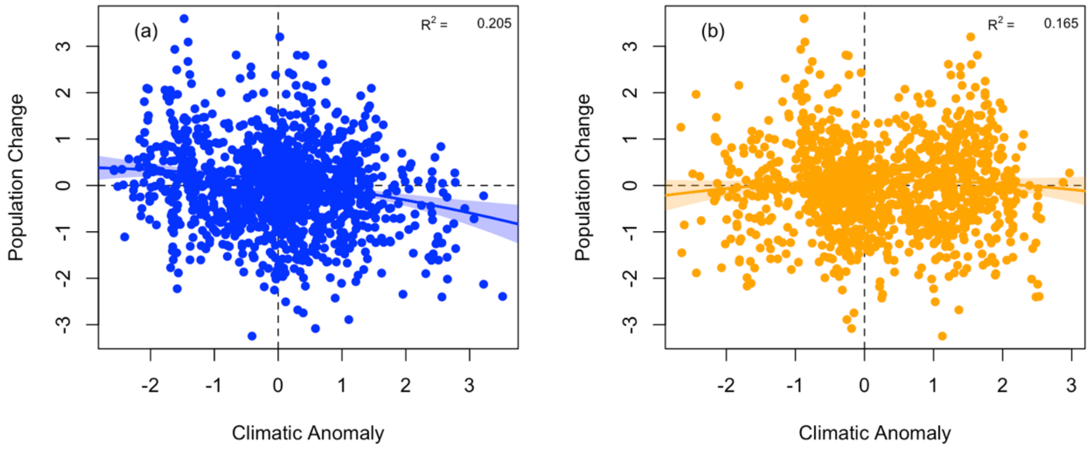

Supplementary Figure 24. Population change in relation to local and global climatic anomalies for (a) local and (b) global responses respectively for *Pieris brassicae*, a species best adapted to local climatic anomalies in temperature during the post flight period of the previous year (*t-1*) of their adult stage (n = 11110). Local climatic anomalies refers to the deviations from the average conditions at the population site. Global climatic anomalies refers to the deviations from the average conditions across the species range. Colours indicate spatial scale (blue, local; orange, global), circles indicate raw data. R^2 values are provided. The degree of local adaptation dla = 0.04, with positive values indicating local adaptation. Adapted from Melero et al. 2022[1].

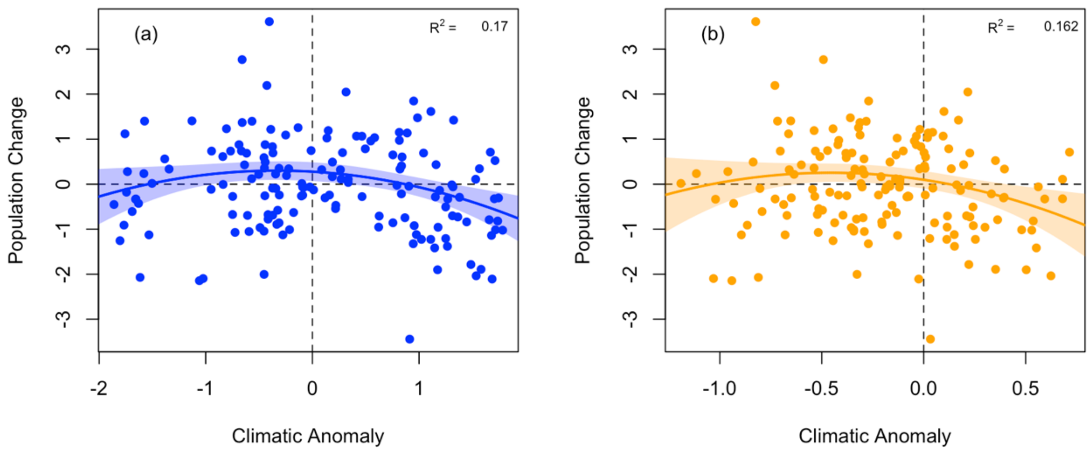

Supplementary Figure 25. Population change in relation to local and global climatic anomalies for (a) local and (b) global responses respectively for *Plebejus idas*, a species best adapted to local climatic anomalies in temperature during the post flight period of the previous year (*t-1*) of their adult stage (n = 428). Local climatic anomalies refers to the deviations from the average conditions at the population site. Global climatic anomalies refers to the deviations from the average conditions across the species range. Colours indicate spatial scale (blue, local; orange, global), circles indicate raw data. R^2 values are provided. The degree of local adaptation dla = 0.008, with positive values indicating local adaptation. Adapted from Melero et al. 2022[1].

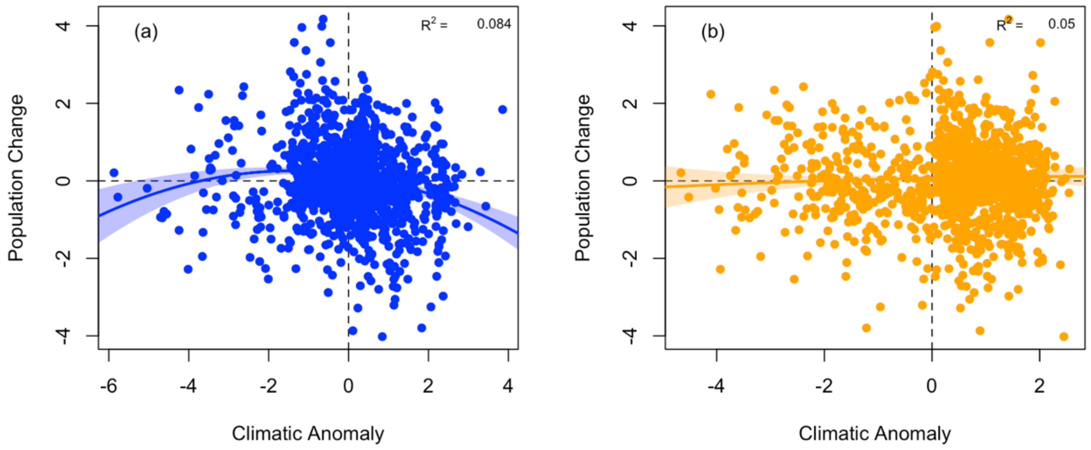

Supplementary Figure 26. Population change in relation to local and global climatic anomalies for (a) local and (b) global responses respectively for *Polyommatus icarus*, a species best adapted to local climatic anomalies in temperature during the overwintering of the previous year (*t-1*) of their adult stage (n = 10859). Local climatic anomalies refers to the deviations from the average conditions at the population site. Global climatic anomalies refers to the deviations from the average conditions across the species range. Colours indicate spatial scale (blue, local; orange, global), circles indicate raw data. R^2 values are provided. The degree of local adaptation dla = 0.034, with positive values indicating local adaptation. Adapted from Melero et al. 2022[1].

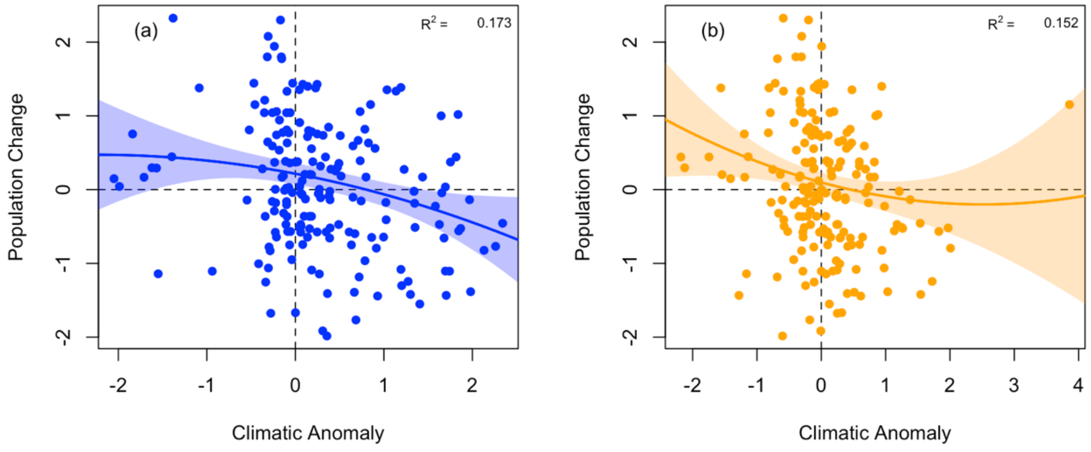

Supplementary Figure 27. Population change in relation to local and global climatic anomalies for (a) local and (b) global responses respectively for *Pseudophilotes panoptes*, a species best adapted to local climatic anomalies in aridity during the pre-flight period of the year (*t*) of their adult stage (n = 337). Local climatic anomalies refers to the deviations from the average conditions at the population site. Global climatic anomalies refers to the deviations from the average conditions across the species range. Colours indicate spatial scale (blue, local; orange, global), circles indicate raw data. R^2 values are provided. The degree of local adaptation dla = 0.022, with positive values indicating local adaptation. Adapted from Melero et al. 2022[1].

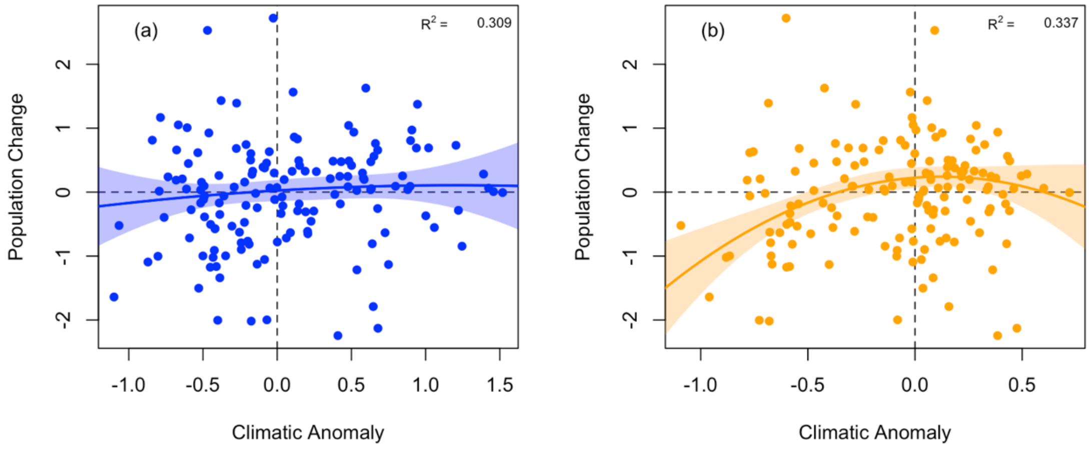

Supplementary Figure 28. Population change in relation to local and global climatic anomalies for (a) local and (b) global responses respectively for *Pyrgus malvae*, a species best adapted to global climatic anomalies in temperature during the post flight period of the previous year (*t-1*) of their adult stage (n = 3072). Local climatic anomalies refers to the deviations from the average conditions at the population site. Global climatic anomalies refers to the deviations from the average conditions across the species range. Colours indicate spatial scale (blue, local; orange, global), circles indicate raw data. R^2 values are provided. The degree of local adaptation dla = -0.028, with negative values indicating global adaptation. Adapted from Melero et al. 2022[1].

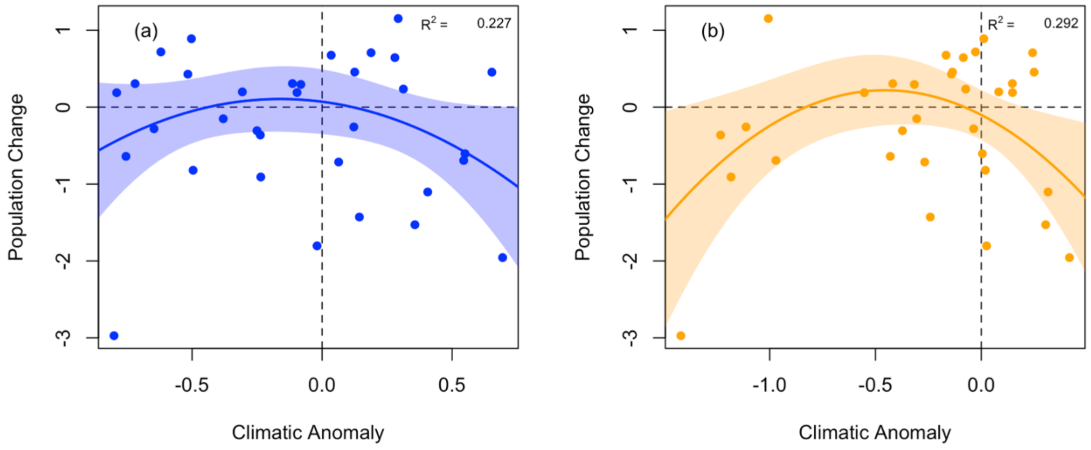

Supplementary Figure 29. Population change in relation to local and global climatic anomalies for (a) local and (b) global responses respectively for *Satyrium acaciae*, a species best adapted to global climatic anomalies in temperature during the pre-flight period of the previous year (*t-1*) of their adult stage (n = 64). Local climatic anomalies refers to the deviations from the average conditions at the population site. Global climatic anomalies refers to the deviations from the average conditions across the species range. Colours indicate spatial scale (blue, local; orange, global), circles indicate raw data. R^2 values are provided. The degree of local adaptation dla = -0.065, with negative values indicating global adaptation. Adapted from Melero et al. 2022[1].

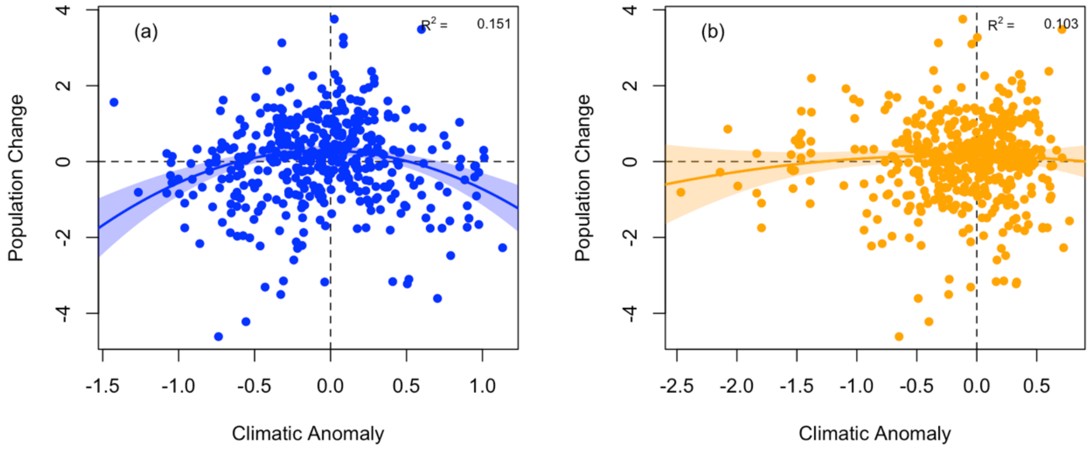

Supplementary Figure 30. Population change in relation to local and global climatic anomalies for (a) local and (b) global responses respectively for *Satyrium esculi*, a species best adapted to local climatic anomalies in temperature during the pre-flight period of the previous year (*t-1*) of their adult stage (n = 568). Local climatic anomalies refers to the deviations from the average conditions at the population site. Global climatic anomalies refers to the deviations from the average conditions across the species range. Colours indicate spatial scale (blue, local; orange, global), circles indicate raw data. R^2 values are provided. The degree of local adaptation dla = 0.048, with positive values indicating local adaptation. Adapted from Melero et al. 2022[1]

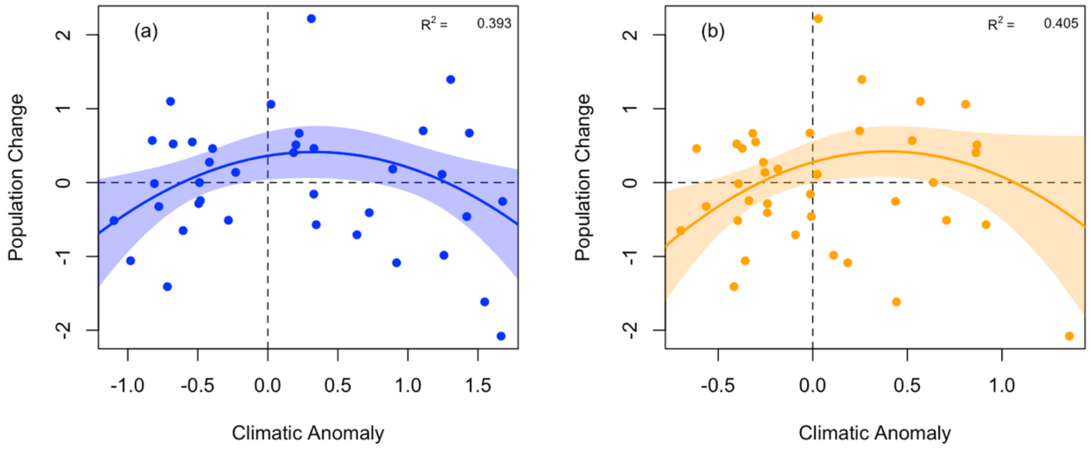

Supplementary Figure 31. Population change in relation to local and global climatic anomalies for (a) local and (b) global responses respectively for *Satyrium pruni*, a species best adapted to global climatic anomalies in temperature during the post flight period of the previous year (*t-1*) of their adult stage (n = 190). Local climatic anomalies refers to the deviations from the average conditions at the population site. Global climatic anomalies refers to the deviations from the average conditions across the species range. Colours indicate spatial scale (blue, local; orange, global), circles indicate raw data. R^2 values are provided. The degree of local adaptation dla = -0.012, with negative values indicating global adaptation. Adapted from Melero et al. 2022[1].

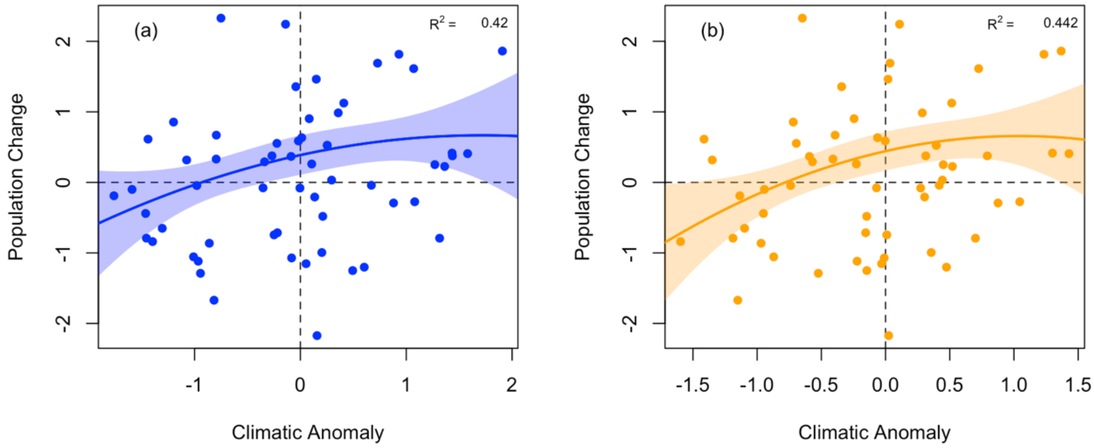

Supplementary Figure 32. Population change in relation to local and global climatic anomalies for (a) local and (b) global responses respectively for *Satyrium spini*, a species best adapted to global climatic anomalies in precipitation during the post flight period of the previous year (*t-1*) of their adult stage (n = 139). Local climatic anomalies refers to the deviations from the average conditions at the population site. Global climatic anomalies refers to the deviations from the average conditions across the species range. Colours indicate spatial scale (blue, local; orange, global), circles indicate raw data. R^2 values are provided. The degree of local adaptation dla = -0.022, with negative values indicating global adaptation. Adapted from Melero et al. 2022[1].

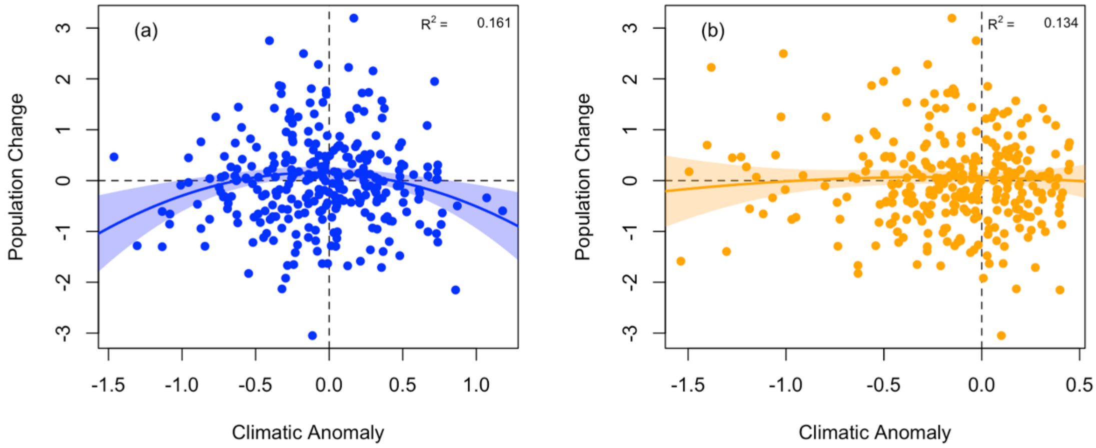

Supplementary Figure 33. Population change in relation to local and global climatic anomalies for (a) local and (b) global responses respectively for *Thymelicus acteon*, a species best adapted to local climatic anomalies in temperature during the pre-flight period of the previous year (*t-1*) of their adult stage (n = 657). Local climatic anomalies refers to the deviations from the average conditions at the population site. Global climatic anomalies refers to the deviations from the average conditions across the species range. Colours indicate spatial scale (blue, local; orange, global), circles indicate raw data. R^2 values are provided. The degree of local adaptation dla = 0.027, with positive values indicating local adaptation. Adapted from Melero et al. 2022[1].

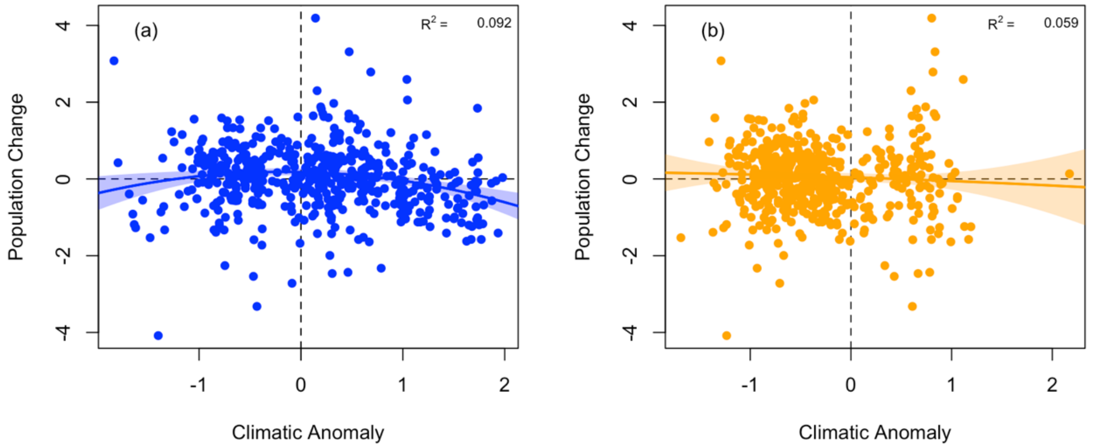

Supplementary Figure 34. Population change in relation to local and global climatic anomalies for (a) local and (b) global responses respectively for *Thymelicus lineola*, a species best adapted to local climatic anomalies in temperature during the post flight period of the previous year (*t-1*) of their adult stage (n = 5033). Local climatic anomalies refers to the deviations from the average conditions at the population site. Global climatic anomalies refers to the deviations from the average conditions across the species range. Colours indicate spatial scale (blue, local; orange, global), circles indicate raw data. R^2 values are provided. The degree of local adaptation dla = 0.033, with positive values indicating local adaptation. Adapted from Melero et al. 2022[1]

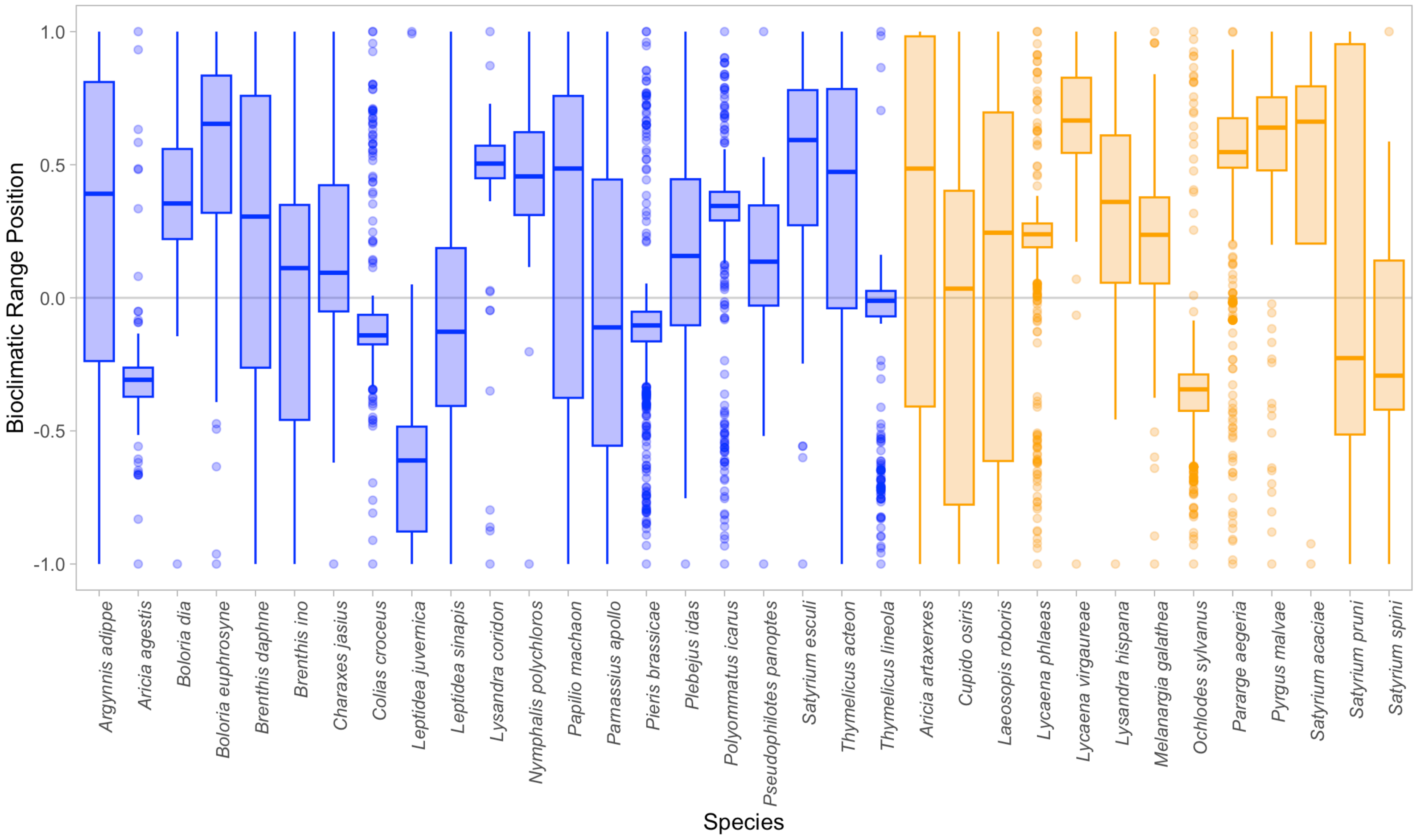

Supplementary Figure 35. Boxplot of the bioclimatic range position of the studied populations calculated per species independently, ranging [-1, 1] from the leading to the trailing margins. Grey line indicates the centre of the species range. Colours indicate locally for blue and globally adapted species for orange.

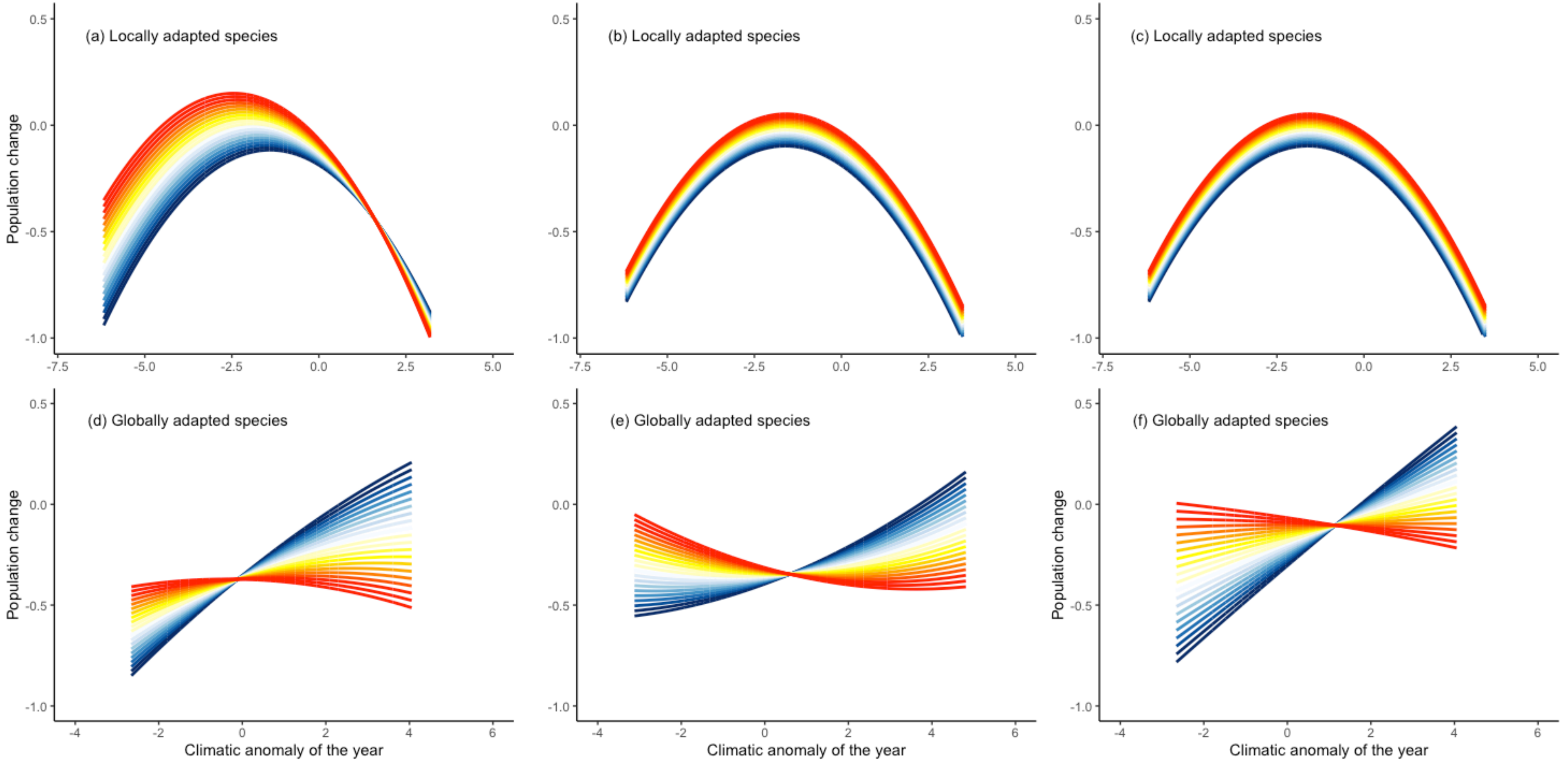

Supplementary Figure 36. Population change in relation to the local climatic anomalies of the year and the population position within the species bioclimatic range. A conservative approach was taken, with the species selection based on two different thresholds of their degree of local adaptation (dla) from less to more restrictive, and the inclusion or exclusion of potential species outliers. Panels **a**, **d** relate to locally and globally adapted species ($N_{species\ local} = 20$, $N_{species\ global} = 11$), respectively, with dla range 0 to | 1 |, but removing potential outliers; **b**, **e** relate to locally and globally adapted species ($N_{species\ local} = 12$, $N_{species\ global} = 6$), respectively, with dla range |0.025| to | 1 |; and **c**, **f** relate to locally and globally adapted species ($N_{species\ local} = 11$, $N_{species\ global} = 4$), respectively, with dla range |0.025| to | 1 |, and removing potential outliers. Potential outliers were identified based on the species dla: *Parnassius apollo* (dla = 0.7) , *Laeosopis roboris* (dla = -0.22) and *Cupido osiris* (dla = -0.23; Supplementary Fig. 72 and Supplementary Table 4). Climatic anomaly relates to the variable most affecting the species (temperature, precipitation or aridity[1]; Supplementary Figs. 1-34). Divergent responses according to the position of the site in the species bioclimatic range are shown at 0.1 intervals from the leading (range position = -1) to the trailing margin (range position = 1), displayed from the leading to training (blue and red, respectively; white indicates centre).

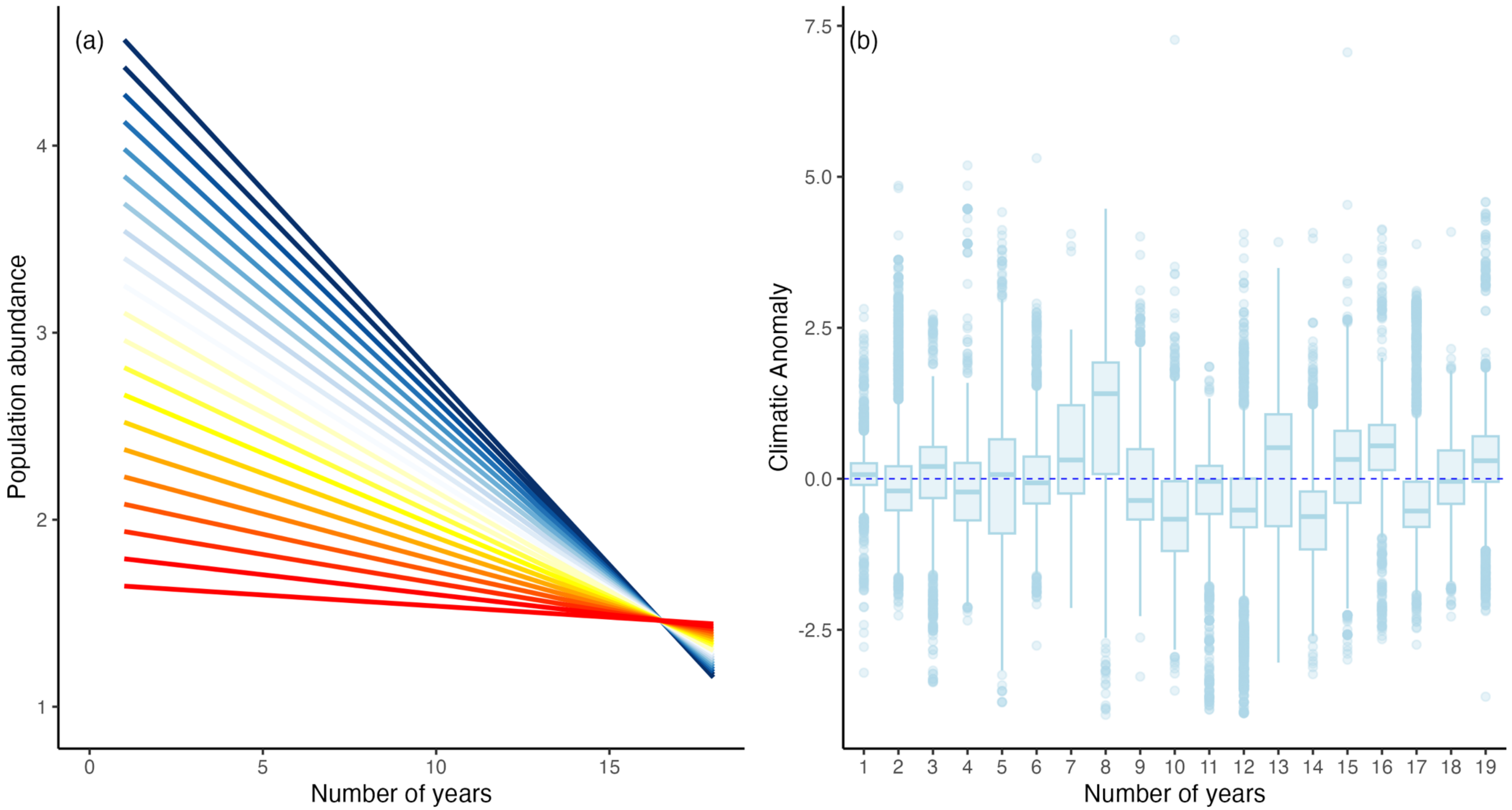

Supplementary Figure 37. Population abundance (log scale) and climatic anomalies over time for *Argynnis adippe*, a species best adapted to local climatic anomalies in precipitation during the pre-flight period of the previous year (t-1) of their adult stage. Panel **a** shows population abundance over time in relation to the population position within the species bioclimatic range (Supplementary Fig. 35). Divergent responses according to the position of the site in the species bioclimatic range are shown at 0.1 intervals from the leading (range position = -1) to the trailing margin (range position = 1), displayed from the leading to trailing (blue and red, respectively; white indicates centre). Panel b shows the boxplot of the local climatic anomalies over time for the species. Dashed line related to the lack of climatic anomalies.

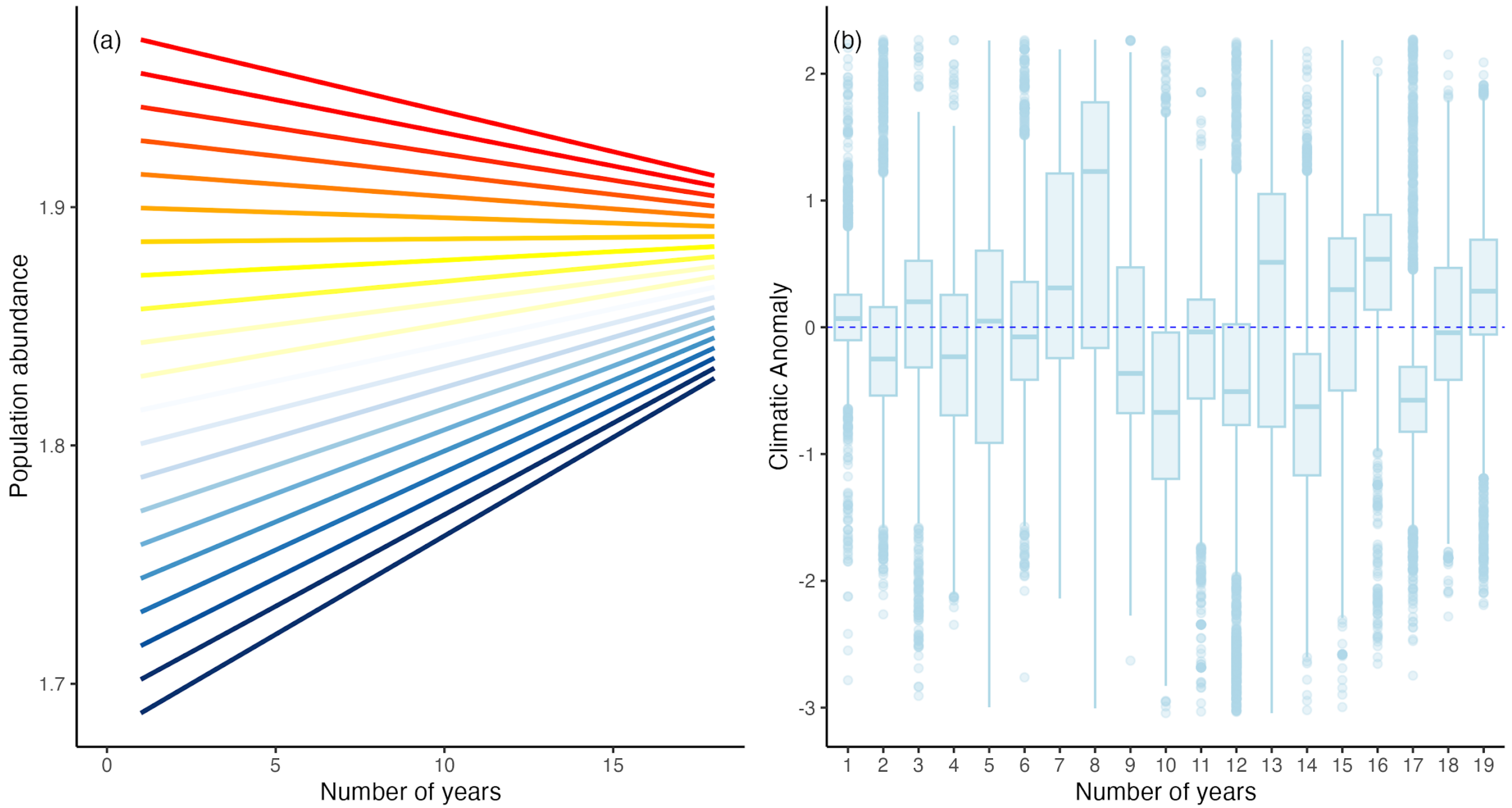

Supplementary Figure 38. Population abundance (log scale) and climatic anomalies over time for *Aricia agestis*, a species best adapted to local climatic anomalies in temperature during the flight period of the previous year (t-1) of their adult stage. Panel **a** shows population abundance over time in relation to the population position within the species bioclimatic range (Supplementary Fig. 35). Divergent responses according to the position of the site in the species bioclimatic range are shown at 0.1 intervals from the leading (range position = -1) to the trailing margin (range position = 1), displayed from the leading to trailing (blue and red, respectively; white indicates centre). Panel **b** shows the boxplot of the local climatic anomalies over time for the species. Dashed line related to the lack of climatic anomalies.

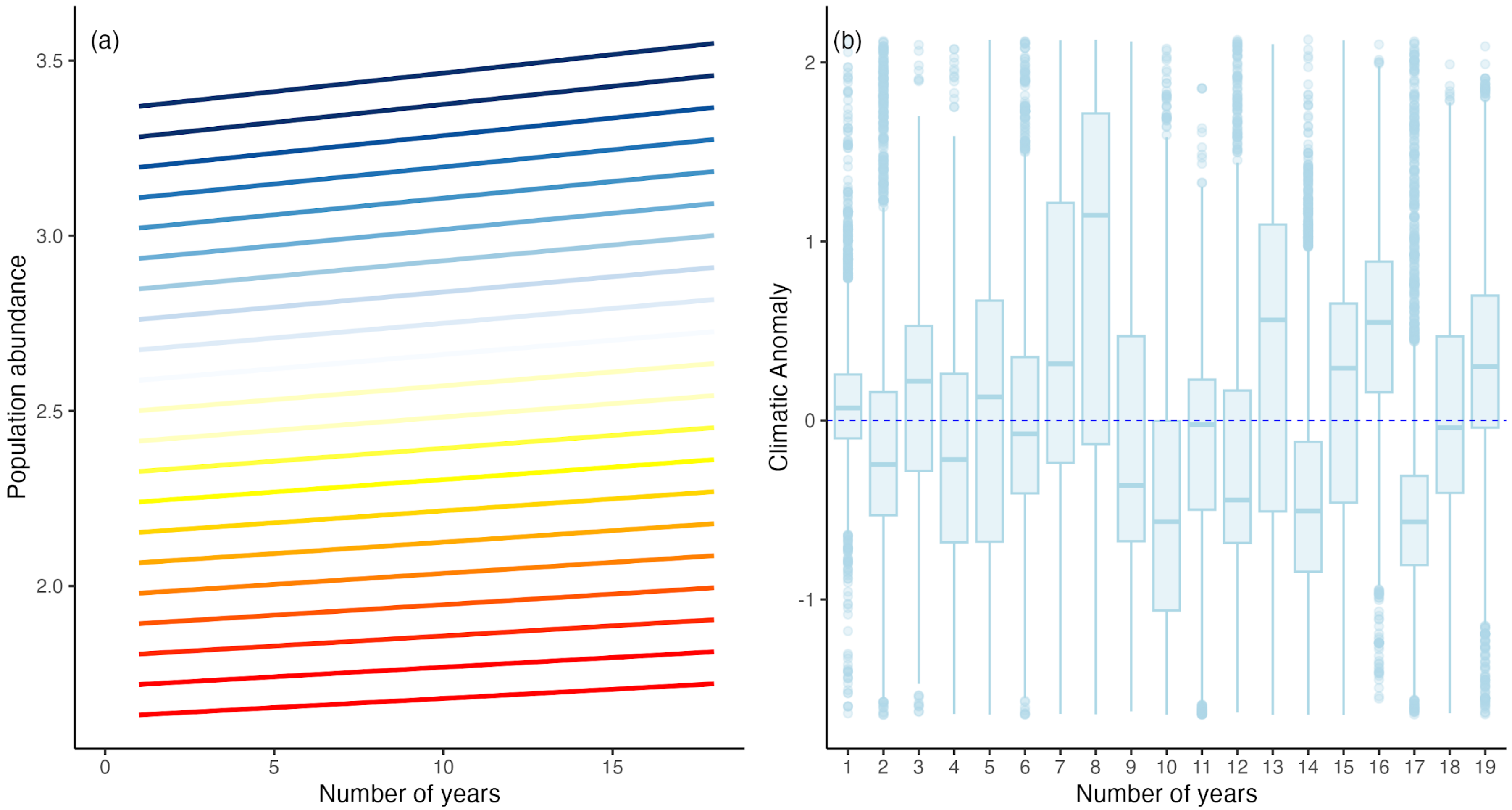

Supplementary Figure 39. Population abundance (log scale) and climatic anomalies over time for *Boloria dia*, a species best adapted to local climatic anomalies in precipitation during the post-flight period of the previous year (t-1) of their adult stage. Panel **a** shows population abundance over time in relation to the population position within the species bioclimatic range (Supplementary Fig. 35). Divergent responses according to the position of the site in the species bioclimatic range are shown at 0.1 intervals from the leading (range position = -1) to the trailing margin (range position = 1), displayed from the leading to trailing (blue and red, respectively; white indicates centre). Panel **b** shows the boxplot of the local climatic anomalies over time for the species. Dashed line related to the lack of climatic anomalies.

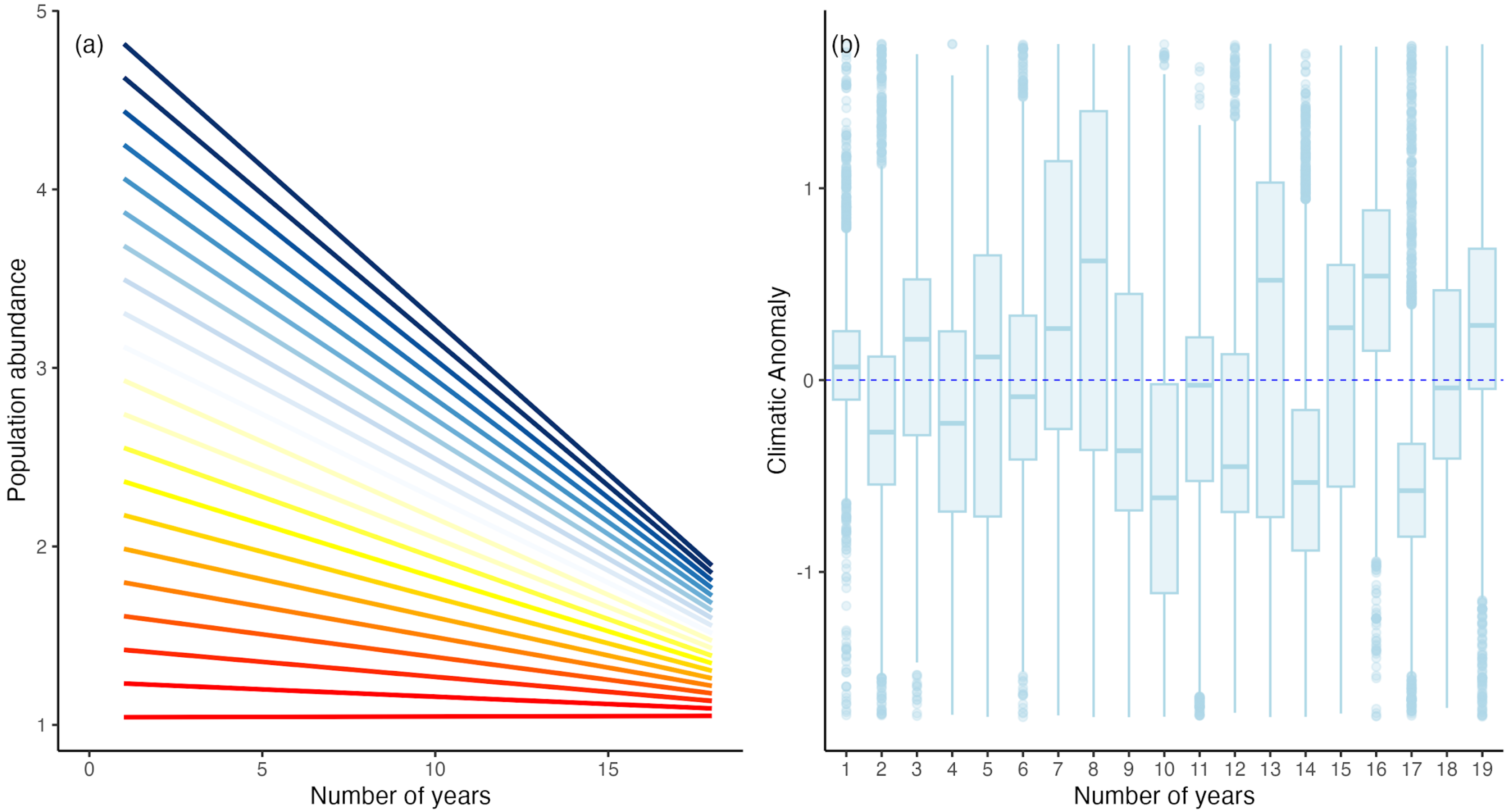

Supplementary Figure 40. Population abundance (log scale) and climatic anomalies over time for *Boloria euphrosyne*, a species best adapted to local climatic anomalies in precipitation during the flight period of the year (t) of their adult stage. Panel **a** shows population abundance over time in relation to the population position within the species bioclimatic range (Supplementary Fig. 35). Divergent responses according to the position of the site in the species bioclimatic range are shown at 0.1 intervals from the leading (range position = -1) to the trailing margin (range position = 1), displayed from the leading to trailing (blue and red, respectively; white indicates centre). Panel **b** shows the boxplot of the local climatic anomalies over time for the species. Dashed line related to the lack of climatic anomalies.

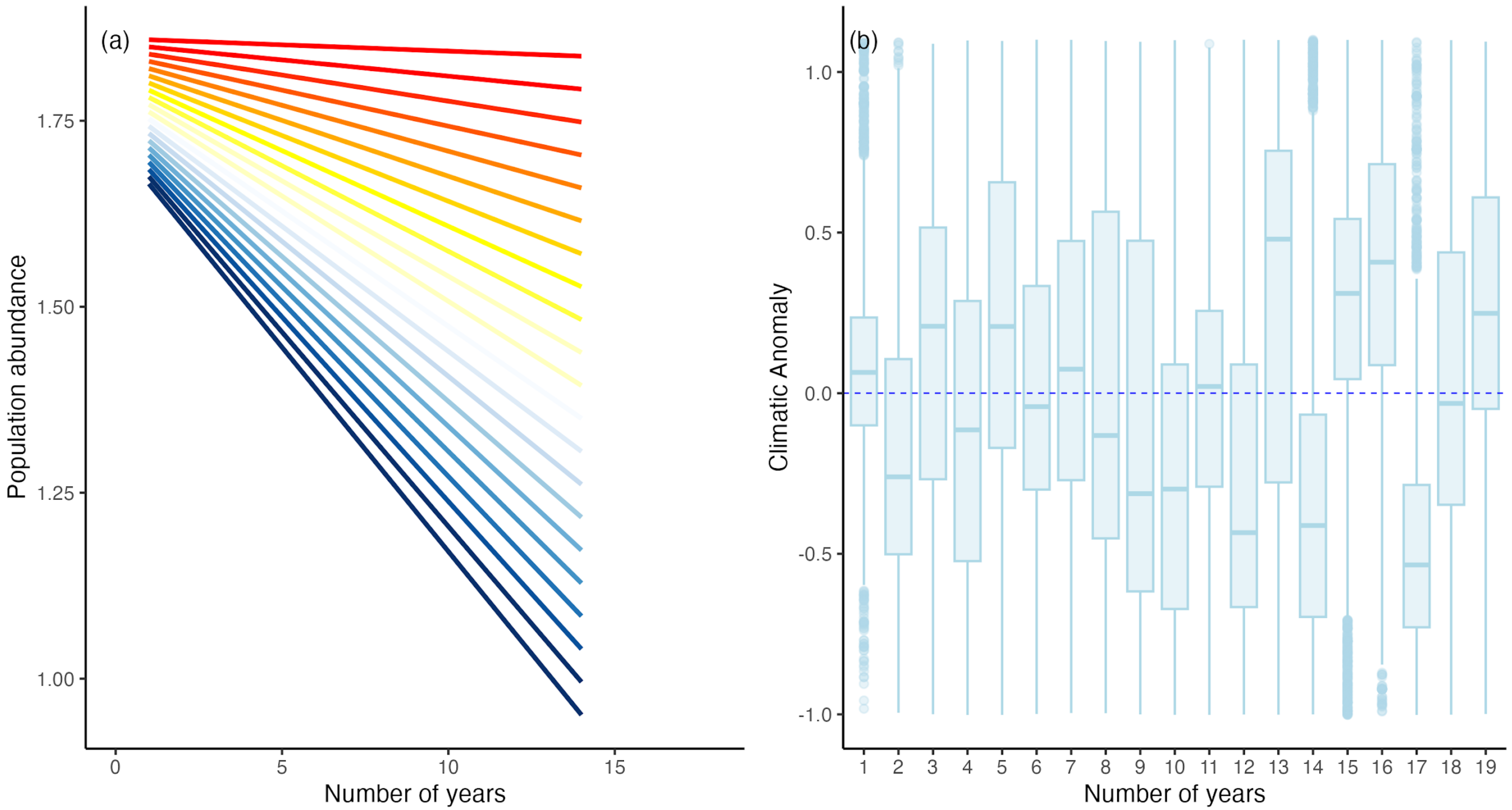

Supplementary Figure 41. Population abundance (log scale) and climatic anomalies over time for *Brenthis daphne*, a species best adapted to local climatic anomalies in precipitation during the flight period of the year (t) of their adult stage. Panel **a** shows population abundance over time in relation to the population position within the species bioclimatic range (Supplementary Fig. 35). Divergent responses according to the position of the site in the species bioclimatic range are shown at 0.1 intervals from the leading (range position = -1) to the trailing margin (range position = 1), displayed from the leading to trailing (blue and red, respectively; white indicates centre). Panel **b** shows the boxplot of the local climatic anomalies over time for the species. Dashed line related to the lack of climatic anomalies.

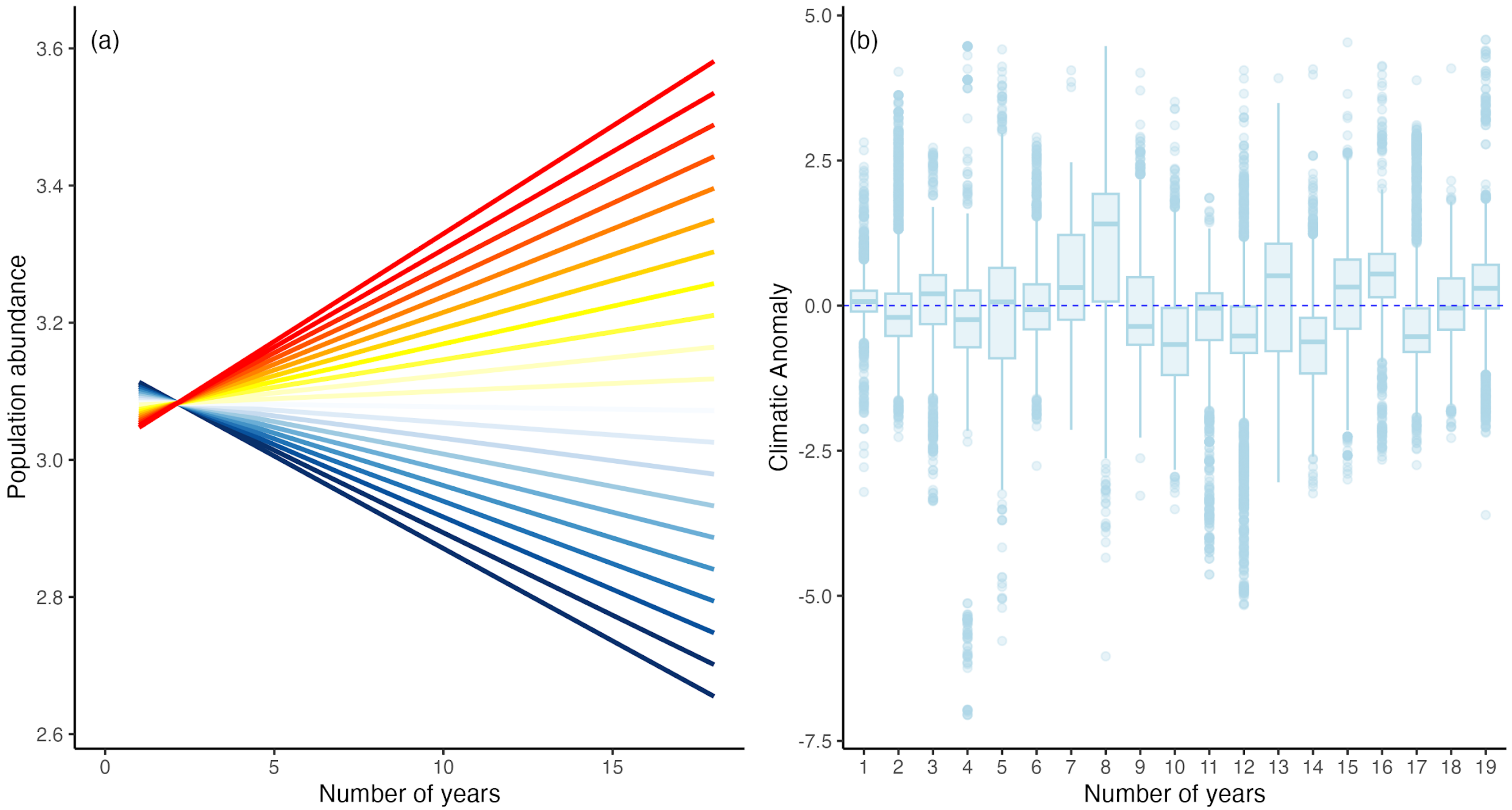

Supplementary Figure 42. Population abundance (log scale) and climatic anomalies over time for *Brenthis ino*, a species best adapted to local climatic anomalies in temperature during the OW period of the previous year (t-1) of their adult stage. Panel **a** shows population abundance over time in relation to the population position within the species bioclimatic range (Supplementary Fig. 35). Divergent responses according to the position of the site in the species bioclimatic range are shown at 0.1 intervals from the leading (range position = -1) to the trailing margin (range position = 1), displayed from the leading to trailing (blue and red, respectively; white indicates centre). Panel **b** shows the boxplot of the local climatic anomalies over time for the species. Dashed line related to the lack of climatic anomalies.

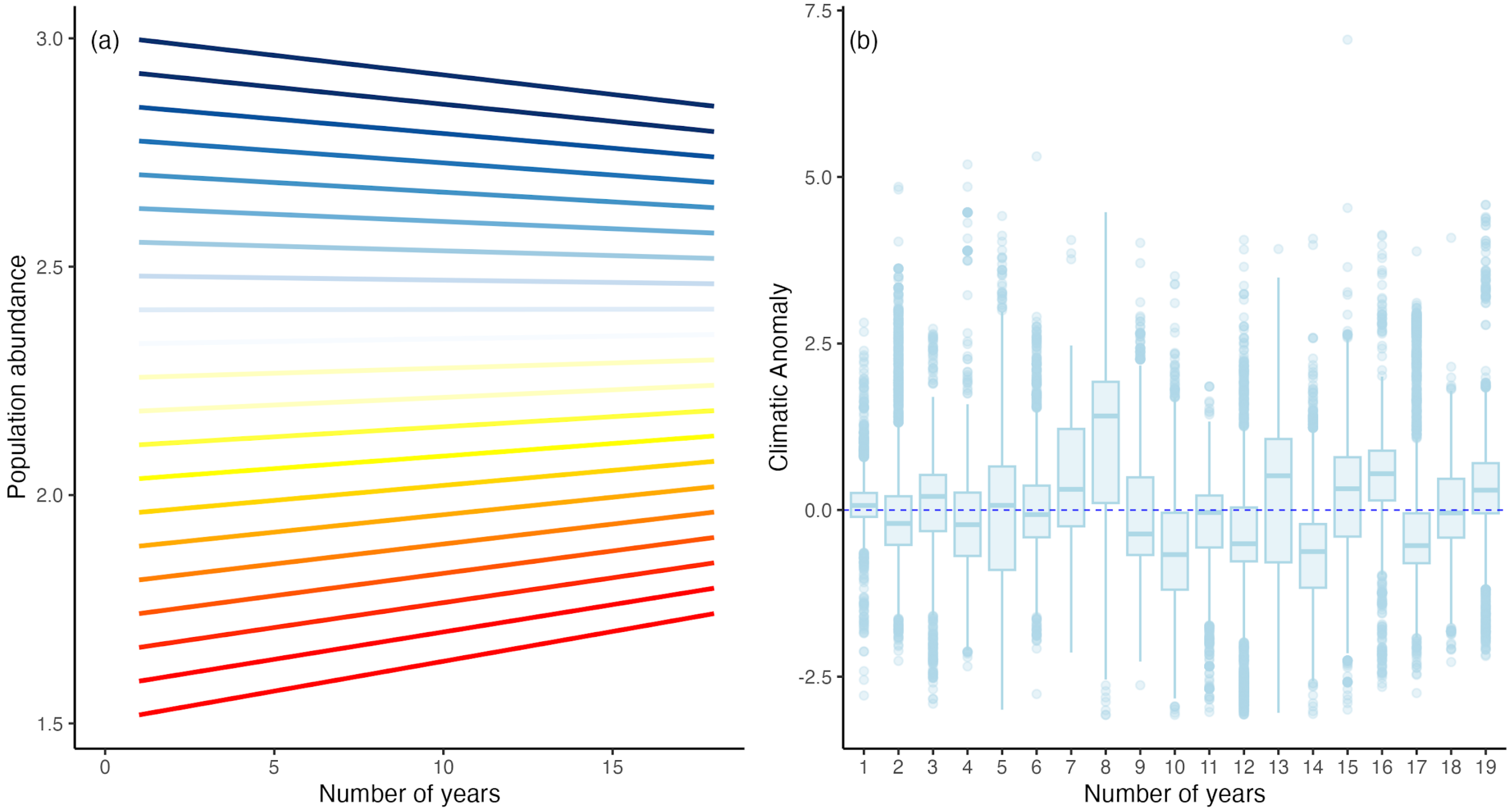

Supplementary Figure 43. Population abundance (log scale) and climatic anomalies over time for *Charaxes jasius*, a species best adapted to local climatic anomalies in precipitation during the pre-flight period of the previous year (t-1) of their adult stage. Panel **a** shows population abundance over time in relation to the population position within the species bioclimatic range (Supplementary Fig. 35). Divergent responses according to the position of the site in the species bioclimatic range are shown at 0.1 intervals from the leading (range position = -1) to the trailing margin (range position = 1), displayed from the leading to trailing (blue and red, respectively; white indicates centre). Panel **b** shows the boxplot of the local climatic anomalies over time for the species. Dashed line related to the lack of climatic anomalies.

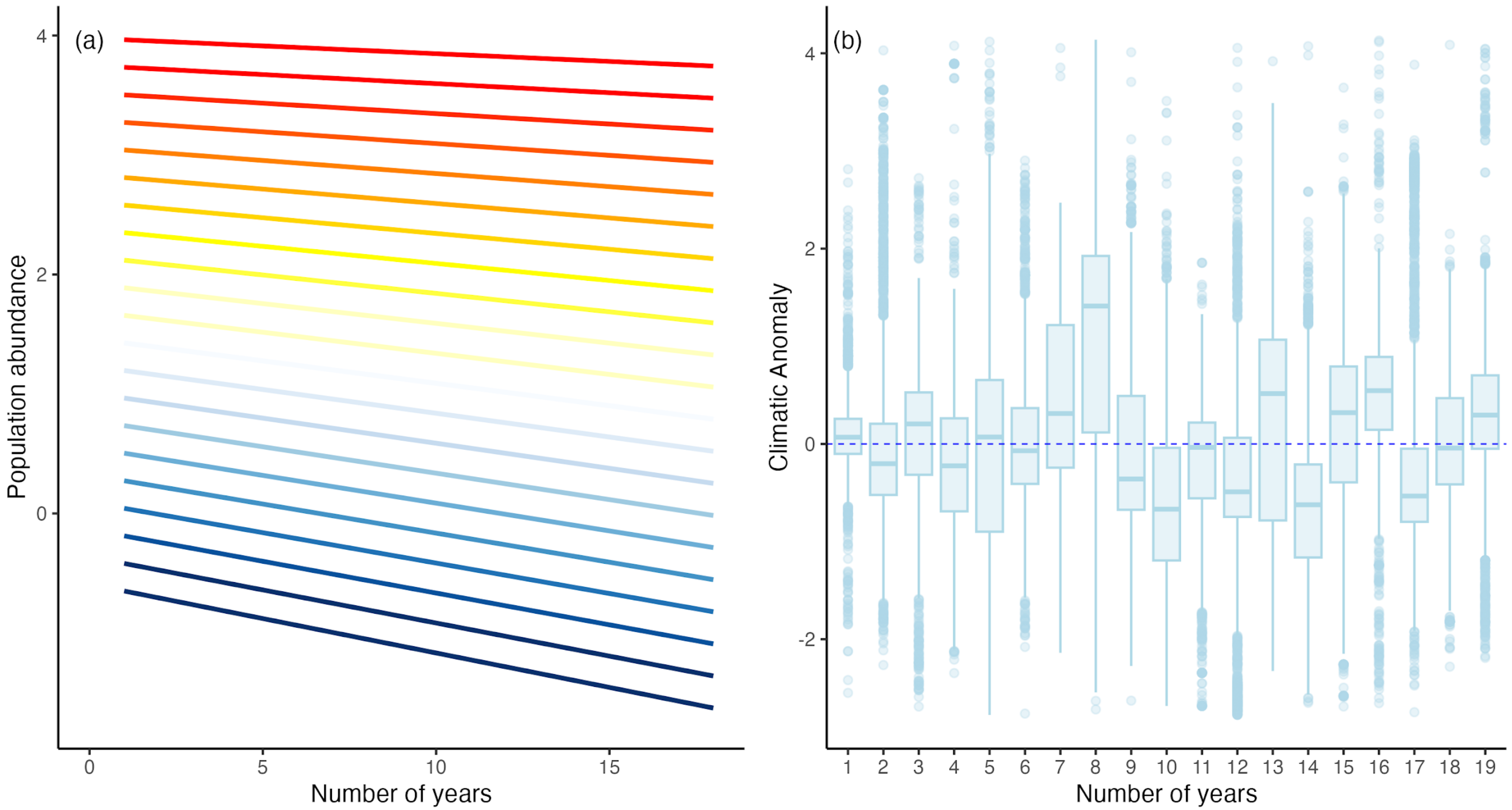

Supplementary Figure 44. Population abundance (log scale) and climatic anomalies over time for *Colias croceus*, a species best adapted to local climatic anomalies in temperature during the post-flight period of the previous year (t-1) of their adult stage. Panel **a** shows population abundance over time in relation to the population position within the species bioclimatic range (Supplementary Fig. 35). Divergent responses according to the position of the site in the species bioclimatic range are shown at 0.1 intervals from the leading (range position = -1) to the trailing margin (range position = 1), displayed from the leading to trailing (blue and red, respectively; white indicates centre). Panel **b** shows the boxplot of the local climatic anomalies over time for the species. Dashed line related to the lack of climatic anomalies.

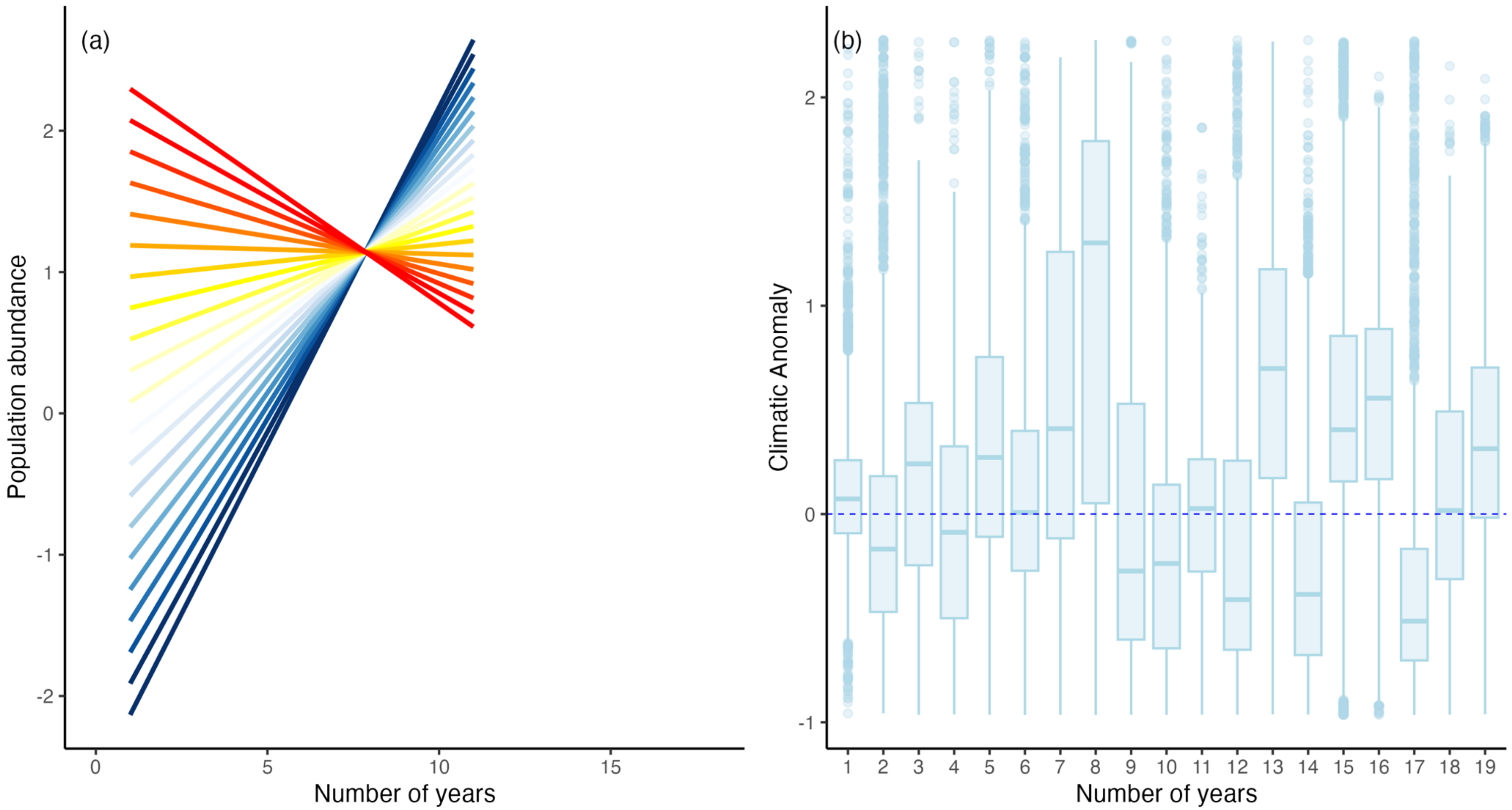

Supplementary Figure 45. Population abundance (log scale) and climatic anomalies over time for *Leptidea juvernica*, a species best adapted to local climatic anomalies in precipitation during the flight period of the previous year (t-1) of their adult stage. Panel **a** shows population abundance over time in relation to the population position within the species bioclimatic range (Supplementary Fig. 35). Divergent responses according to the position of the site in the species bioclimatic range are shown at 0.1 intervals from the leading (range position = -1) to the trailing margin (range position = 1), displayed from the leading to trailing (blue and red, respectively; white indicates centre). Panel **b** shows the boxplot of the local climatic anomalies over time for the species. Dashed line related to the lack of climatic anomalies.

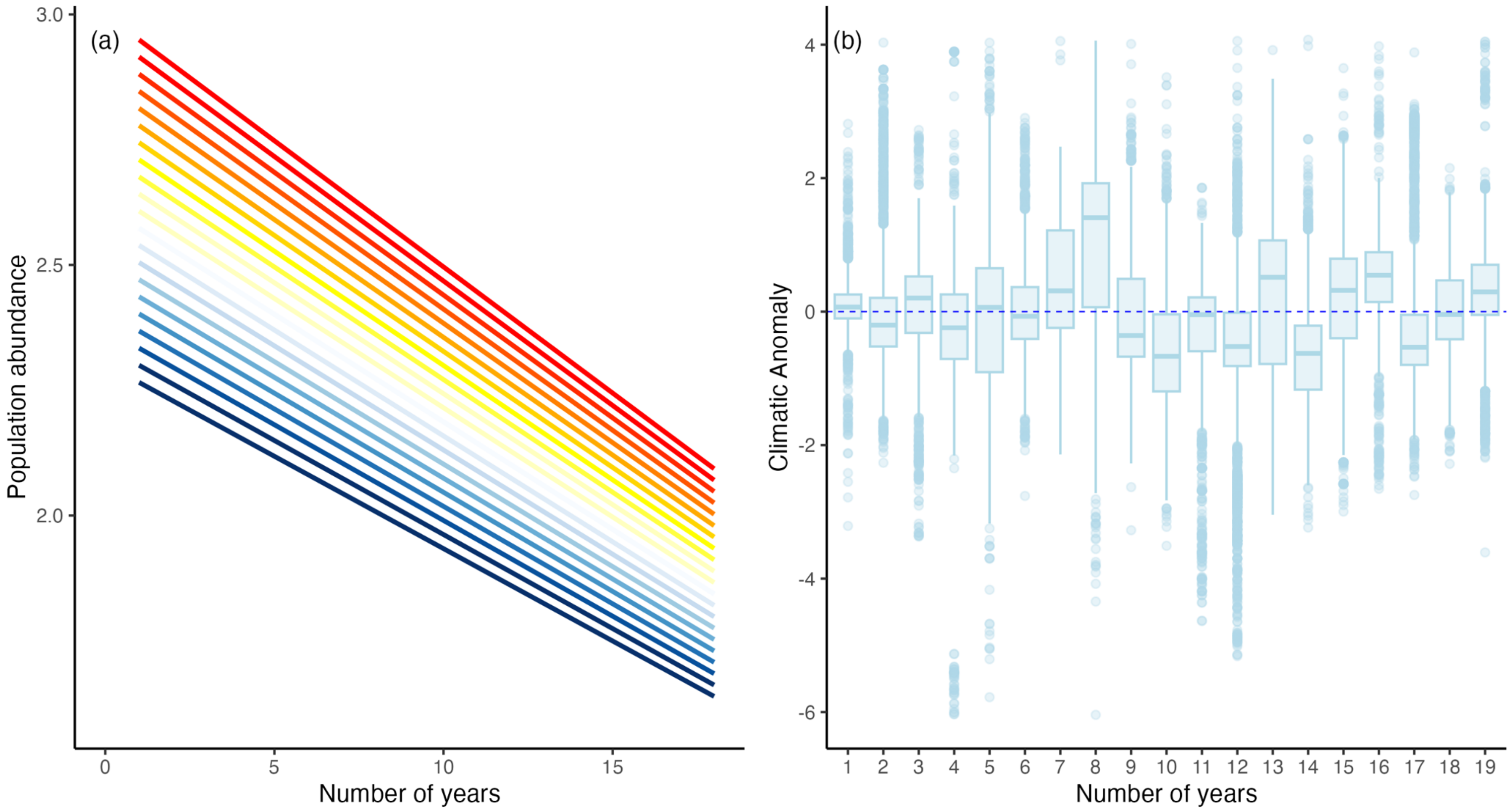

Supplementary Figure 46. Population abundance (log scale) and climatic anomalies over time for *Leptidea sinapis*, a species best adapted to local climatic anomalies in aridity during the post-flight period of the previous year (t-1) of their adult stage. Panel **a** shows population abundance over time in relation to the population position within the species bioclimatic range (Supplementary Fig. 35). Divergent responses according to the position of the site in the species bioclimatic range are shown at 0.1 intervals from the leading (range position = -1) to the trailing margin (range position = 1), displayed from the leading to trailing (blue and red, respectively; white indicates centre). Panel **b** shows the boxplot of the local climatic anomalies over time for the species. Dashed line related to the lack of climatic anomalies.

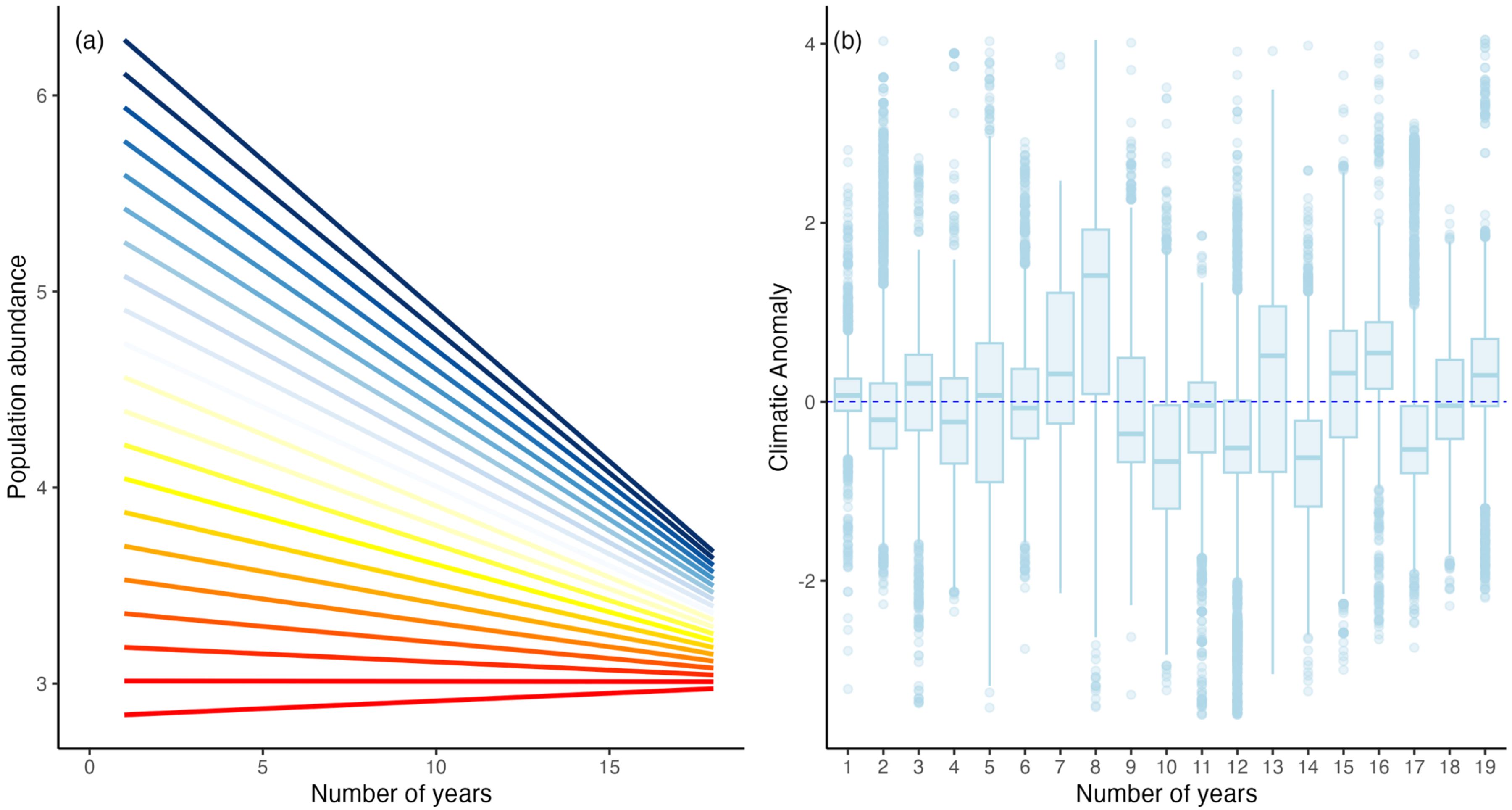

Supplementary Figure 47. Population abundance (log scale) and climatic anomalies over time for *Lysandra coridon*, a species best adapted to local climatic anomalies in temperature during the post-flight period of the previous year (t-1) of their adult stage. Panel **a** shows population abundance over time in relation to the population position within the species bioclimatic range (Supplementary Fig. 35). Divergent responses according to the position of the site in the species bioclimatic range are shown at 0.1 intervals from the leading (range position = -1) to the trailing margin (range position = 1), displayed from the leading to trailing (blue and red, respectively; white indicates centre). Panel **b** shows the boxplot of the local climatic anomalies over time for the species. Dashed line related to the lack of climatic anomalies.

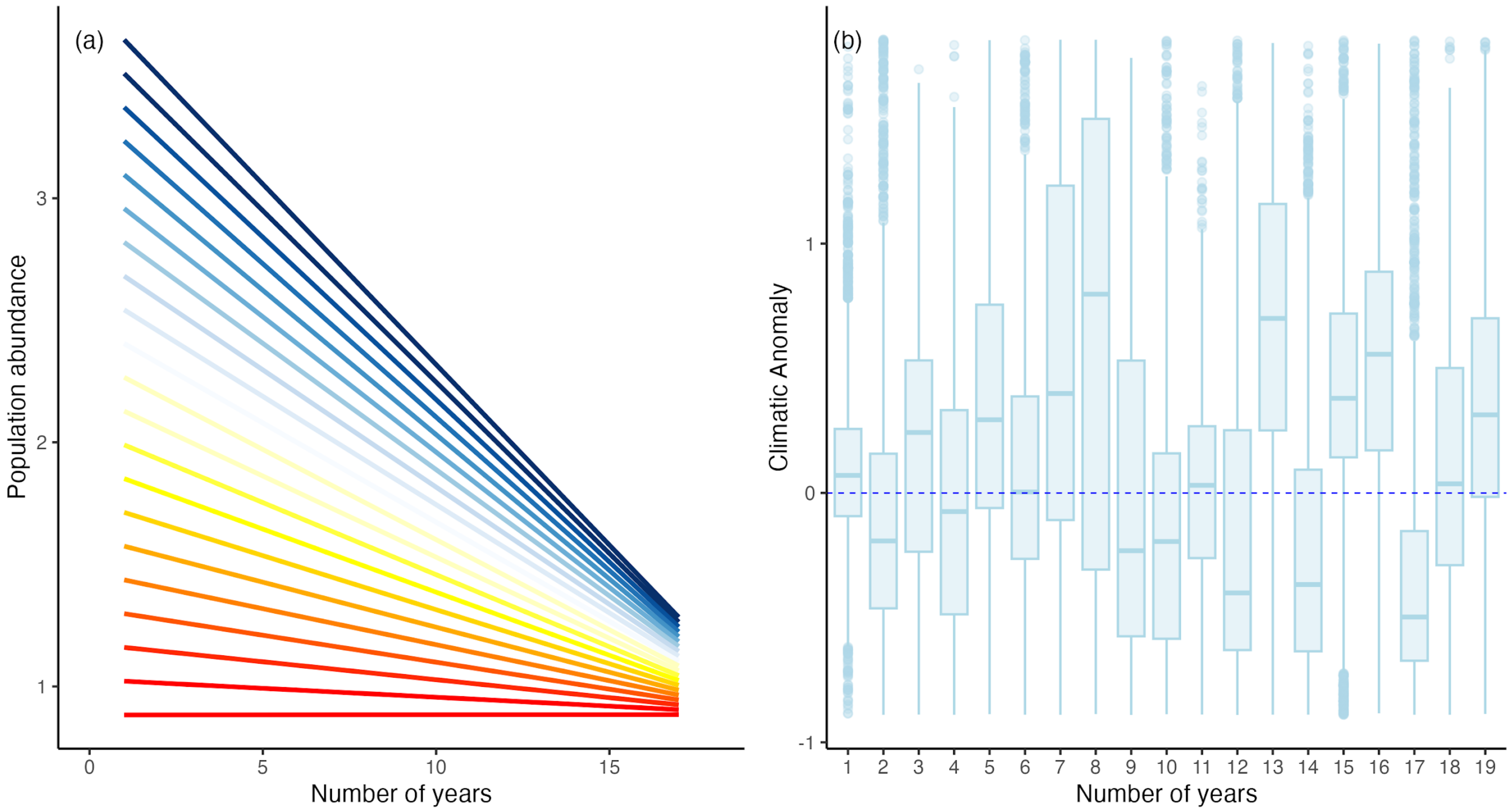

Supplementary Figure 48. Population abundance (log scale) and climatic anomalies over time for *Nymphalis polychloros*, a species best adapted to local climatic anomalies in precipitation during the pre-flight period of the previous year (t-1) of their adult stage. Panel **a** shows population abundance over time in relation to the population position within the species bioclimatic range (Supplementary Fig. 35). Divergent responses according to the position of the site in the species bioclimatic range are shown at 0.1 intervals from the leading (range position = -1) to the trailing margin (range position = 1), displayed from the leading to trailing (blue and red, respectively; white indicates centre). Panel **b** shows the boxplot of the local climatic anomalies over time for the species. Dashed line related to the lack of climatic anomalies.

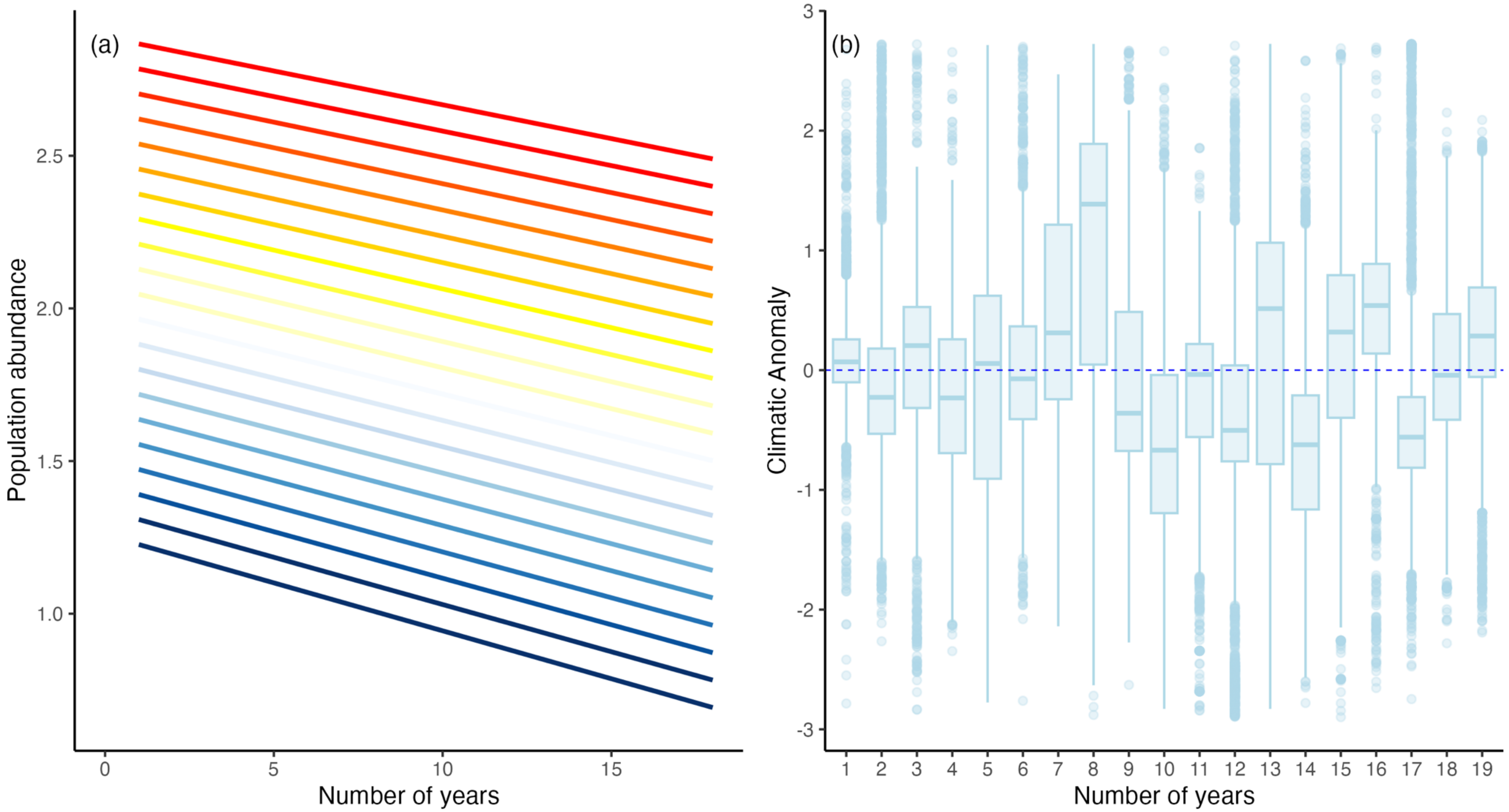

Supplementary Figure 49. Population abundance (log scale) and climatic anomalies over time for *Papilio machaon*, a species best adapted to local climatic anomalies in temperature during the flight period of the year (t) of their adult stage. Panel **a** shows population abundance over time in relation to the population position within the species bioclimatic range (Supplementary Fig. 35). Divergent responses according to the position of the site in the species bioclimatic range are shown at 0.1 intervals from the leading (range position = -1) to the trailing margin (range position = 1), displayed from the leading to trailing (blue and red, respectively; white indicates centre). Panel **b** shows the boxplot of the local climatic anomalies over time for the species. Dashed line related to the lack of climatic anomalies.

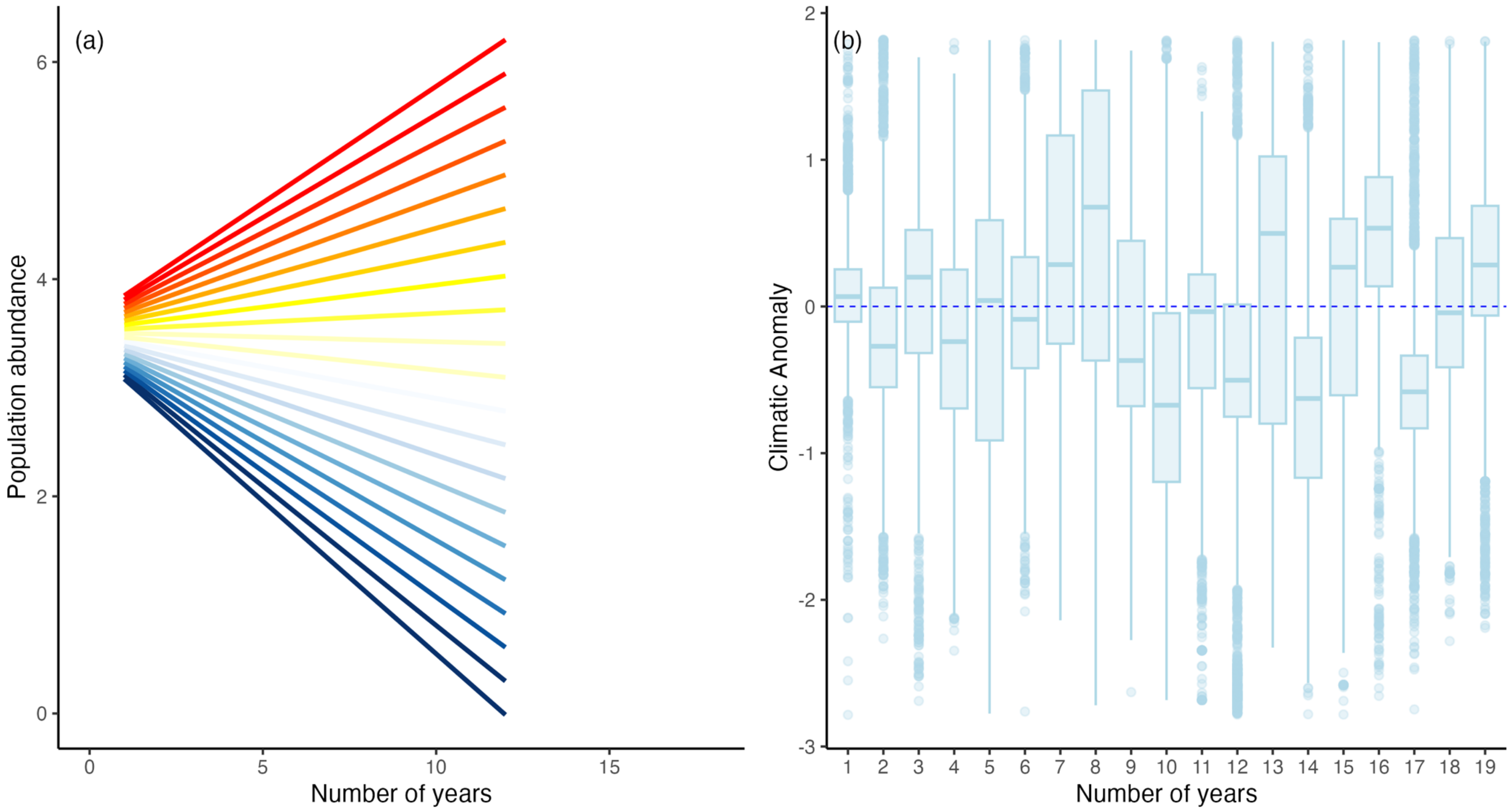

Supplementary Figure 50. Population abundance (log scale) and climatic anomalies over time for *Parnassius apollo*, a species best adapted to local climatic anomalies in precipitation during the pre-flight period of the previous year (t-1) of their adult stage. Panel **a** shows population abundance over time in relation to the population position within the species bioclimatic range (Supplementary Fig. 35). Divergent responses according to the position of the site in the species bioclimatic range are shown at 0.1 intervals from the leading (range position = -1) to the trailing margin (range position = 1), displayed from the leading to trailing (blue and red, respectively; white indicates centre). Panel **b** shows the boxplot of the local climatic anomalies over time for the species. Dashed line related to the lack of climatic anomalies.

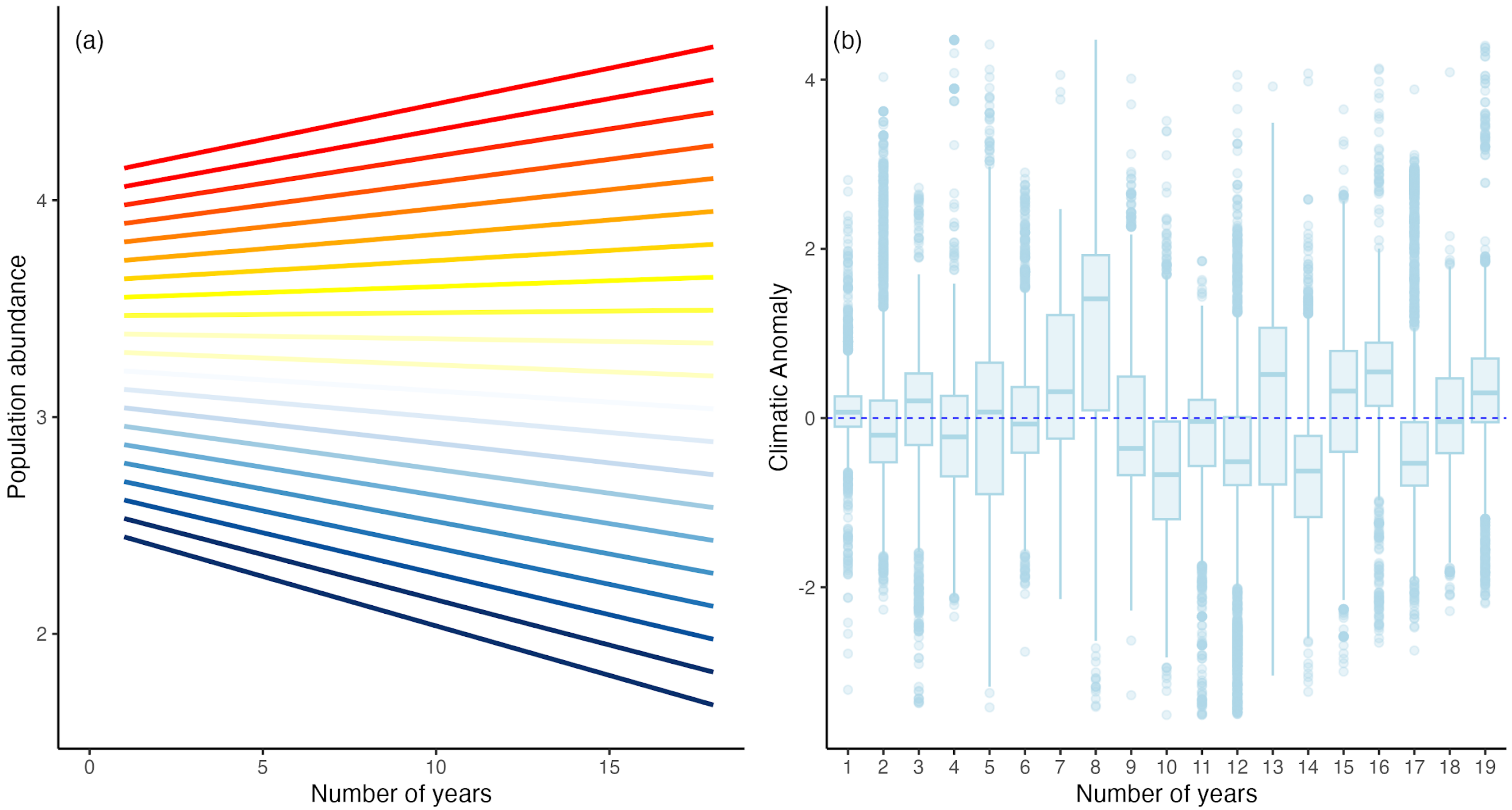

Supplementary Figure 51. Population abundance (log scale) and climatic anomalies over time for *Pieris brassicae*, a species best adapted to local climatic anomalies in temperature during the post-flight period of the previous year (t-1) of their adult stage. Panel **a** shows population abundance over time in relation to the population position within the species bioclimatic range (Supplementary Fig. 35). Divergent responses according to the position of the site in the species bioclimatic range are shown at 0.1 intervals from the leading (range position = -1) to the trailing margin (range position = 1), displayed from the leading to trailing (blue and red, respectively; white indicates centre). Panel **b** shows the boxplot of the local climatic anomalies over time for the species. Dashed line related to the lack of climatic anomalies.

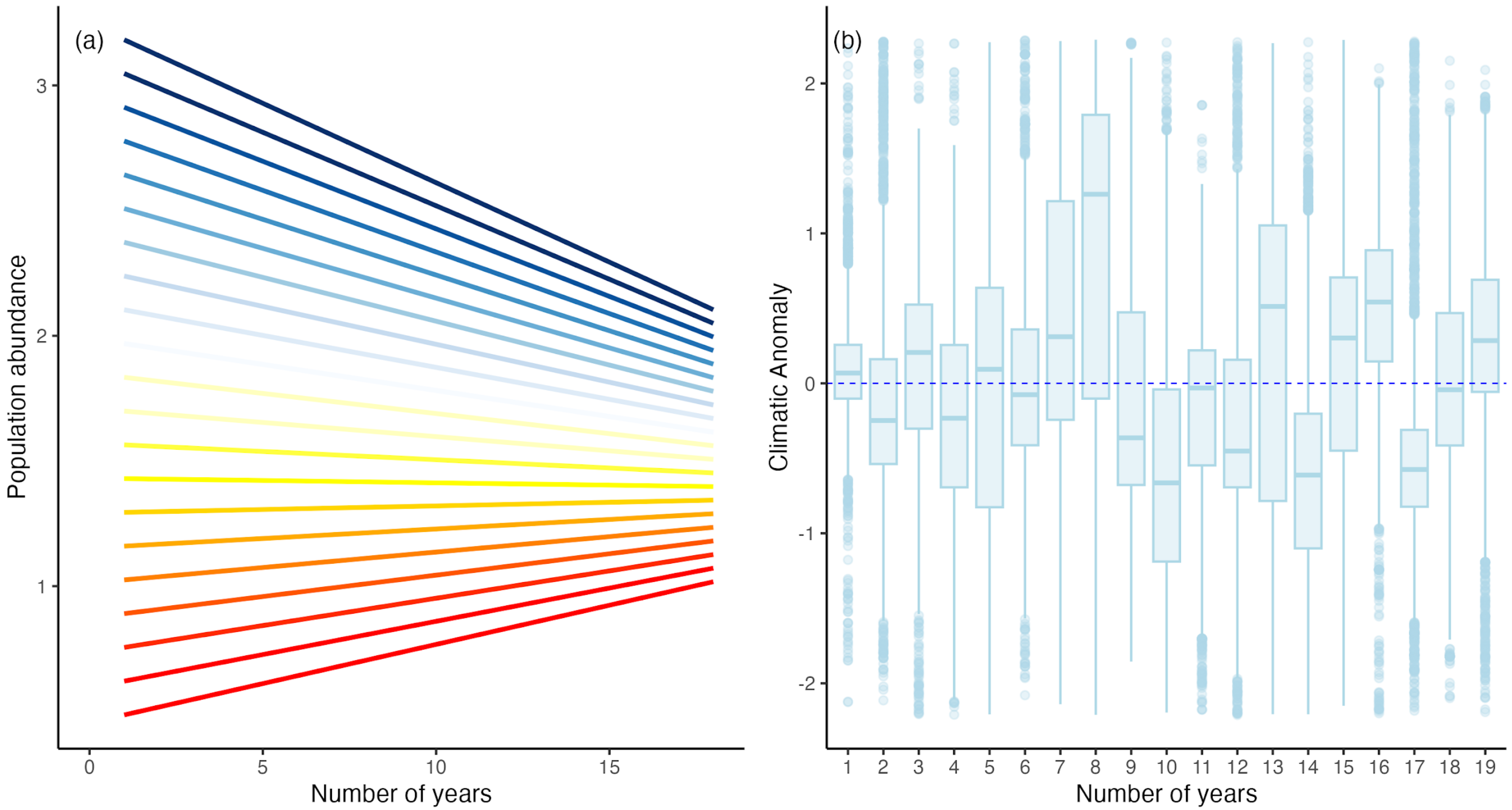

Supplementary Figure 52. Population abundance (log scale) and climatic anomalies over time for *Plebejus idas*, a species best adapted to local climatic anomalies in temperature during the post-flight period of the previous year (t-1) of their adult stage. Panel **a** shows population abundance over time in relation to the population position within the species bioclimatic range (Supplementary Fig.). Divergent responses according to the position of the site in the species bioclimatic range are shown at 0.1 intervals from the leading (range position = -1) to the trailing margin (range position = 1), displayed from the leading to trailing (blue and red, respectively; white indicates centre). Panel **b** shows the boxplot of the local climatic anomalies over time for the species. Dashed line related to the lack of climatic anomalies.

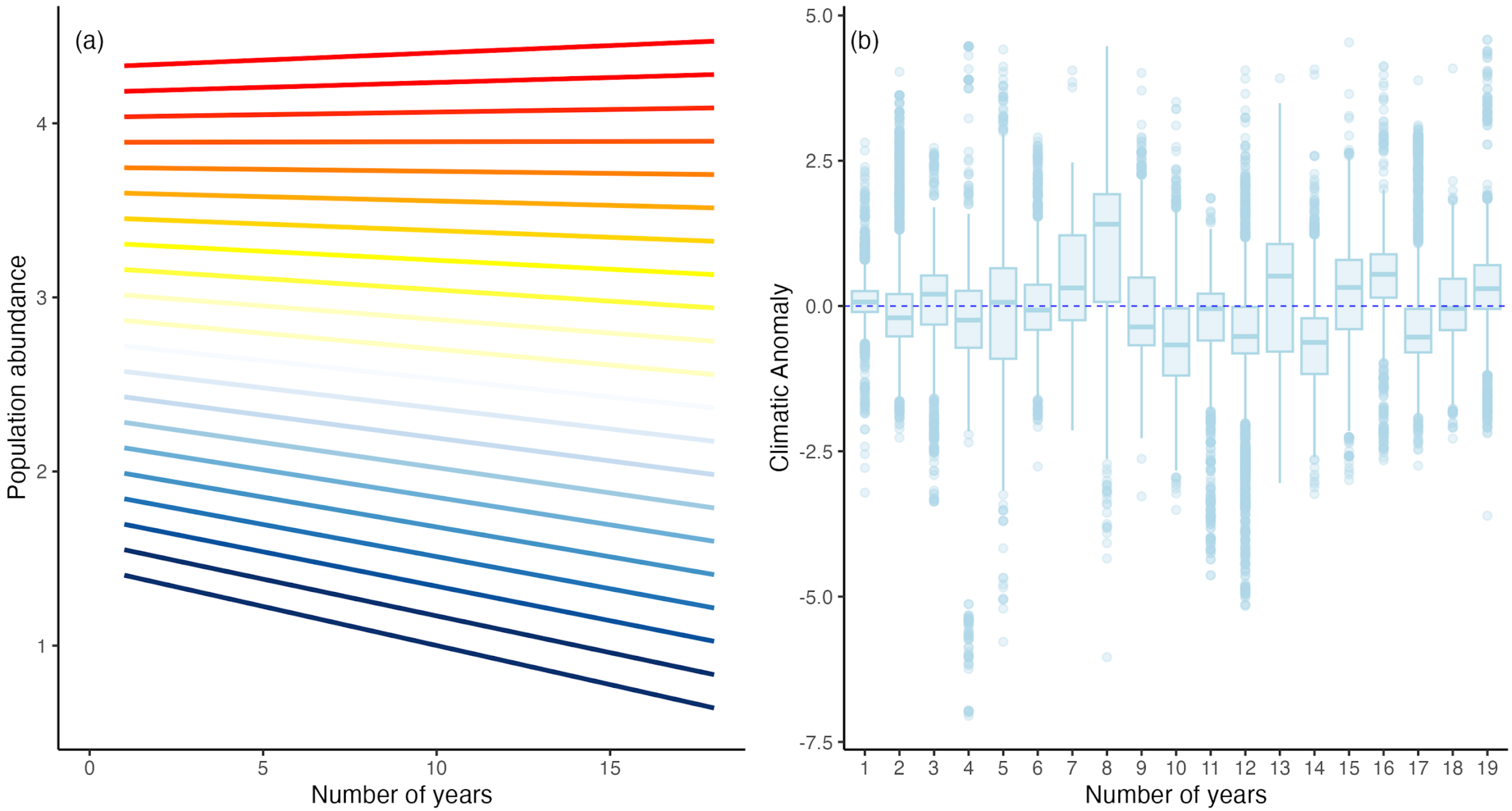

Supplementary Figure 53. Population abundance (log scale) and climatic anomalies over time for *Polyommatus icarus*, a species best adapted to local climatic anomalies in temperature during the OW period of the previous year (t-1) of their adult stage. Panel **a** shows population abundance over time in relation to the population position within the species bioclimatic range (Supplementary Fig.). Divergent responses according to the position of the site in the species bioclimatic range are shown at 0.1 intervals from the leading (range position = -1) to the trailing margin (range position = 1), displayed from the leading to trailing (blue and red, respectively; white indicates centre). Panel **b** shows the boxplot of the local climatic anomalies over time for the species. Dashed line related to the lack of climatic anomalies.

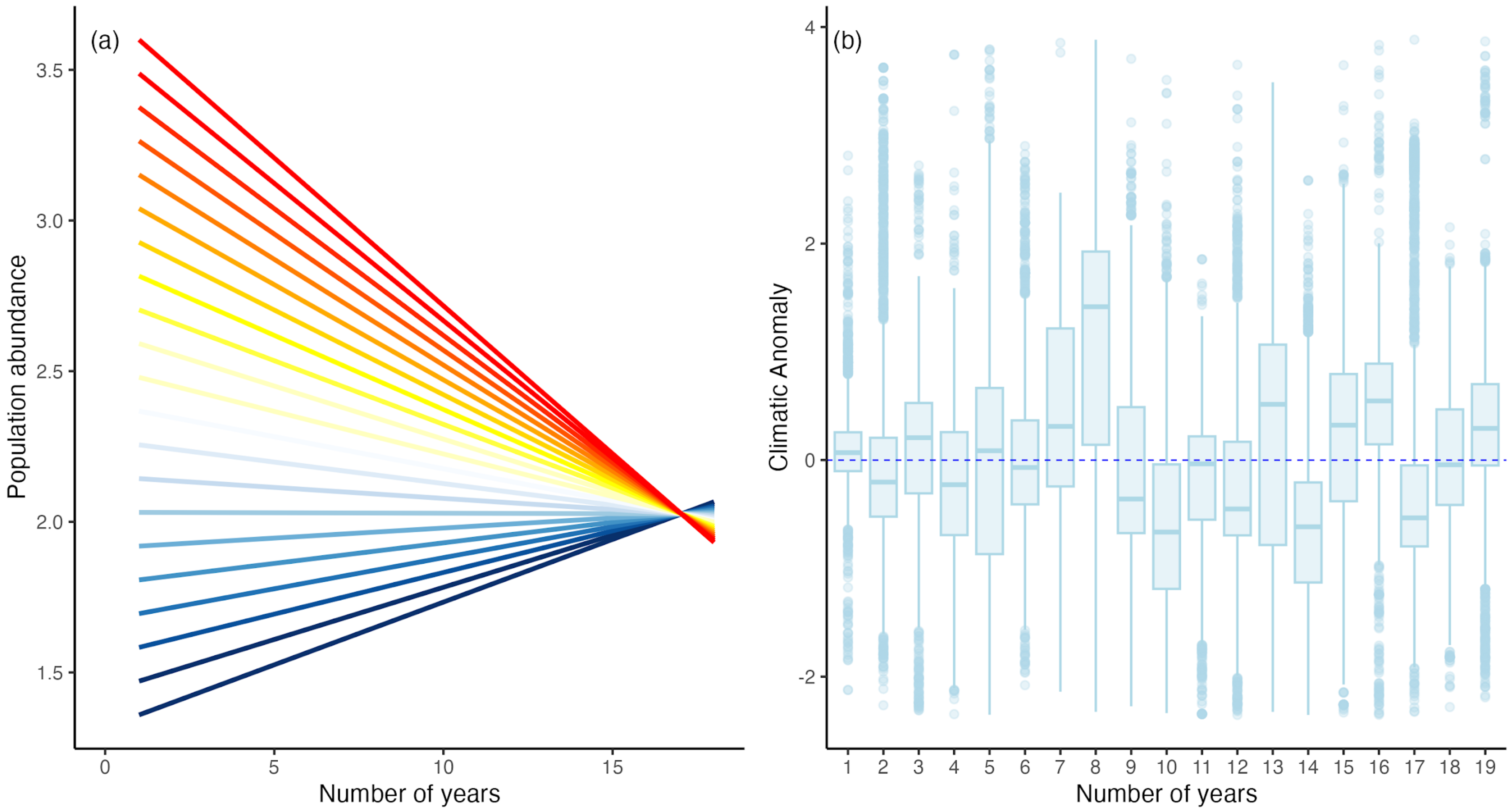

Supplementary Figure 54. Population abundance (log scale) and climatic anomalies over time for *Pseudophilotes panoptes*, a species best adapted to local climatic anomalies in aridity during the pre-flight period of the year (t) of their adult stage. Panel **a** shows population abundance over time in relation to the population position within the species bioclimatic range (Supplementary Fig. 35). Divergent responses according to the position of the site in the species bioclimatic range are shown at 0.1 intervals from the leading (range position = -1) to the trailing margin (range position = 1), displayed from the leading to trailing (blue and red, respectively; white indicates centre). Panel **b** shows the boxplot of the local climatic anomalies over time for the species. Dashed line related to the lack of climatic anomalies.

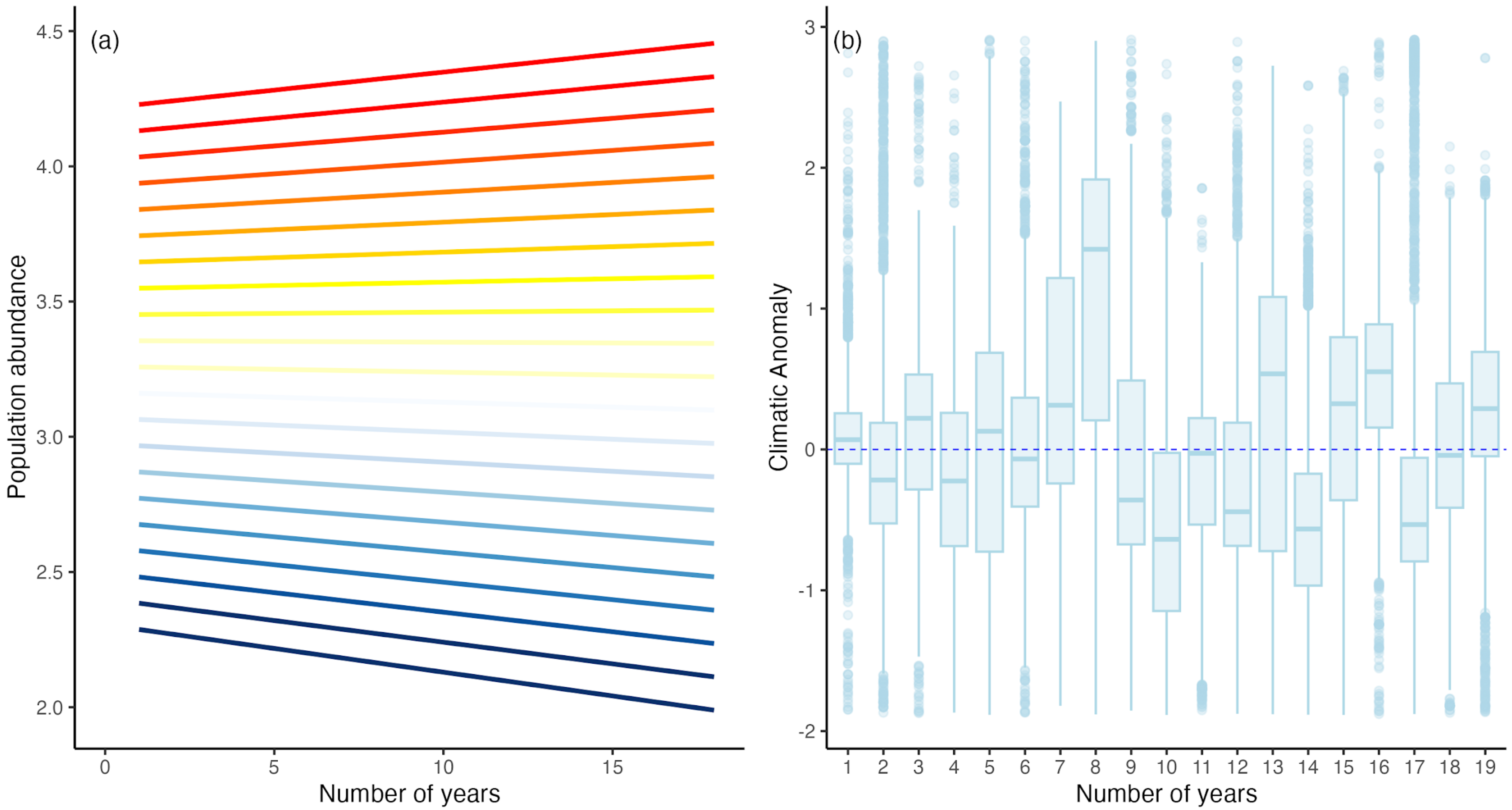

Supplementary Figure 55. Population abundance (log scale) and climatic anomalies over time for *Satyrium esculi,* a species best adapted to local climatic anomalies in temperature during the pre-flight period of the previous year (t-1) of their adult stage. Panel **a** shows population abundance over time in relation to the population position within the species bioclimatic range (Supplementary Fig. 35). Divergent responses according to the position of the site in the species bioclimatic range are shown at 0.1 intervals from the leading (range position = -1) to the trailing margin (range position = 1), displayed from the leading to trailing (blue and red, respectively; white indicates centre). Panel **b** shows the boxplot of the local climatic anomalies over time for the species. Dashed line related to the lack of climatic anomalies.

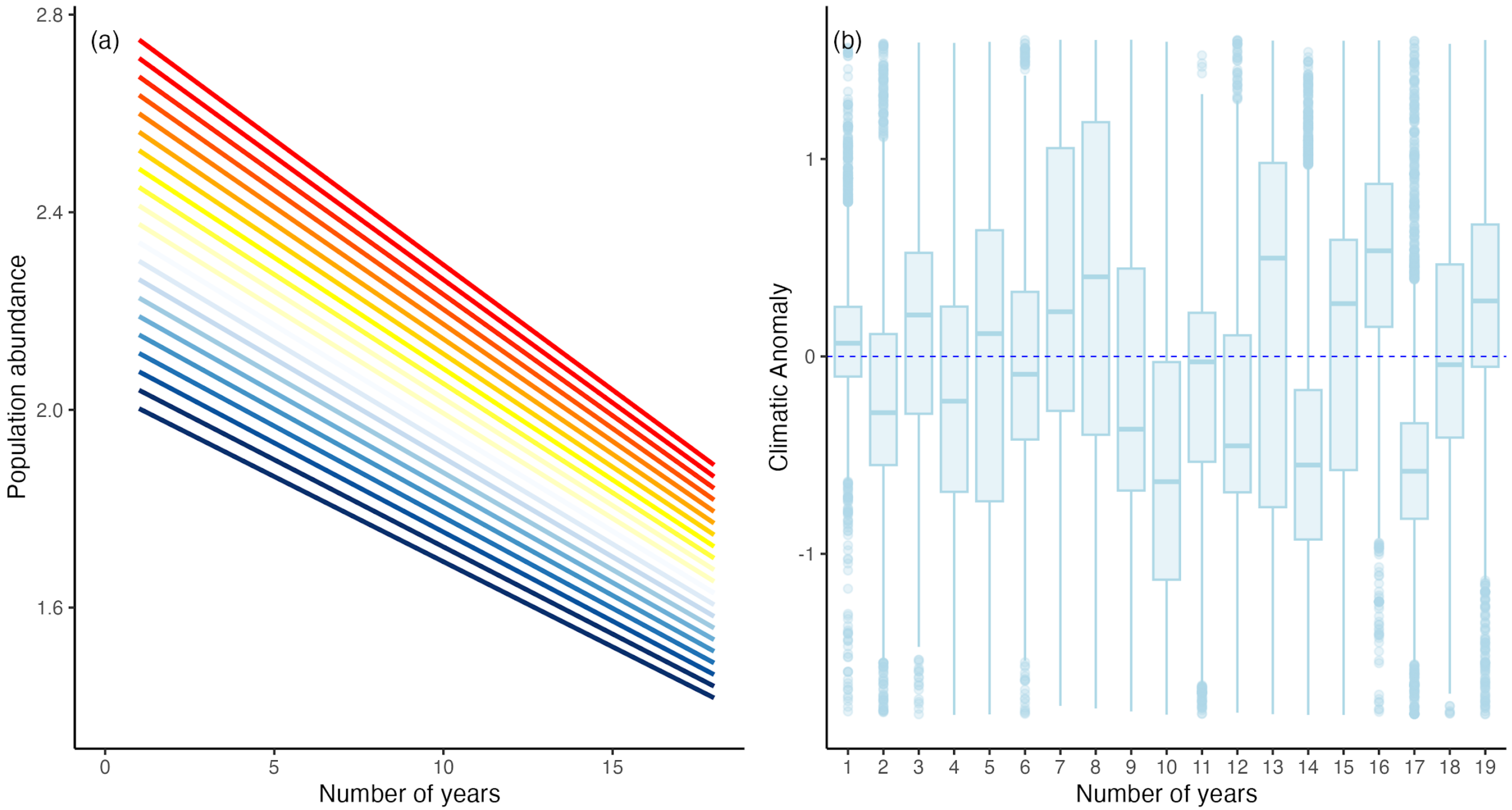

Supplementary Figure 56. Population abundance (log scale) and climatic anomalies over time for *Thymelicus acteon*, a species best adapted to local climatic anomalies in temperature during the pre-flight period of the previous year (t-1) of their adult stage. Panel **a** shows population abundance over time in relation to the population position within the species bioclimatic range (Supplementary Fig. 35). Divergent responses according to the position of the site in the species bioclimatic range are shown at 0.1 intervals from the leading (range position = -1) to the trailing margin (range position = 1), displayed from the leading to trailing (blue and red, respectively; white indicates centre). Panel **b** shows the boxplot of the local climatic anomalies over time for the species. Dashed line related to the lack of climatic anomalies.

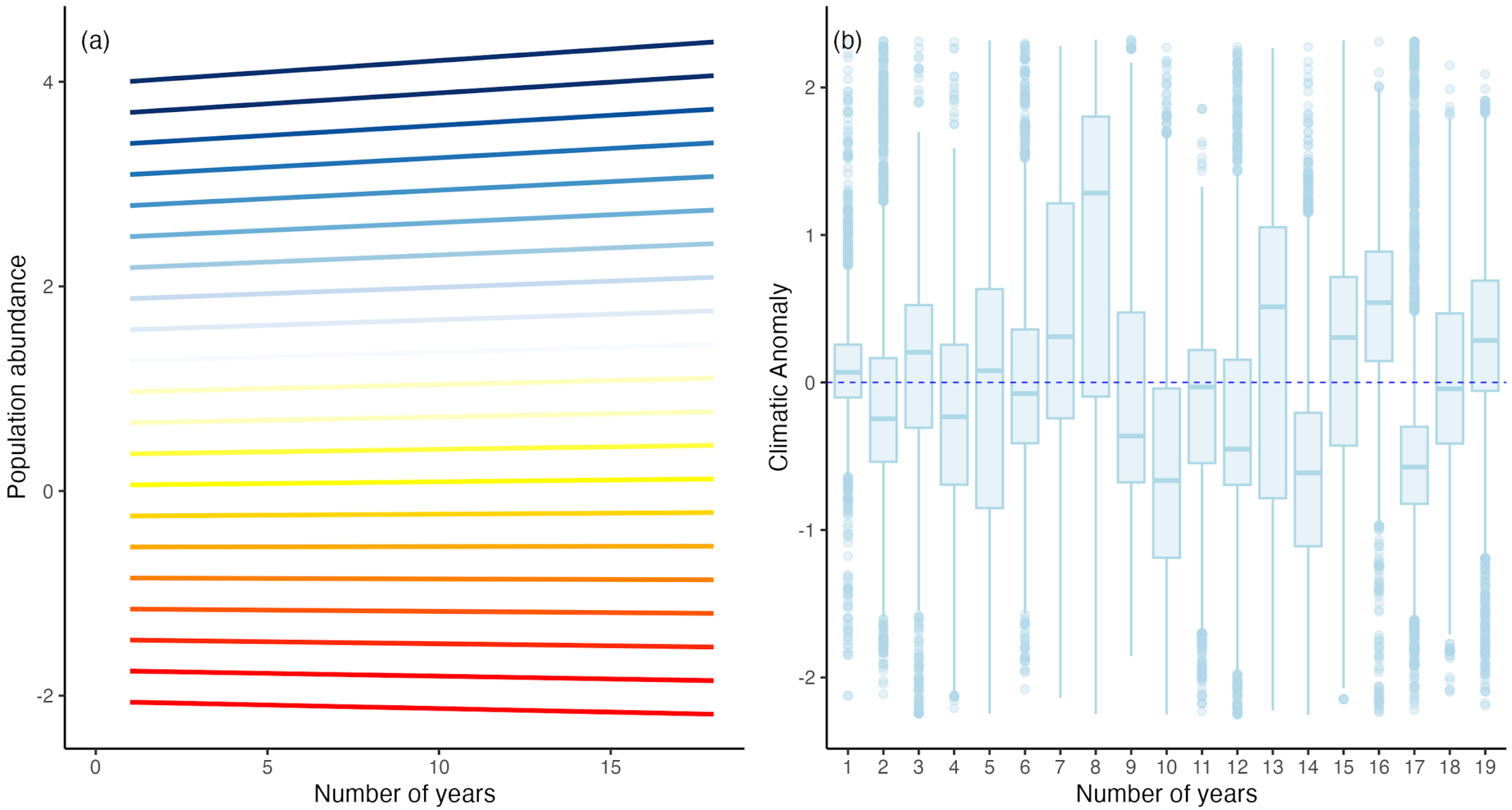

Supplementary Figure 57. Population abundance (log scale) and climatic anomalies over time for *Thymelicus lineola*, a species best adapted to local climatic anomalies in temperature during the post-flight period of the previous year (t-1) of their adult stage. Panel **a** shows population abundance over time in relation to the population position within the species bioclimatic range (Supplementary Fig. 35). Divergent responses according to the position of the site in the species bioclimatic range are shown at 0.1 intervals from the leading (range position = -1) to the trailing margin (range position = 1), displayed from the leading to trailing (blue and red, respectively; white indicates centre). Panel **b** shows the boxplot of the local climatic anomalies over time for the species. Dashed line related to the lack of climatic anomalies.

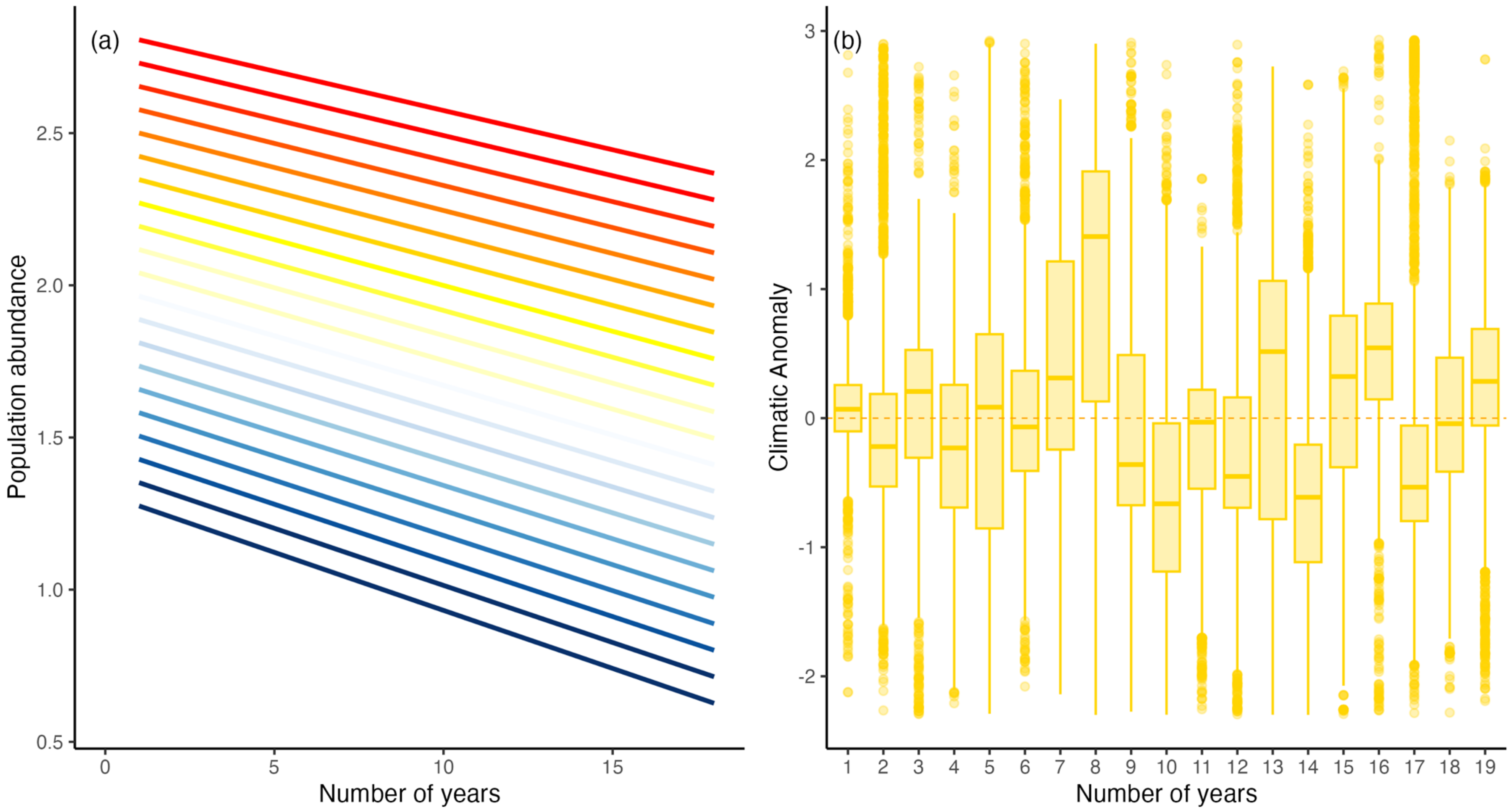

Supplementary Figure 58. Population abundance (log scale) and climatic anomalies over time for *Aricia artaxerxes*, a species best adapted to global climatic anomalies in temperature during the pre-flight period of the year (t) of their adult stage. Panel **a** shows population abundance over time in relation to the population position within the species bioclimatic range (Supplementary Fig. 35). Divergent responses according to the position of the site in the species bioclimatic range are shown at 0.1 intervals from the leading (range position = -1) to the trailing margin (range position = 1), displayed from the leading to trailing (blue and red, respectively; white indicates centre). Panel **b** shows the boxplot of the local climatic anomalies over time for the species. Dashed line related to the lack of climatic anomalies.

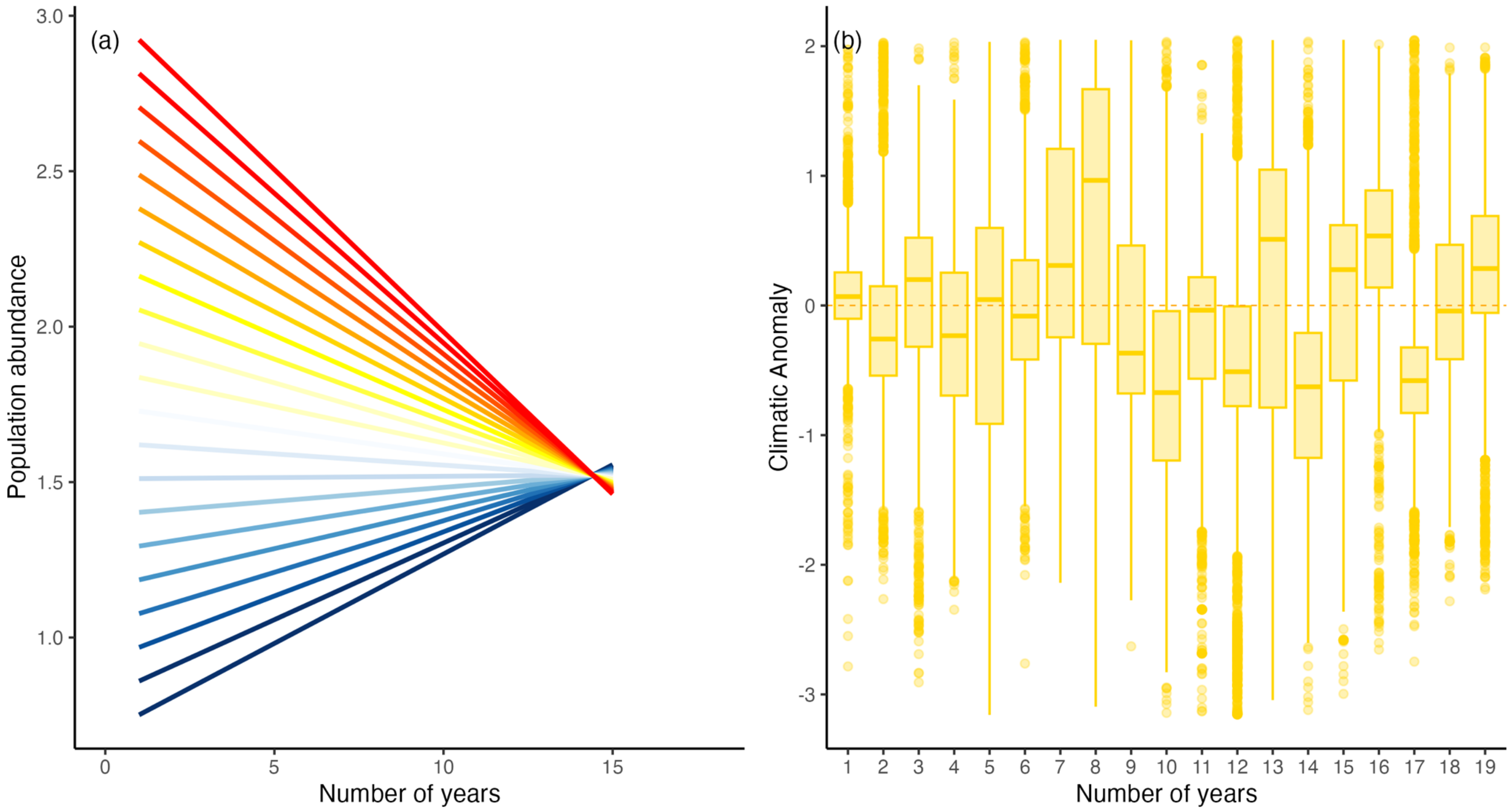

Supplementary Figure 59. Population abundance (log scale) and climatic anomalies over time for *Cupido osiris*, a species best adapted to global climatic anomalies in aridity during the pre-flight period of the year (t) of their adult stage. Panel **a** shows population abundance over time in relation to the population position within the species bioclimatic range (Supplementary Fig. 35). Divergent responses according to the position of the site in the species bioclimatic range are shown at 0.1 intervals from the leading (range position = -1) to the trailing margin (range position = 1), displayed from the leading to trailing (blue and red, respectively; white indicates centre). Panel **b** shows the boxplot of the local climatic anomalies over time for the species. Dashed line related to the lack of climatic anomalies.

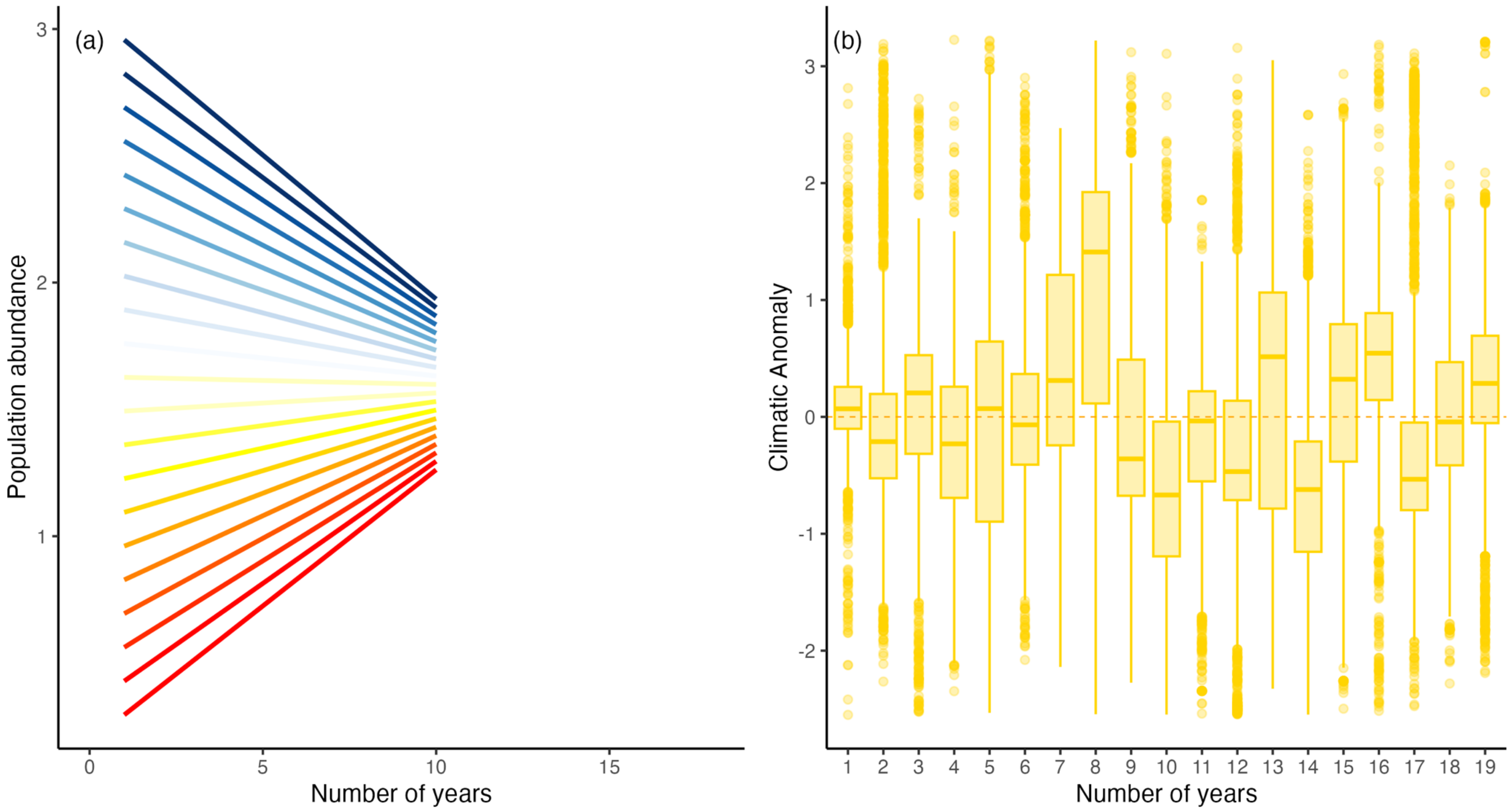

Supplementary Figure 60. Population abundance (log scale) and climatic anomalies over time for *Laeosopis roboris*, a species best adapted to global climatic anomalies in temperature during the flight period of the year (t) of their adult stage. Panel **a** shows population abundance over time in relation to the population position within the species bioclimatic range (Supplementary Fig. 35). Divergent responses according to the position of the site in the species bioclimatic range are shown at 0.1 intervals from the leading (range position = -1) to the trailing margin (range position = 1), displayed from the leading to trailing (blue and red, respectively; white indicates centre). Panel **b** shows the boxplot of the local climatic anomalies over time for the species. Dashed line related to the lack of climatic anomalies.

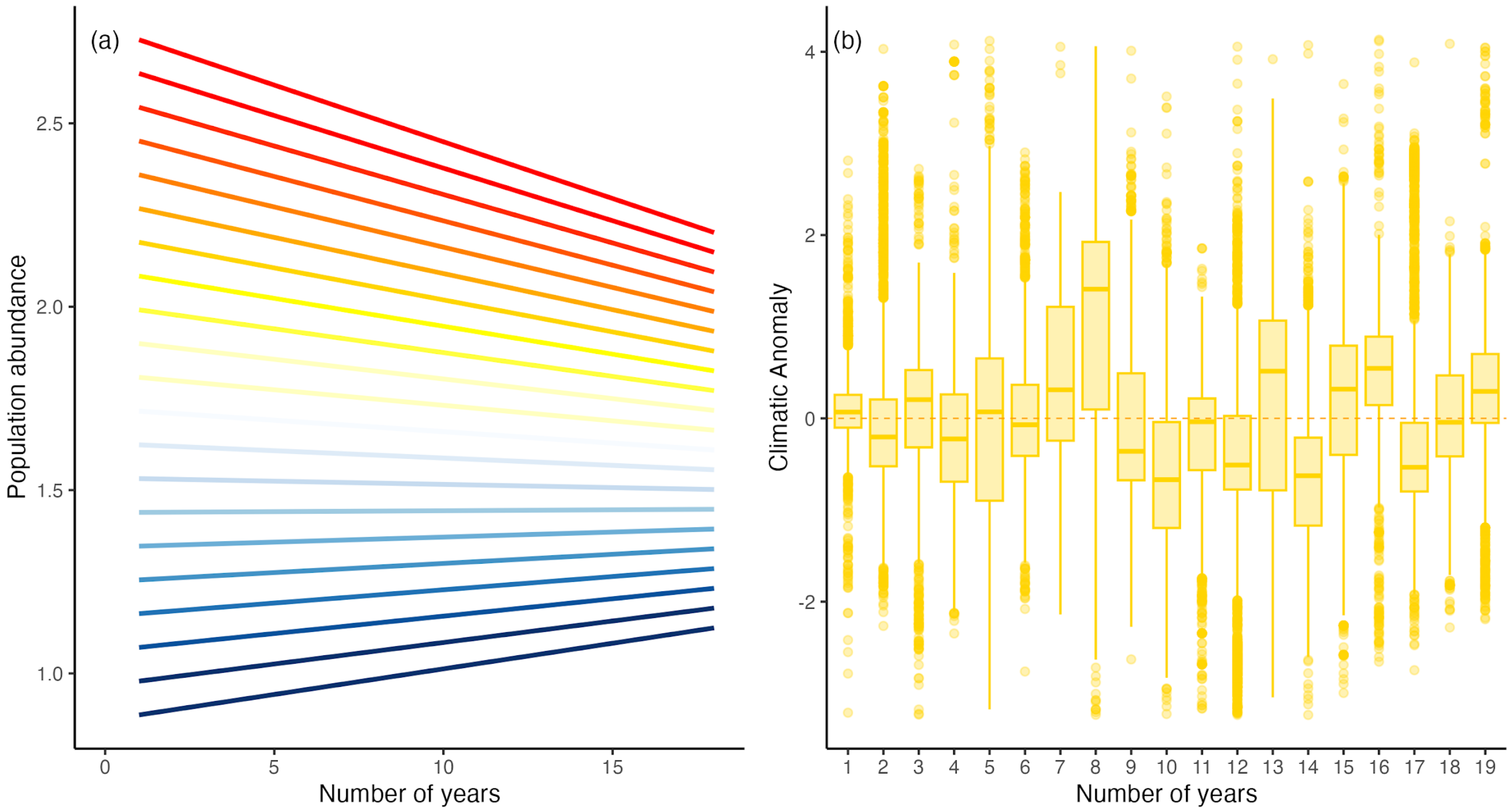

Supplementary Figure 61. Population abundance (log scale) and climatic anomalies over time for *Lycaena phlaeas*, a species best adapted to global climatic anomalies in temperature during the pre-flight period of the previous year (t-1) of their adult stage. Panel **a** shows population abundance over time in relation to the population position within the species bioclimatic range (Supplementary Fig. 35). Divergent responses according to the position of the site in the species bioclimatic range are shown at 0.1 intervals from the leading (range position = -1) to the trailing margin (range position = 1), displayed from the leading to trailing (blue and red, respectively; white indicates centre). Panel **b** shows the boxplot of the local climatic anomalies over time for the species. Dashed line related to the lack of climatic anomalies.

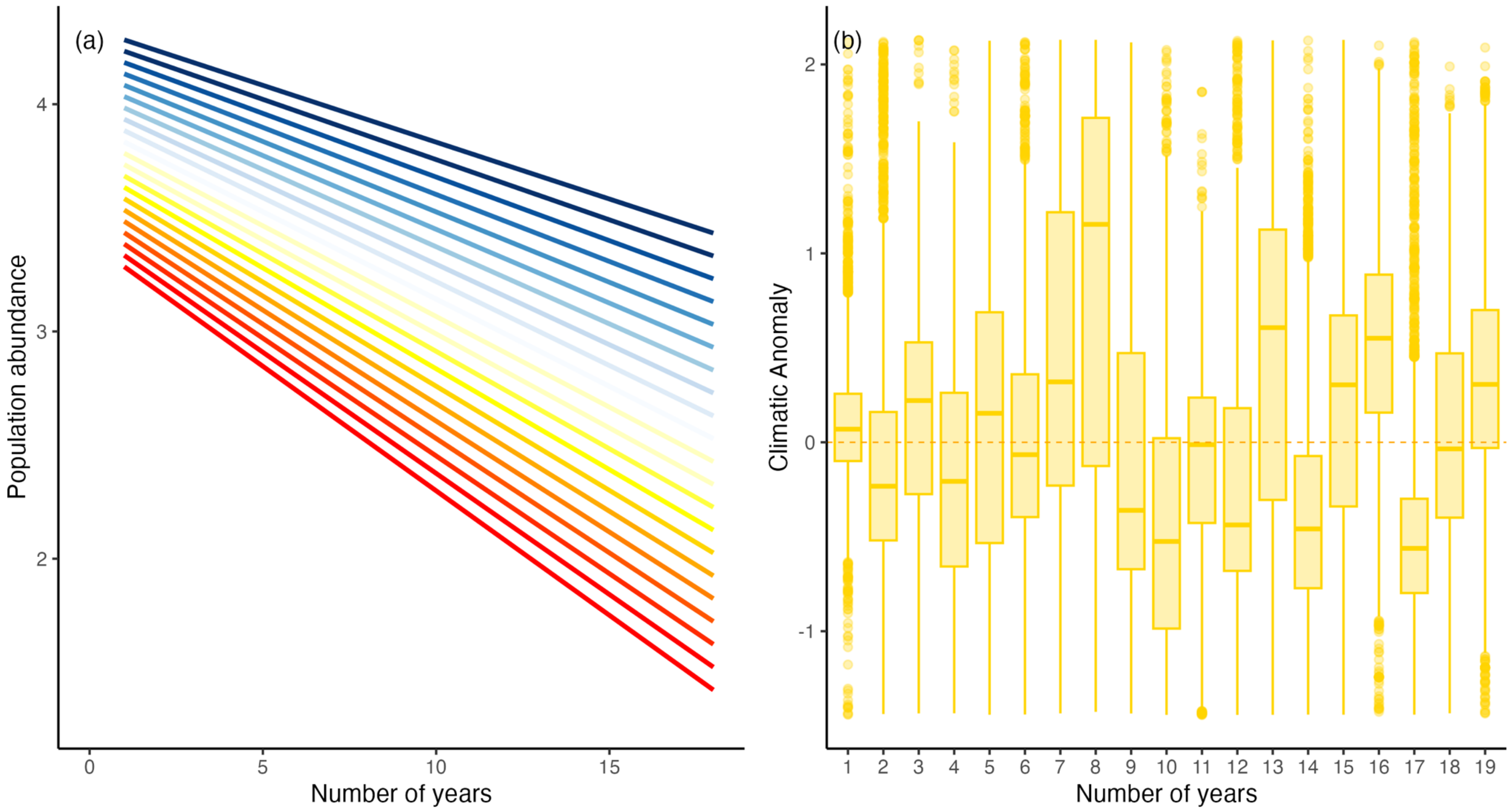

Supplementary Figure 62. Population abundance (log scale) and climatic anomalies over time for *Lycaena virgaureae*, a species best adapted to global climatic anomalies in precipitation during the flight period of the year (t) of their adult stage. Panel **a** shows population abundance over time in relation to the population position within the species bioclimatic range (Supplementary Fig. 35). Divergent responses according to the position of the site in the species bioclimatic range are shown at 0.1 intervals from the leading (range position = -1) to the trailing margin (range position = 1), displayed from the leading to trailing (blue and red, respectively; white indicates centre). Panel **b** shows the boxplot of the local climatic anomalies over time for the species. Dashed line related to the lack of climatic anomalies.

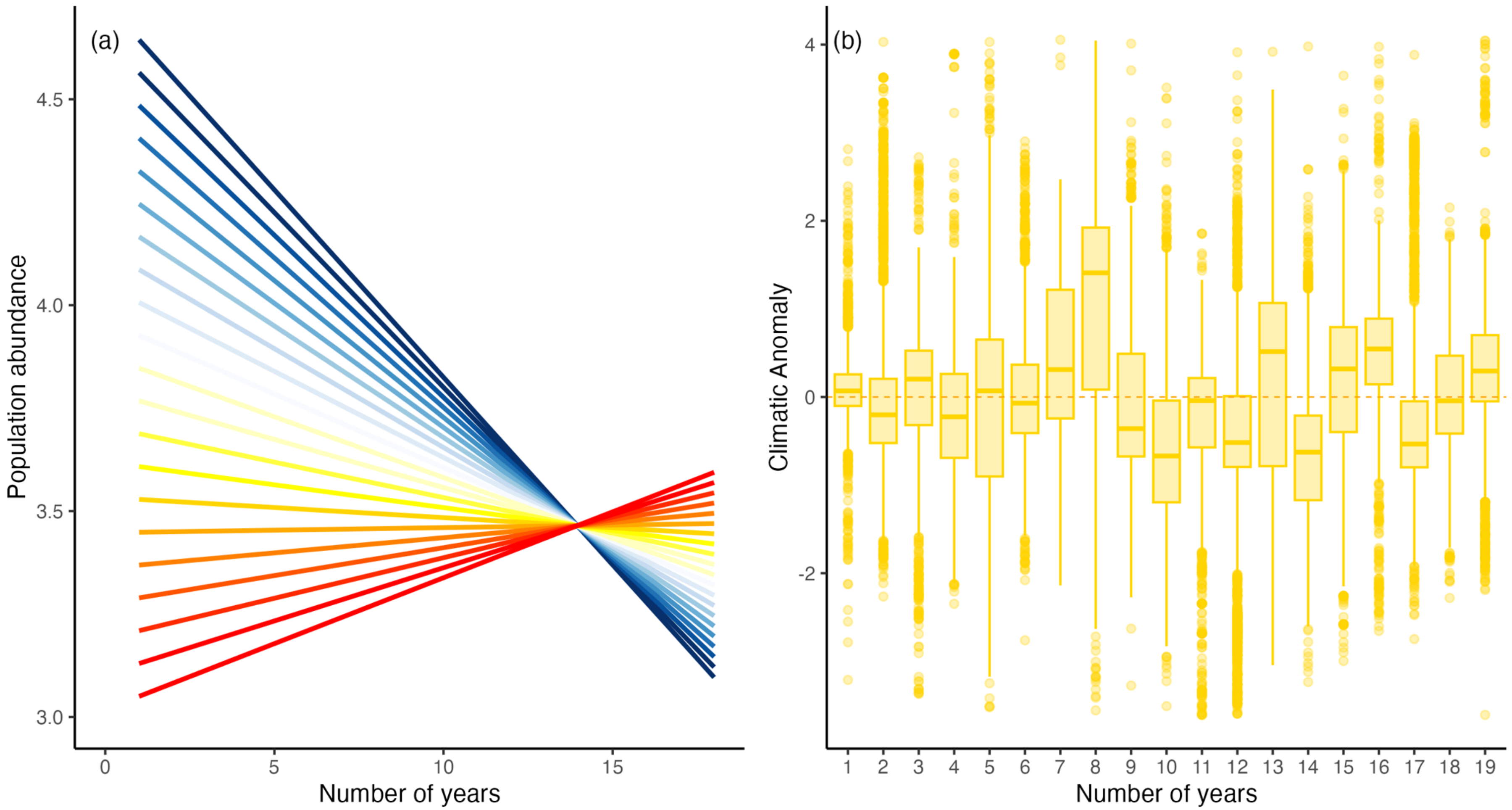

Supplementary Figure 63. Population abundance (log scale) and climatic anomalies over time for *Lysandra hispana*, a species best adapted to global climatic anomalies in aridity during the OW period of the year (t) of their adult stage. Panel **a** shows population abundance over time in relation to the population position within the species bioclimatic range (Supplementary Fig. 35). Divergent responses according to the position of the site in the species bioclimatic range are shown at 0.1 intervals from the leading (range position = -1) to the trailing margin (range position = 1), displayed from the leading to trailing (blue and red, respectively; white indicates centre). Panel **b** shows the boxplot of the local climatic anomalies over time for the species. Dashed line related to the lack of climatic anomalies.

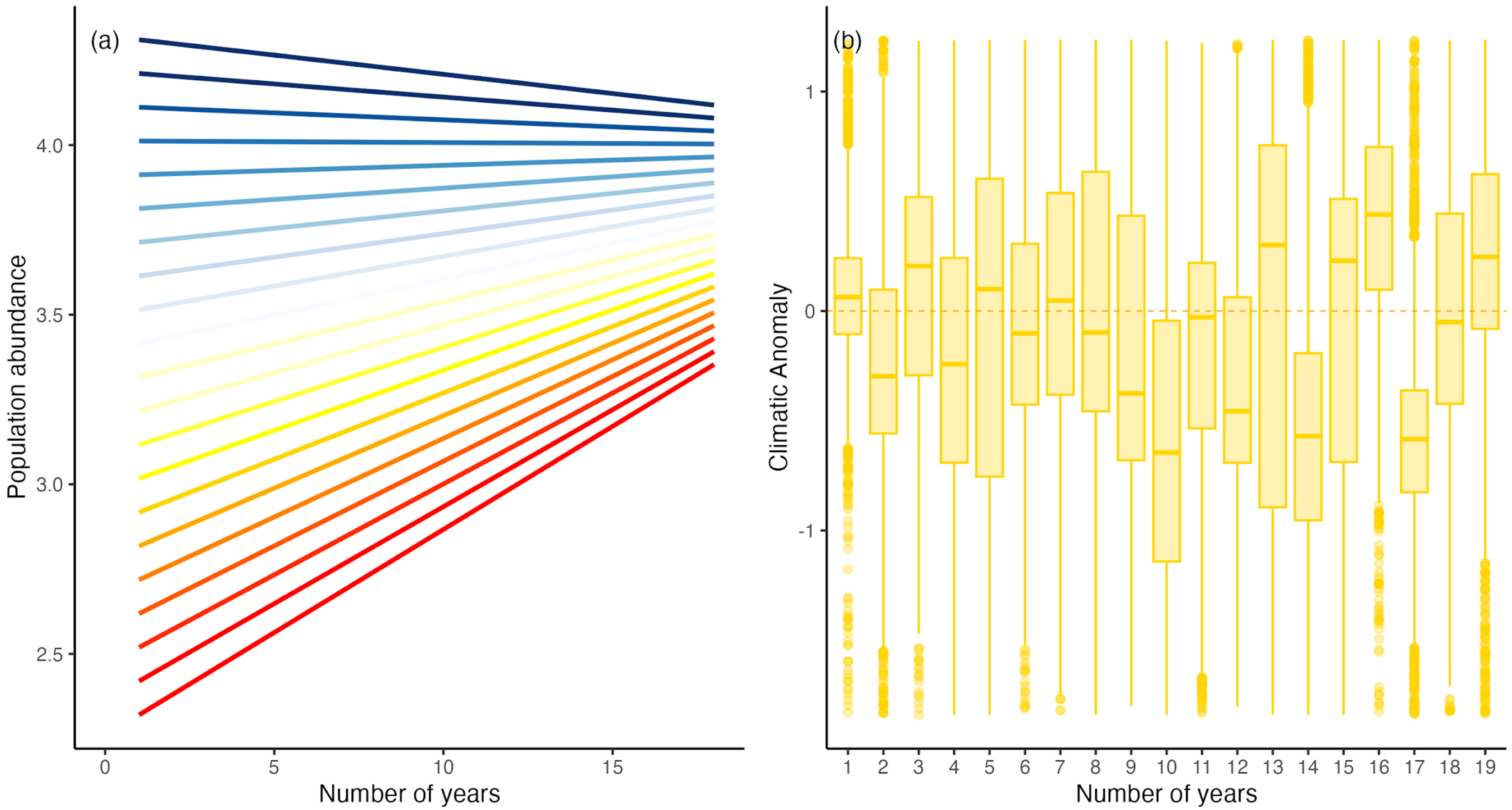

Supplementary Figure 64. Population abundance (log scale) and climatic anomalies over time for *Melanargia galathea*, a species best adapted to global climatic anomalies in temperature during the pre-flight period of the year (t) of their adult stage. Panel **a** shows population abundance over time in relation to the population position within the species bioclimatic range (Supplementary 74). Divergent responses according to the position of the site in the species bioclimatic range are shown at 0.1 intervals from the leading (range position = -1) to the trailing margin (range position = 1), displayed from the leading to trailing (blue and red, respectively; white indicates centre). Panel **b** shows the boxplot of the local climatic anomalies over time for the species. Dashed line related to the lack of climatic anomalies.

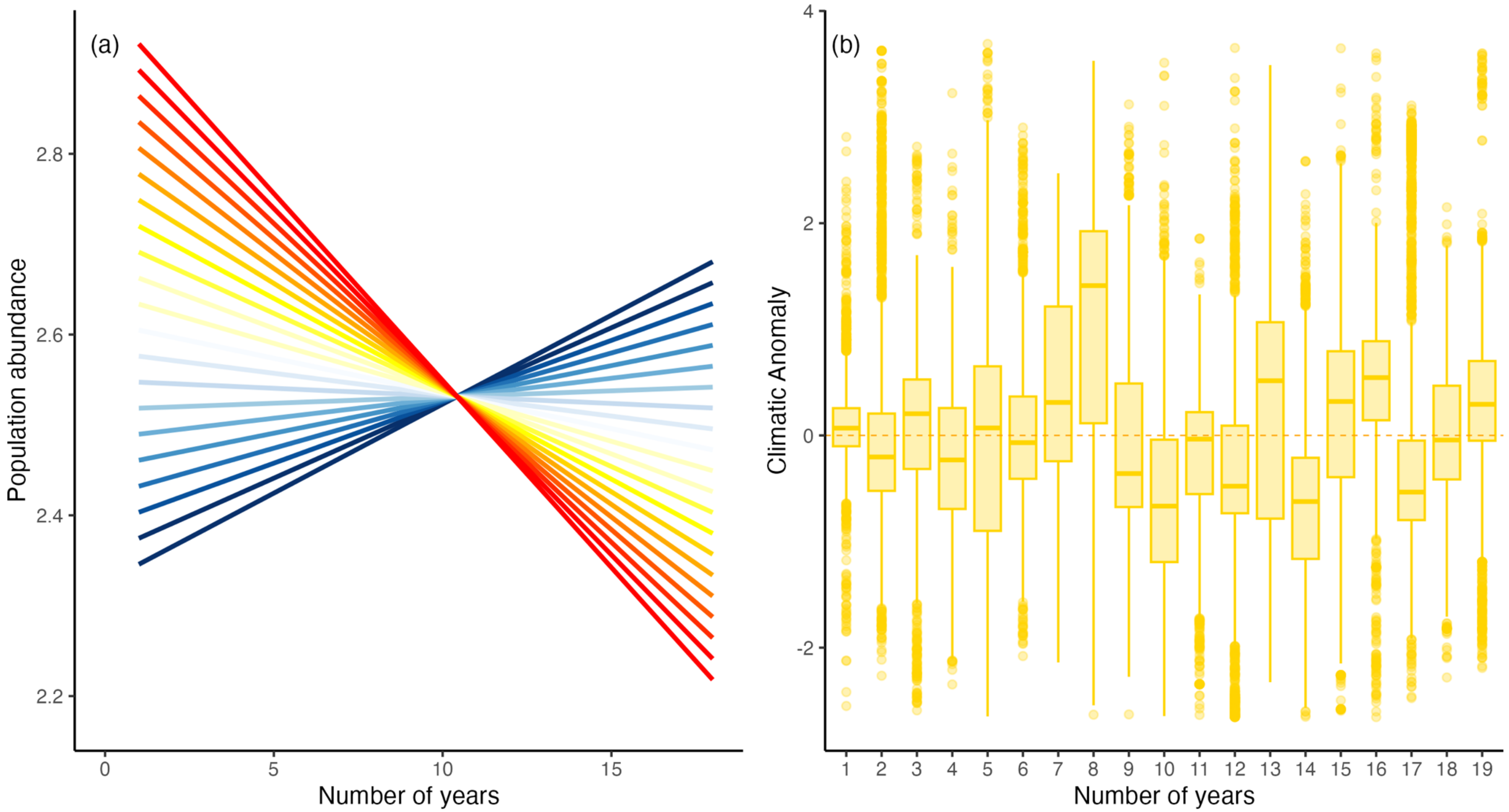

Supplementary Figure 65. Population abundance (log scale) and climatic anomalies over time for *Ochlodes sylvanus*, a species best adapted to global climatic anomalies in temperature during the flight period of the previous year (t-1) of their adult stage. Panel **a** shows population abundance over time in relation to the population position within the species bioclimatic range (Supplementary Fig. 35). Divergent responses according to the position of the site in the species bioclimatic range are shown at 0.1 intervals from the leading (range position = -1) to the trailing margin (range position = 1), displayed from the leading to trailing (blue and red, respectively; white indicates centre). Panel **b** shows the boxplot of the local climatic anomalies over time for the species. Dashed line related to the lack of climatic anomalies.

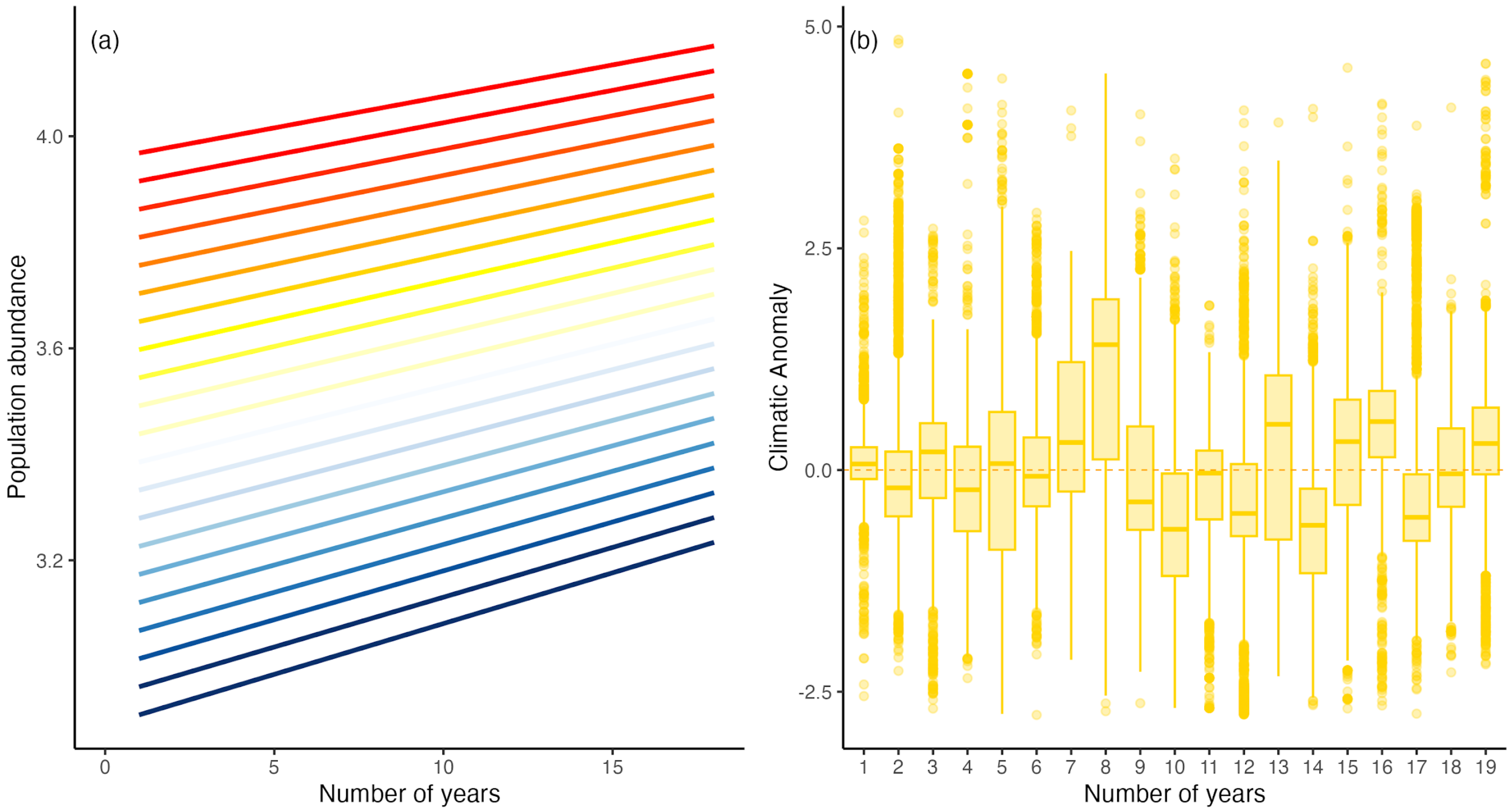

Supplementary Figure 66. Population abundance (log scale) and climatic anomalies over time for *Pararge aegeria*, a species best adapted to global climatic anomalies in precipitation during the post-flight period of the previous year (t-1) of their adult stage. Panel **a** shows population abundance over time in relation to the population position within the species bioclimatic range (Supplementary Fig. 35). Divergent responses according to the position of the site in the species bioclimatic range are shown at 0.1 intervals from the leading (range position = -1) to the trailing margin (range position = 1), displayed from the leading to trailing (blue and red, respectively; white indicates centre). Panel **b** shows the boxplot of the local climatic anomalies over time for the species. Dashed line related to the lack of climatic anomalies.

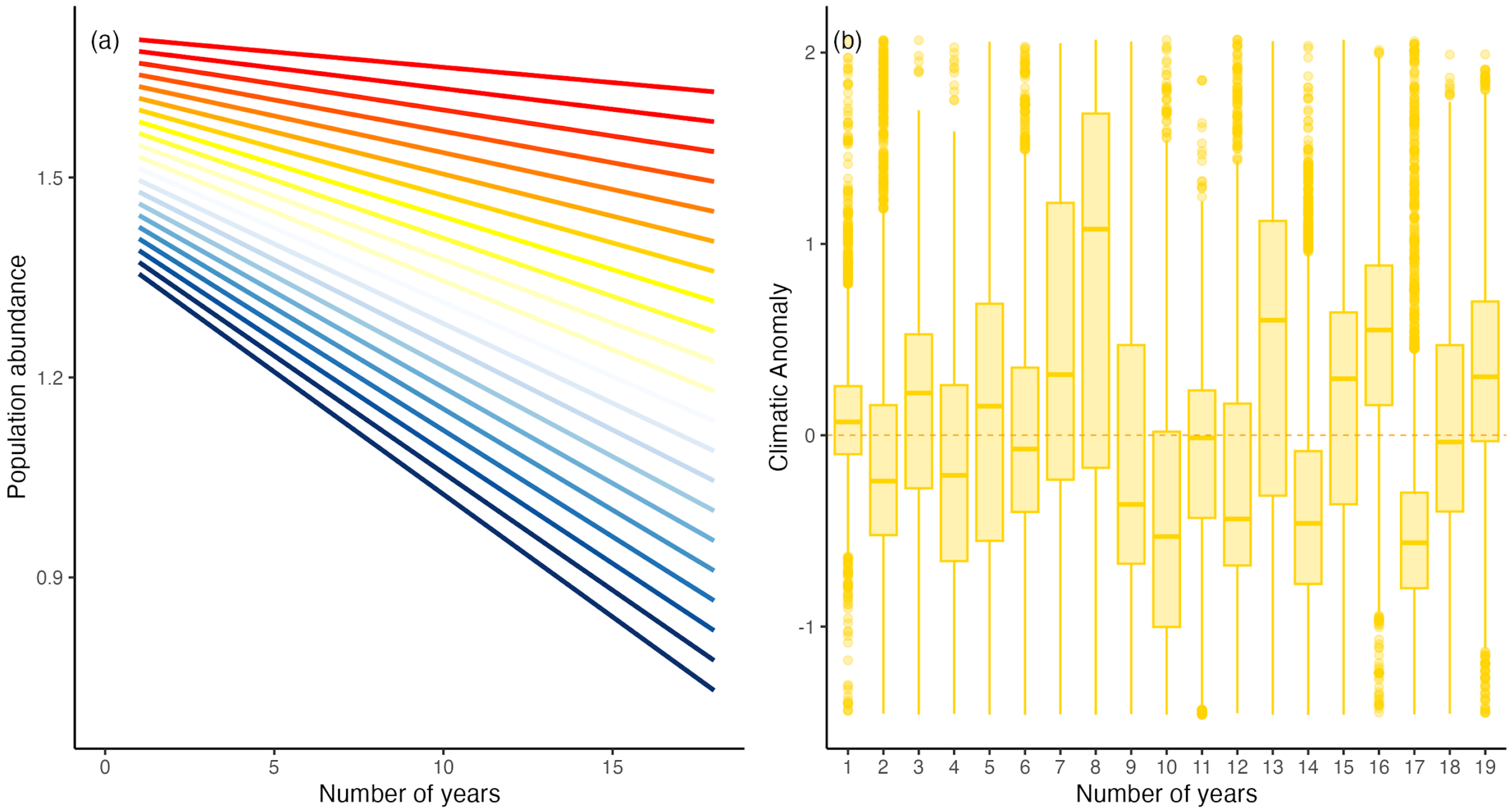

Supplementary Figure 67. Population abundance (log scale) and climatic anomalies over time for *Pyrgus malvae*, a species best adapted to global climatic anomalies in temperature during the post-flight period of the previous year (t-1) of their adult stage. Panel **a** shows population abundance over time in relation to the population position within the species bioclimatic range (Supplementary Fig. 35). Divergent responses according to the position of the site in the species bioclimatic range are shown at 0.1 intervals from the leading (range position = -1) to the trailing margin (range position = 1), displayed from the leading to trailing (blue and red, respectively; white indicates centre). Panel **b** shows the boxplot of the local climatic anomalies over time for the species. Dashed line related to the lack of climatic anomalies.

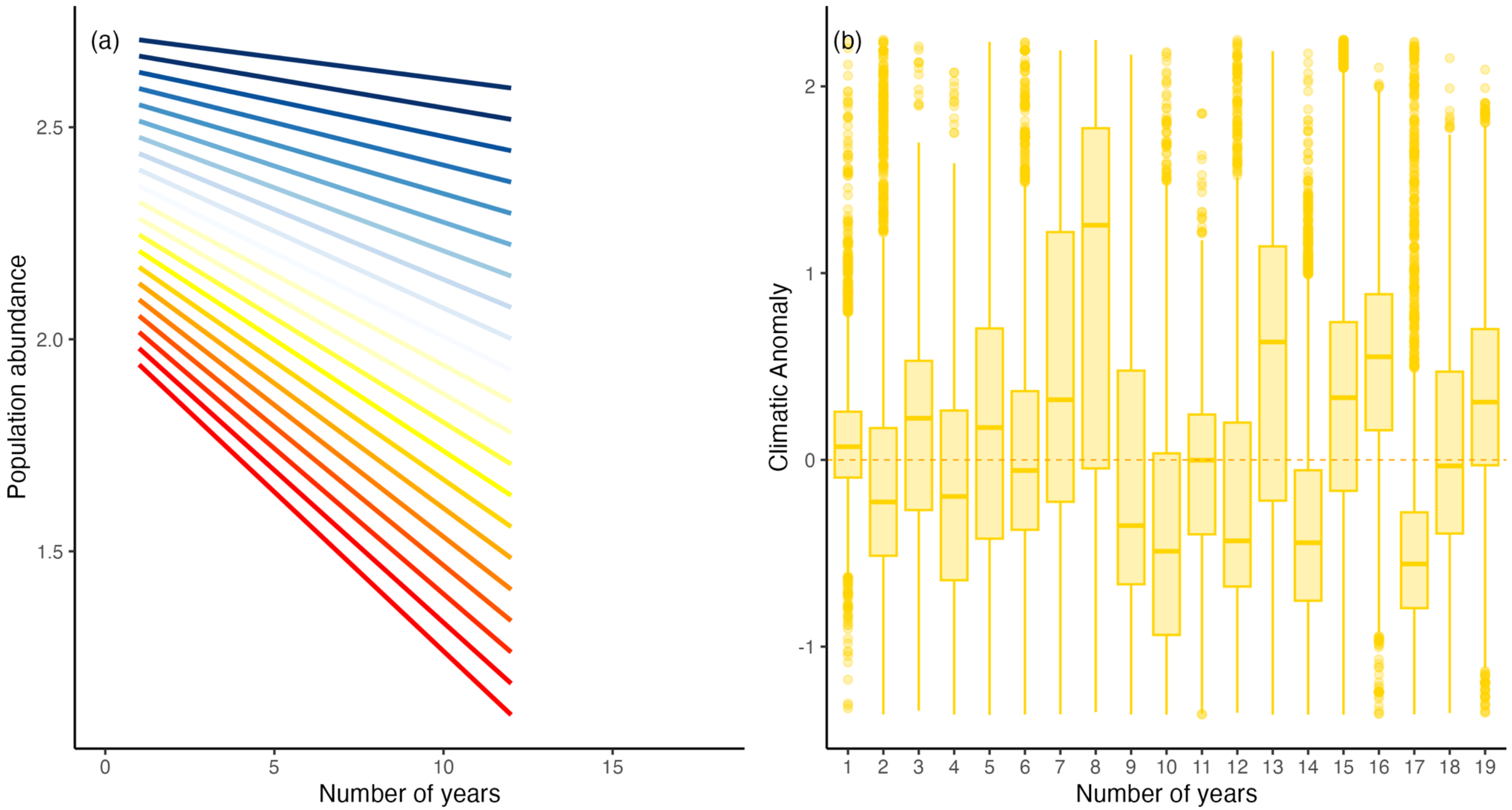

Supplementary Figure 68. Population abundance (log scale) and climatic anomalies over time for *Satyrium acaciae*, a species best adapted to global climatic anomalies in temperature during the pre-flight period of the previous year (t-1) of their adult stage. Panel **a** shows population abundance over time in relation to the population position within the species bioclimatic range (Supplementary Fig. 35). Divergent responses according to the position of the site in the species bioclimatic range are shown at 0.1 intervals from the leading (range position = -1) to the trailing margin (range position = 1), displayed from the leading to trailing (blue and red, respectively; white indicates centre). Panel **b** shows the boxplot of the local climatic anomalies over time for the species. Dashed line related to the lack of climatic anomalies.

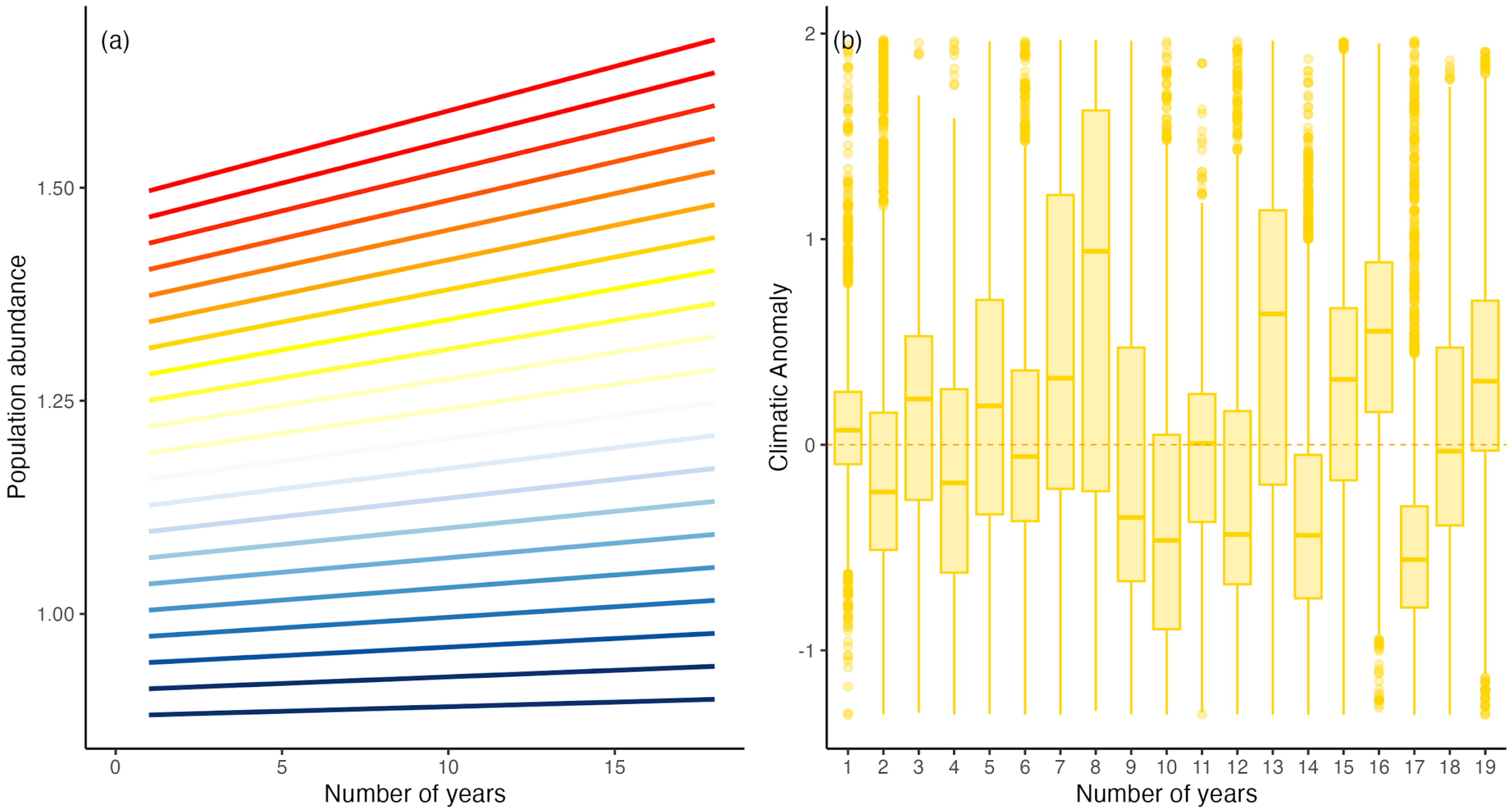

Supplementary Figure 69. Population abundance (log scale) and climatic anomalies over time for *Satyrium pruni*, a species best adapted to global climatic anomalies in temperature during the post-flight period of the previous year (t-1) of their adult stage. Panel **a** shows population abundance over time in relation to the population position within the species bioclimatic range (Supplementary Fig. 35). Divergent responses according to the position of the site in the species bioclimatic range are shown at 0.1 intervals from the leading (range position = -1) to the trailing margin (range position = 1), displayed from the leading to trailing (blue and red, respectively; white indicates centre). Panel **b** shows the boxplot of the local climatic anomalies over time for the species. Dashed line related to the lack of climatic anomalies.

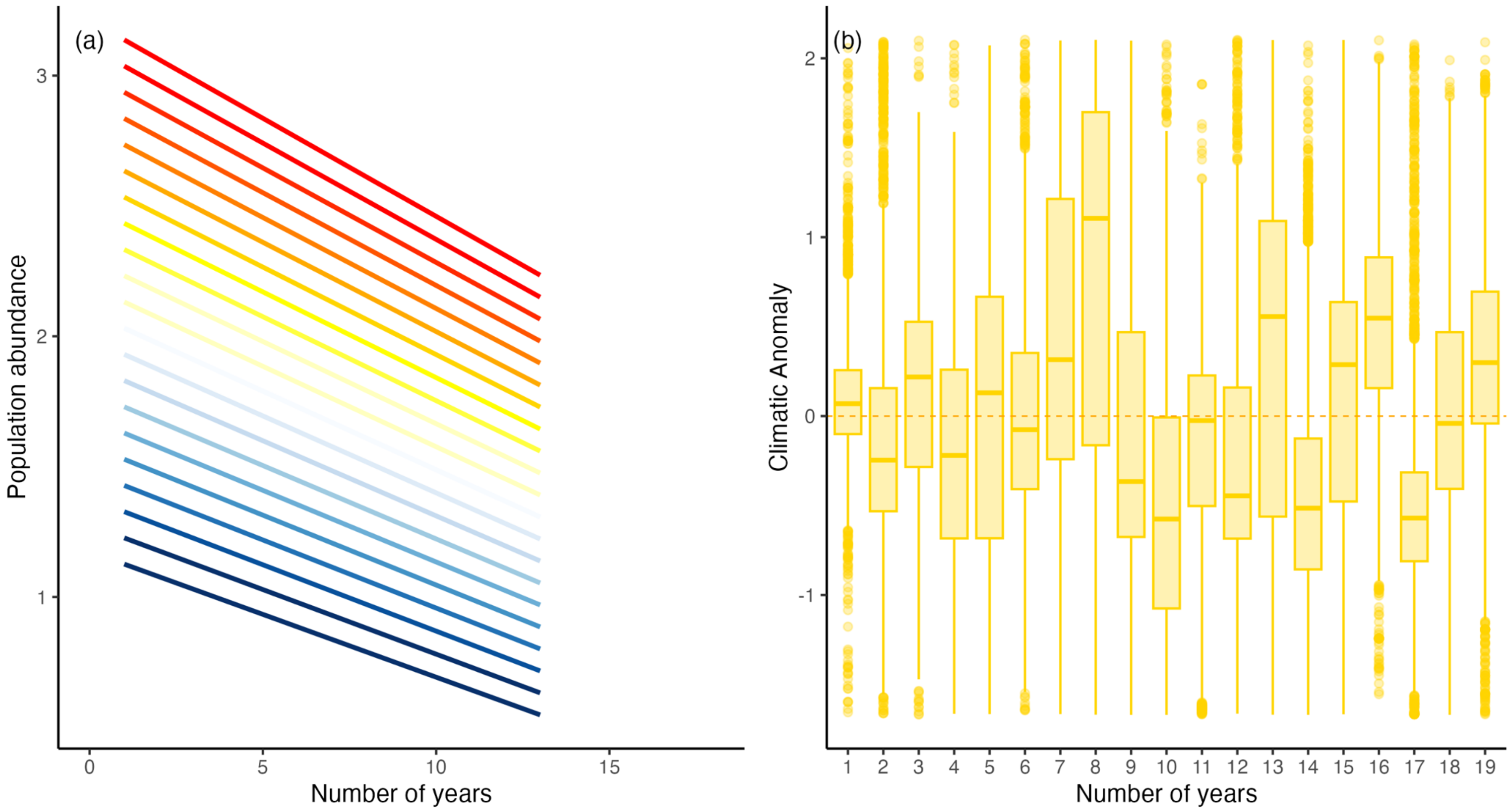

Supplementary Figure 70. Population abundance (log scale) and climatic anomalies over time for *Satyrium spini*, a species best adapted to global climatic anomalies in precipitation during the post-flight period of the previous year (t-1) of their adult stage. Panel **a** shows population abundance over time in relation to the population position within the species bioclimatic range (Supplementary Fig. 35). Divergent responses according to the position of the site in the species bioclimatic range are shown at 0.1 intervals from the leading (range position = -1) to the trailing margin (range position = 1), displayed from the leading to trailing (blue and red, respectively; white indicates centre). Panel **b** shows the boxplot of the local climatic anomalies over time for the species. Dashed line related to the lack of climatic anomalies.

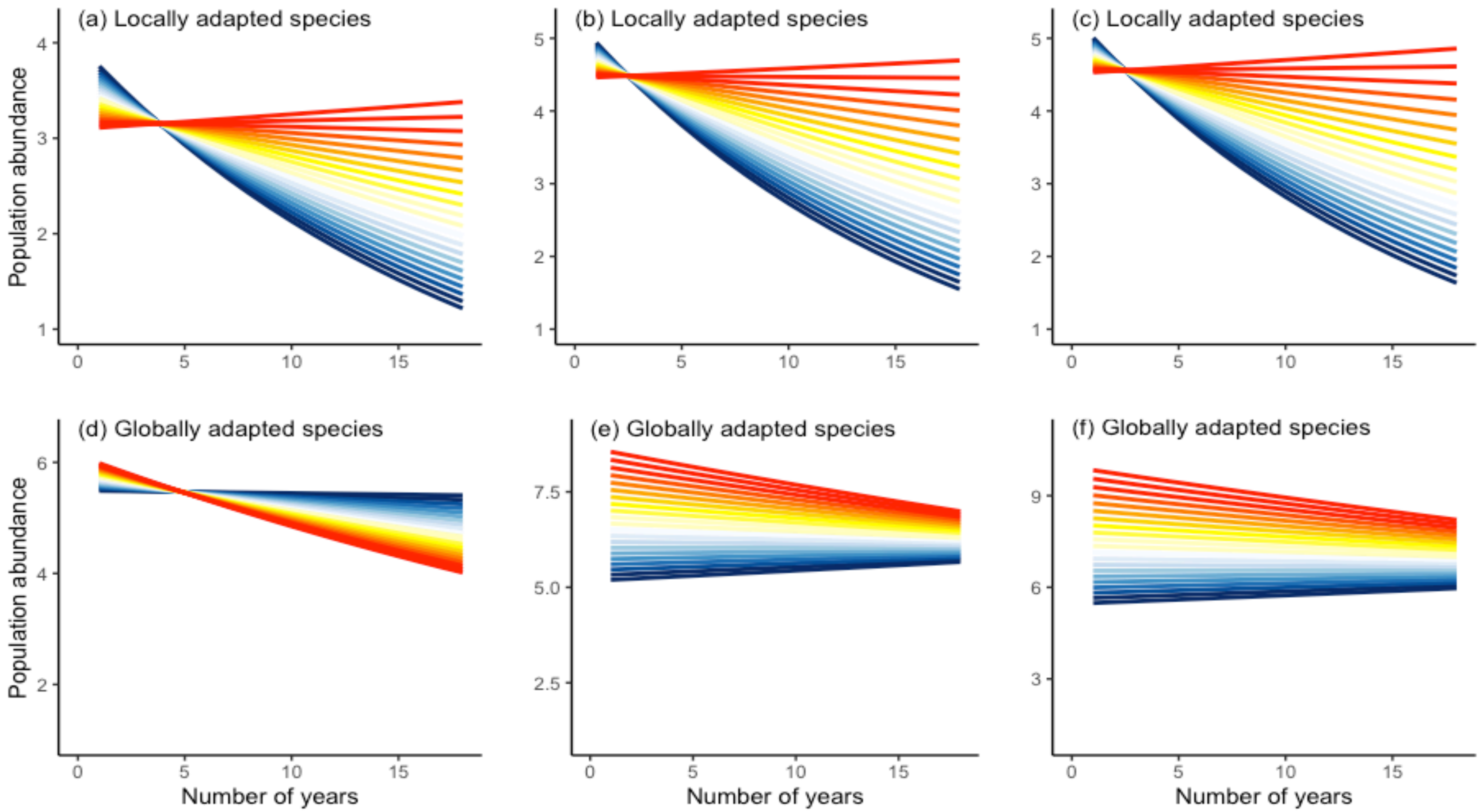

Supplementary Figure 71. Population abundances in relation to the number of years of observations and the population position within the species bioclimatic range. A conservative approach was taken, with the species selection based on two different thresholds of their degree of local adaptation (dla) from less to more restrictive, and the inclusion or exclusion of potential species outliers. Panels **a**, **d** relate to locally and globally adapted species ($N_{species\ local} = 20$, $N_{species\ global} = 11$), respectively, with dla range 0 to | 1 |, but removing potential outliers; **b**, **e** relate to locally and globally adapted species ($N_{species\ local} = 12$, $N_{species\ global} = 6$), respectively, with dla range 0.025 to | 1 |; and **c**, **f** relate to locally and globally adapted species ($N_{species\ local} = 11$, $N_{species\ global} = 4$), respectively, with dla range 0.025 to | 1 |, and removing potential outliers. Potential outliers were identified based on the species dla: *Parnassius apollo* (dla = 0.7) , *Laeosopis roboris* (dla = -0.22) and *Cupido osiris* (dla = -0.23; Supplementary Fig. 72 and Supplementary Table 4). Divergent responses according to the position of the site in the species bioclimatic range are shown at 0.1 intervals from the leading (range position = -1) to the trailing margin (range position = 1), displayed from the leading to training (blue and red, respectively; white indicates centre).

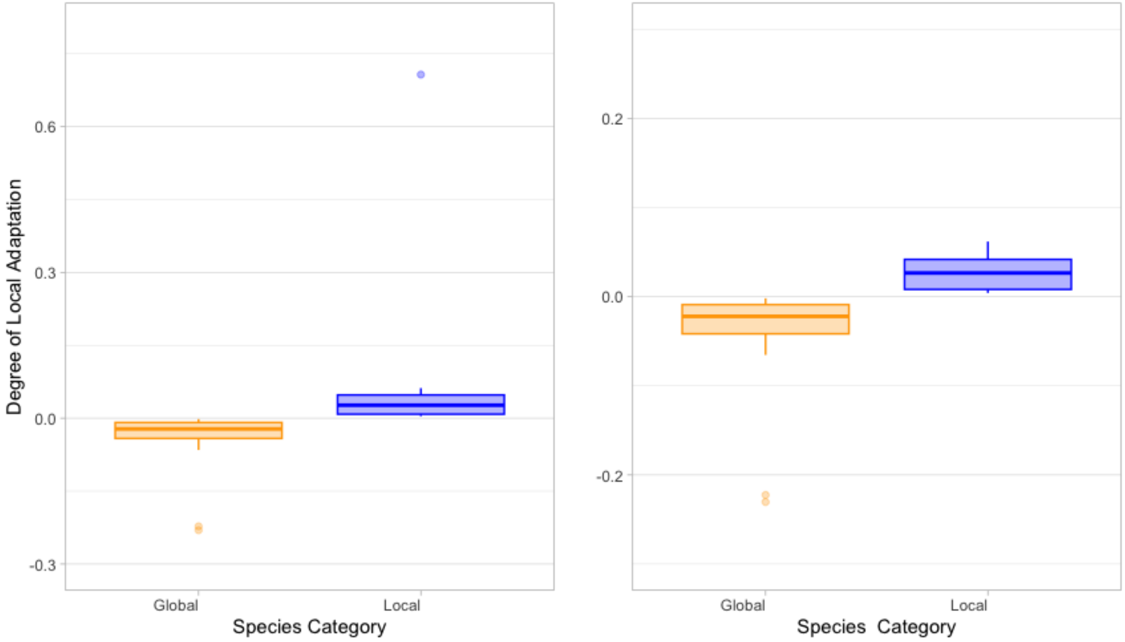

Supplementary Figure 72. Boxplot for the degree of local adaptation (dla) of globally and locally adapted species for (a) all 34 studied species, (b) removing *Parnassius apollo* as an outlier of locally adapted species (dla = 0.7). Two global adapted species could also be interpreted outliers *Laeosopis roboris* and *Cupido osiris* (dla = -0.22 and -0.23). Colours relate to locally (blue) and globally (orange) adapted species.

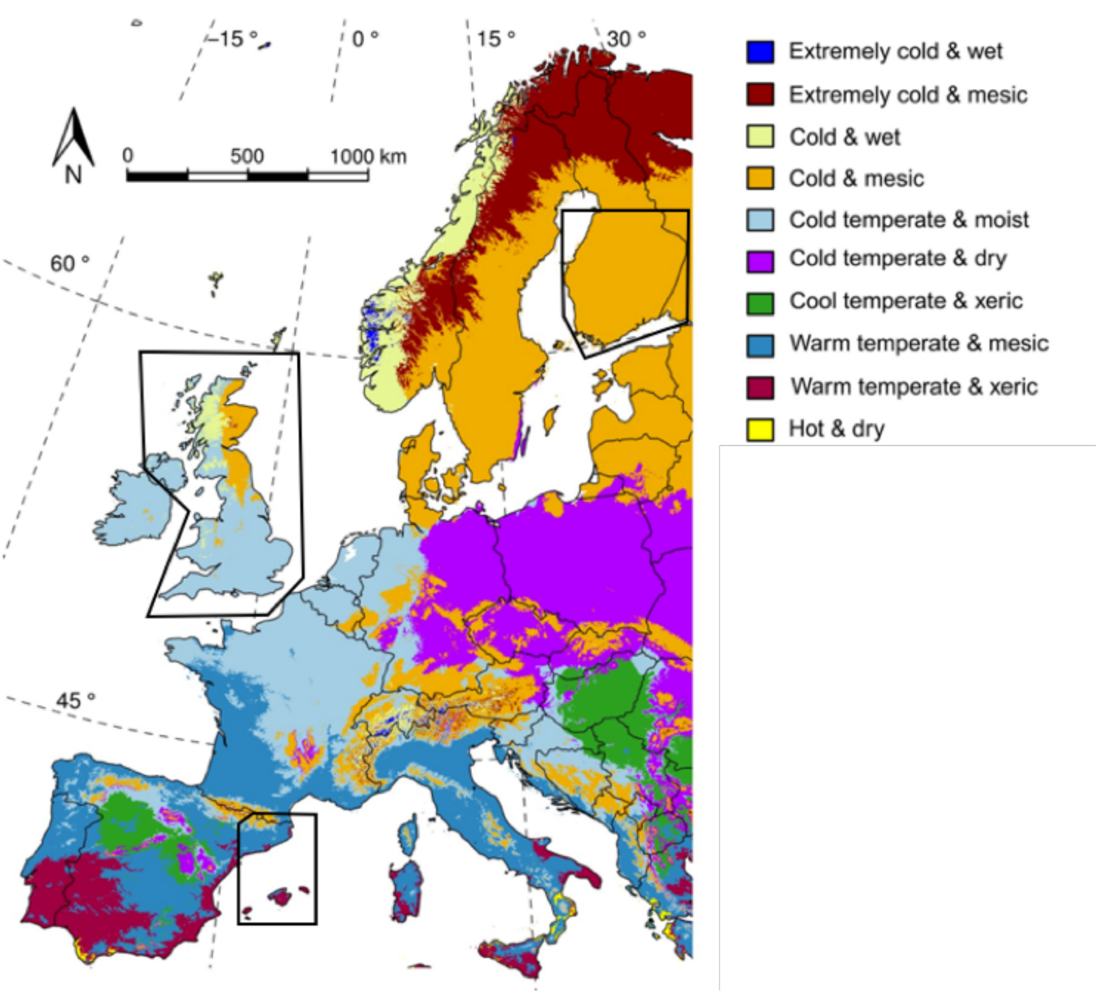

Supplementary Figure 73. Bioclimatic regions across Europe, marking those areas covered by the study. Adapted from Schmuchi[6] and regions based on Metzger[7].

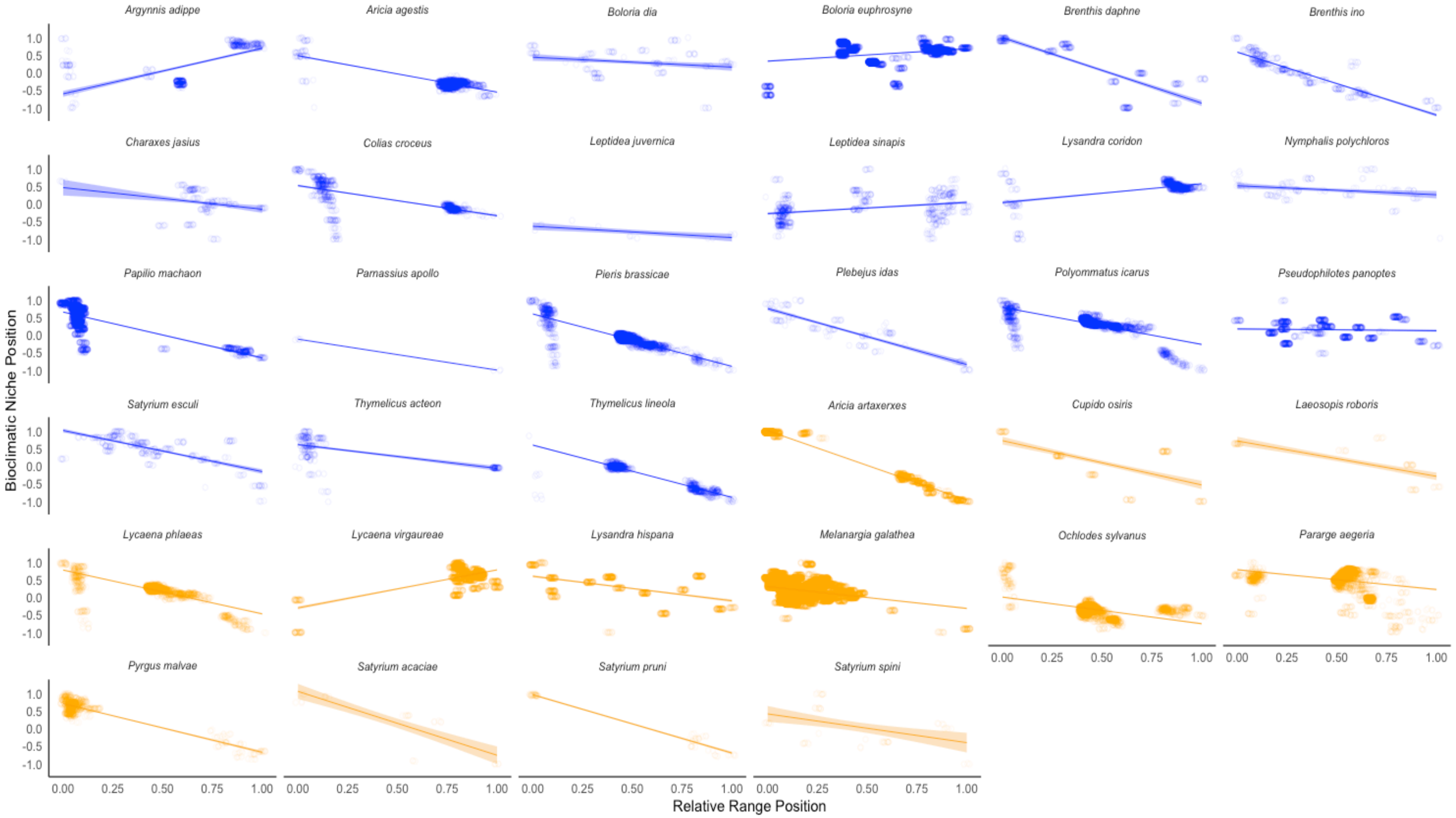

Supplementary Figure 74. Pearson correlation between the population position within the bioclimatic niche and the relative range position of each species. Bioclimatic range position ranges [-1, 1] from leading (position -1) to trailing margins (position 1). Relative range position ranges [0, 1] from lower latitudes (trailing margins) to higher latitudes (leading margins). Negative correlations were expected. All species failing at correlating margins (correlation < 0.4), or with positive correlations ha degree of lower adaptation < | 0.025 |, hence they were not included in the most conservative models (Supplementary Figs. 36 and 71), except for *Arginnis adipe* (dla = 0.033, a locally adapted species to precipitation, present in high altitudinal sites in Spain) and *Lysandra coridon* (dla = 0.066, a locally adapted best adapted to temperature, absent in the UK). All other correlations were negative and significant (p-values < 0.0056). Colours indicate locally adapted species in blue and globally adapted species in orange.

Supplementary Table 1. List of the 34 butterfly species whose populations showed a clear response to the local climatic anomalies of a specific temporal window (phenological period and year (*t*) or *t-1*), depending on the species; Supplementary Figs 1-34). Columns show the species category (locally or globally adapted) in relation to the spatial scale of their responses which is based on the degree of local adaptation being locally adapted species those with dla=]0, 1] and globally adapted those with dla = [-1, 0[. The climatic variable to which each species was most sensitive to, as well as the period and time year are also shown. All variables per species were extracted from Melero et al. 2022[5], except the species distribution which was extracted from the eBMS and the GBIF datasets.

Species	Category	Degree Local Adaptation	Climatic variable	Period	Year	Distribution
Argynnis adippe	Locally	0.03356	Precipitation	preFP	t-1	Most EU, except northern UK and Scandinavia.
Aricia agestis	Locally	0.05069	Temperature	FP	t-1	Most of EU, except northern UK and Scandinavia.
Boloria dia	Locally	0.01483	Precipitation	postFP	t-1	Most of EU, except northern UK and Scandinavia.
Boloria euphrosyne	Locally	0.00799	Precipitation	FP	t	Most of EU.
Brenthis daphne	Locally	0.05864	Precipitation	FP	t	Most of EU, except in the UK.
Brenthis ino	Locally	0.06191	Temperature	OW	t-1	Most of EU, except in the UK.
Charaxes jasius	Locally	0.00765	Precipitation	preFP	t-1	North Africa and south EU.
Colias croceus	Locally	0.02644	Temperature	postFP	t-1	Most of EU.
Leptidea juvernica	Locally	0.01583	Precipitation	FP	t-1	Most of EU.
Leptidea sinapis	Locally	0.00436	Aridity	postFP	t-1	Across EU.
Lysandra coridon	Locally	0.06019	Temperature	postFP	t-1	Most of EU, except Scandinavia and south Italy.
Nymphalis polychloros	Locally	0.00480	Precipitation	preFP	t-1	North Africa and across EU.
Papilio machaon	Locally	0.00416	Temperature	FP	t	North Africa and across EU.
Parnassius apollo	Locally	0.70706	Precipitation	preFP	t-1	Across EU (alpine), not in the UK.
Pieris brassicae	Locally	0.03990	Temperature	postFP	t-1	North Africa and across EU.
Plebejus idas	Locally	0.00832	Temperature	postFP	t-1	Across EU.
Polyommatus icarus	Locally	0.03414	Temperature	OW	t-1	Across EU.

Pseudophilotes panoptes	Locally	0.02159	Aridity	preFP	t	Spain.
Satyrium esculi	Locally	0.04784	Temperature	preFP	t-1	North Africa and south EU.
Thymelicus acteon	Locally	0.02683	Temperature	preFP	t-1	North Africa and across EU, not in Scandinavia.
Thymelicus lineola	Locally	0.03253	Temperature	postFP	t-1	North Africa and across EU.
Aricia artaxerxes	Globally	-0.01424	Temperature	preFP	t	North Africa and across EU.
Cupido osiris	Globally	-0.23039	Aridity	preFP	t	South EU.
Laeosopis roboris	Globally	-0.22257	Temperature	FP	t	Iberian Peninsula and South France.
Lycaena phlaeas	Globally	-0.00886	Temperature	preFP	t-1	North Africa and across EU.
Lycaena virgaureae	Globally	-0.00209	Precipitation	FP	t	Most of EU.
Lysandra hispana	Globally	-0.03164	Aridity	OW	t	NE Spain, S France, NW Italy
Melanargia galathea	Globally	-0.00181	Temperature	preFP	t	North Africa and across EU.
Ochlodes sylvanus	Globally	-0.04163	Temperature	FP	t-1	North Africa and across EU.
Pararge aegeria	Globally	-0.00488	Precipitation	postFP	t-1	North Africa and across EU.
Pyrgus malvae	Globally	-0.02806	Temperature	postFP	t-1	Most of EU.
Satyrium acaciae	Globally	-0.06535	Temperature	preFP	t-1	Southern and central UE. Absent from the UK and Scandinavia.
Satyrium pruni	Globally	-0.01242	Temperature	postFP	t-1	NE Spain, central EU and UK.
Satyrium spini	Globally	-0.02205	Precipitation	postFP	t-1	Southern and central EU, absent from the UK, Scandinavia

Supplementary Table 2. Model parameter estimates from the LMMs for population change in relation to the climatic anomalies of the year and the population position within the species bioclimatic range (both variables standardized), for (a) locally adapted and (b) globally adapted species, selected based on two different thresholds of their degree of local adaptation (dla) from less to more restrictive, and the inclusion or exclusion of potential species outliers (Supplementary Fig. 72). Potential outliers were identified based on the species dla: *Parnassius apollo* (dla = 0.7), *Laeosopis roboris* (dla = -0.22) and *Cupido osiris* (dla = -0.23). Climatic anomaly relates to the variable most affecting the species (temperature, precipitation or aridity[5]; Supplementary Figs. 1-34 and Supplementary Table 1). Bioclimatic range position is set from the leading (range position = -1) to the trailing margin (range position = 1; Supplementary Fig. 35). Values rounded to two decimal places or next significant figure. Significant p-values are marked in bold. Random effects related to site (Est = 0.04, σ^2 = 0.21; and Est = 0.08, σ^2 = 0.28 for locally and globally adapted species) and species (Est = 0.08, σ^2 = 0.28; and Est = 0.16, σ^2 = 0.4).

		All data								Data without potential species outliers							
		dla: 0 to $\lvert 1 \rvert$ ($N_{species\ local}$ = 21, $N_{species\ global}$ = 13)				dla: $\lvert 0.025 \rvert$ to $\lvert 1 \rvert$ ($N_{species\ local}$ = 12, $N_{species\ global}$ = 6)				dla: 0 to $\lvert 1 \rvert$ ($N_{species\ local}$ = 20, $N_{species\ global}$ = 11)				dla: $\lvert 0.025 \rvert$ to $\lvert 1 \rvert$ ($N_{species\ local}$ = 11, $N_{species\ global}$ = 4)			
	Covariate	Est.	SE	t-value	p-value	Est.	SE	t-value	p-value	Est.	SE	t-value	p-value	Est.	SE	t-value	p-value
(a)	Intercept	0.80	0.07	12.27	**<0.0001**	0.86	0.10	8.29	**<0.0001**	0.81	0.07	12.34	**<0.0001**	0.81	0.07	12.34	**<0.0001**
	Log(Nt)	-0.37	0.004	-92.28	**<0.0001**	-0.37	0.01	-71.56	**<0.0001**	-0.37	0.004	-92.28	**<0.0001**	-0.37	0.004	-92.28	**<0.0001**
	Range position	0.08	0.02	4.13	**<0.0001**	0.19	0.03	7.14	**<0.0001**	0.08	0.02	4.12	**<0.0001**	0.08	0.02	4.12	**<0.0001**
	Climatic anomaly	-0.12	0.004	-28.15	**<0.0001**	-0.14	0.005	-30.02	**<0.0001**	-0.11	0.004	-28.84	**<0.0001**	-0.11	0.004	-28.84	**<0.0001**
	Climatic anomaly^2	-0.04	0.002	-18.18	**<0.0001**	-0.03	0.002	-15.40	0.016	-0.04	0.002	-18.32	**<0.0001**	-0.04	0.002	-18.32	**<0.0001**
	Climatic anomaly: Range position	<0.001	0.009	0.94	0.93	-0.03	0.01	-2.40	**<0.0001**	0.001	0.009	0.094	0.92	<0.001	0.009	0.94	0.93
(b)	Intercept	0.60	0.12	4.97	**<0.0001**	0.94	0.22	4.36	**0.006**	0.69	0.11	6.52	**<0.0001**	1.14	0.25	4.53	0.02
	Log(Nt)	-0.35	0.003	-113.72	**<0.0001**	-0.56	0.01	-61.32	**<0.0001**	-0.35	0.004	-113.5	**<0.0001**	-0.56	0.01	-60.41	**<0.0001**
	Range position	0.03	0.01	1.85	0.06	-0.01	0.04	-0.26	0.80	0.04	0.01	2.57	**0.01**	0.12	0.05	2.57	0.01
	Climatic anomaly	0.01	0.004	3.17	**0.002**	0.08	0.01	10.47	**<0.0001**	0.01	0.004	2.82	**<0.0001**	0.07	0.01	9.54	**<0.0001**
	Climatic anomaly^2	0.005	0.003	1.80	0.07	-0.007	0.005	-1.46	0.14	0.008	0.003	2.69	**0.007**	-0.001	0.01	-0.28	0.78
	Climatic anomaly: Range position	-0.07	0.008	-8.70	**<0.0001**	-0.09	0.02	-5.90	**<0.0001**	-0.07	0.01	-8.71	**<0.0001**	-0.11	0.02	6.29	**<0.0001**

Supplementary Table 3. Model parameter estimates from the LMMs for population change in relation to the climatic anomalies of the year and the population position within the species bioclimatic range (both variables standardized), for the locally adapted species (a) *Brenthis ino* (n = 1194; degree of local adaptation = 0.06) and (b) *Satyrium esculi* (n = 878; degree of local adaptation = 0.05), and for the globally adapted species in panel (c) *Satyrium spini* (n = 112; degree of local adaptation = -0.02) and (d) *Ochlodes sylvanus* (n = 12794; degree of local adaptation = -0.04). Climatic anomaly relates to the variable most affecting the species (temperature, precipitation or aridity[5]; Supplementary Figs. 1-34 and Supplementary Table 1). Bioclimatic range position is set from the leading (range position = -1) to the trailing margin (range position = 1; Supplementary Fig. 35). Values rounded to two decimal places or next significant figure. Significant p-values are marked in bold.

	Brenthis ino				*Satyrium esculi*				*Satyrium spini*				*Ochlodes sylvanus*			
	Est.	SE	t-value	p-value	Est.	SE	t-value	p-value	Est.	SE	t-value	p-value	Est.	SE	t-value	p-value
Intercept	1.90	0.11	17.73	**<0.0001**	2.23	0.21	10.49	**<0.0001**	2.08	0.27	7.62	**<0.0001**	1.67	0.05	35.49	**<0.0001**
Log(Nt)	-0.67	0.03	-23.86	**<0.0001**	-0.69	0.03	-21.53	**<0.0001**	-1.14	0.07	-15.97	**<0.0001**	-0.76	0.01	-90.42	**<0.0001**
Range position	0.16	0.17	0.95	0.35	0.46	0.30	1.55	0.13	0.19	0.45	0.42	0.68	0.05	0.10	0.53	0.6
Climatic anomaly	-0.08	0.01	-7.70	**<0.0001**	0.23	0.06	3.53	**<0.0001**	0.29	0.06	4.64	**<0.0001**	0.07	0.01	7.23	**<0.0001**
Climatic anomaly^2	-0.02	0.00	-6.80	**<0.0001**	-0.30	0.05	-5.43	**<0.0001**	-0.03	0.06	-0.60	0.55	-0.01	0.00	-2.36	0.02
Climatic anomaly: Range position	0.01	0.01	0.40	0.69	-0.56	0.11	-5.14	**<0.0001**	0.32	0.11	2.92	0.004	-0.14	0.02	-5.57	0.93

Supplementary Table 4. Model parameter estimates from the phylogenetic LMMs for population change in relation to the climatic anomalies of the year and the population position within the species bioclimatic range (both variables standardized), for (a) locally adapted and (b) globally adapted species, including all 34 species to account for their phylogenies. Climatic anomaly relates to the variable most affecting the species (temperature, precipitation or aridity[5]; Supplementary Figs. 1-34 and Supplementary Table 1). Bioclimatic range position is set from the leading (range position = -1) to the trailing margin (range position = 1; Supplementary Fig. 35). The butterfly phylogenetic tree was extracted from the most recently published phylogenetic tree of European butterflies[8]. Values rounded to two decimal places or next significant figure. Significant p-values are marked in bold. Random effects related to site (Est = 0.04, σ^2 = 0.21; and Est = 0.08, σ^2 = 0.28, for locally and globally adapted species), species (Est = 0.07, σ^2 = 0.27; and Est = 0.04, σ^2 = 0.21), and phylogenetic distances (Est = 0.003, σ^2 = 0.06; and Est = 0.08, σ^2 = 0.28, for locally and globally adapted species respectively).

		dla: 0 to | 1 | ($N_{species\ local}$ = 21, $N_{species\ global}$ = 13)			
	Covariate	Est.	SE	t-value	p-value
(a)	Intercept	0.77	0.08	9.53	**<0.0001**
	Log(Nt)	-0.37	0.004	-92.27	**<0.0001**
	Range position	0.08	0.02	4.13	**<0.0001**
	Climatic anomaly	-0.12	0.004	-28.15	**<0.0001**
	Climatic anomaly^2	-0.035	0.002	-18.18	**<0.0001**
	Climatic anomaly: Range position	<0.001	0.009	0.01	0.94
(b)	Intercept	0.71	0.23	3.12	**0.0018**
	Log(Nt)	-0.35	0.003	-113.89	**<0.0001**
	Range position	0.03	0.01	2.05	**0.03**
	Climatic anomaly	0.01	0.004	2.85	**0.004**
	Climatic anomaly^2	0.005	0.003	1.80	0.07
	Climatic anomaly: Range position	-0.07	0.008	-8.6 0	**<0.0001**

Supplementary Table 5. Model parameter estimates from the LMMs for population trends, in terms of population abundances over time (cumulative number of years of observations) in relation to the population position within the species bioclimatic range (standardized), for (a) locally adapted and (b) globally adapted species, selected based on two different thresholds of their degree of local adaptation (dla) from less to more restrictive, and the inclusion or exclusion of potential species outliers (Supplementary Fig. 72). Potential outliers were identified based on the species dla: *Parnassius apollo* (dla = 0.7), *Laeosopis roboris* (dla = -0.22) and *Cupido osiris* (dla = -0.23). Bioclimatic range position is set from the leading (range position = -1) to the trailing margin (range position = 1; Supplementary Fig. 35). Values rounded to two decimal places or next significant figure. Significant p-values are marked in bold.

		All data								Data without potential species outliers							
		dla: 0 to | 1 | ($N_{species\ local}$ = 21, $N_{species\ global}$ = 13)				dla: | 0.025 | to | 1 | ($N_{species\ local}$ = 12, $N_{species\ global}$ = 6)				dla: 0 to | 1 | ($N_{species\ local}$ = 20, $N_{species\ global}$ = 11)				dla: | 0.025 | to | 1 | ($N_{species\ local}$ = 11, $N_{species\ global}$ = 4)			
	Covariate	Est.	SE	t-value	p-value	Est.	SE	t-value	p-value	Est.	SE	t-value	p-value	Est.	SE	t-value	p-value
(a)	Intercept	1.93	0.21	9.28	**<0.0001**	2.23	0.29	7.61	**<0.0001**	1.95	0.22	9.01	**<0.0001**	2.27	0.31	7.31	**<0.0001**
	Number of years	-0.03	0.01	-3.94	**<0.0001**	-0.03	0.01	-2.82	**0.02**	-0.03	0.01	-3.86	**<0.001**	-0.03	0.01	-2.68	**0.02**
	Range position	-0.11	0.06	-1.99	**<0.05**	-0.07	0.07	-1.06	0.29	-0.12	0.06	-2.02	**0.04**	-0.08	0.07	-1.12	0.26
	Number of years: Range position	0.03	0.004	7.09	**<0.0001**	0.03	0.01	6.36	**<0.0001**	0.03	0.004	7.08	**<0.0001**	0.03	0.01	6.37	**<0.0001**
(b)	Intercept	2.29	0.27	8.53	**<0.001**	2.60	0.51	5.01	**0.004**	2.42	0.29	8.26	<**0.0001**	2.68	0.75	3.57	**0.03**
	Number of years	-0.01	0.01	-1.40	0.19	-0.002	0.003	-0.79	0.51	-0.01	0.01	-1.22	0.25	0.002	0.003	-0.65	0.62
	Range position	0.04	0.05	0.64	0.52	0.20	0.07	2.82	**0.005**	0.05	0.06	0.87	0.38	0.27	0.08	3.56	**<0.0001**
	Number of years: Range position	-0.01	0.00	-2.29	**0.02**	-0.01	0.004	-1.08	0.28	-0.01	0.004	-2.49	**0.01**	-0.01	0.004	-1.45	0.15

Supplementary Table 6. Model selection for population change in relation to the interaction of the standardized covariates based on lowest ΔAIC < 2 for (a) locally adapted (degree of local adaptation, dla =]0, 1]), and (b) globally adapted species (dla = [-1, 0]), selected based on the two different thresholds of their degree of local adaptation from less to more restrictive, and the inclusion or exclusion of potential species outliers. Potential outliers were identified based on the species dla: *Parnassius apollo* (dla = 0.7), *Laeosopis roboris* (dla = -0.22) and *Cupido osiris* (dla = -0.23). The climatic anomaly of the year was set as species specific in relation to the value of the variable most affecting the species (temperature, precipitation or aridity) along the position of the site in the species bioclimatic range. Values rounded to one decimal place. We kept the quadratic term to test for linear relationships as per our hypotheses, but we show all model AIC and ΔAIC. Selected models are marked in bold, models without quadratic term are shown in italic.

		All data				Data without potential sp. outliers			
		dla: 0 to | 1 |		dla: | 0.025 | to | 1 |		dla: 0 to | 1 |		dla: | 0.025 | to | 1 |	
	Model	AIC	ΔAIC	AIC	ΔAIC	AIC	ΔAIC	AIC	ΔAIC
(a)	Log(Nt) + Climatic anomaly + Range position + Climatic anomaly^2	**91224.7**		**59535.9**		**91209.7**		**91209.7**	
	Log(Nt) + Climatic anomaly : Range position + Climatic anomaly^2	91234.2	9.5	**59534.1**	1.8	91219.2	9.5	91219.2	9.5
	Log(Nt) + Climatic anomaly + Range position	*91546.2*	*312*	*59803.6*	*267.7*	*91530.8*	*321.1*	*91530.8*	*321.1*
	Log(Nt) + Climatic anomaly : Range position	*91550.7*	*316.5*	*59788.2*	*252.3*	*91535.5*	*325.8*	*91535.5*	*325.8*
(b)	Log(Nt) + Climatic anomaly : Range position + Climatic anomaly^2	**124730.3**	**8.6**	**21106.5**	**8.7**	**123930.3**	**4.5**	**20343.1**	**10.6**
	Log(Nt) + Climatic anomaly + Range position + Climatic anomaly^2	124794.1	72.4	21126.9	29.1	123994.2	68.4	20370.3	37.8
	Log(Nt) + Climatic anomaly : Range position	*124721.7*		*21097.8*		*123925.8*		*20332.5*	
	Log(Nt) + Climatic anomaly + Range position	*124786.9*	*65.2*	*21124.1*	*26.3*	*123991.7*	*65.9*	*20363.6*	*31.1*

Supplementary Table 7. Model selection for population abundances in relation to the interaction of the standardized covariates based on lowest ΔAIC < 2 for for the locally adapted species (a) *Brenthis ino* (n = 1194; degree of local adaptation = 0.06) and (b) *Satyrium esculi* (n = 878; degree of local adaptation = 0.05), and for the globally adapted species (c) *Satyrium spini* (n = 112; degree of local adaptation = -0.02) and (d) *Ochlodes sylvanus* (n = 12794; degree of local adaptation = -0.04), in response to the climatic anomaly of the year most affecting the species (temperature, precipitation or aridity) along the position of the site in the species bioclimatic range. Values rounded to one decimal place. We kept the quadratic term to test for linear relationships as per our hypotheses, but we show all model AIC and ΔAIC. Selected models are marked in bold, models without quadratic term are shown in italic.

	(a)		(b)		(c)		(d)	
Model	AIC	ΔAIC	AIC	ΔAIC	AIC	ΔAIC	AIC	ΔAIC
Log(Nt) + Climatic anomaly + Range position + Climatic anomaly^2	**2321.9**		2395.5	21.5	257.4	8.9	26946.5	23.5
Log(Nt) + Climatic anomaly : Range position + Climatic anomaly^2	2330.4	8.5	**2374.0**		**253.9**	5.4	**26923.0**	
Log(Nt) + Climatic anomaly + Range position	*2355.4*	*33.5*	*2412.8*	*38.8*	*251.8*	*8.9*	*26941.2*	*18.2*
Log(Nt) + Climatic anomaly : Range position	*2363.9*	*42*	*2397.0*	*23*	*248.5*		*26917.4*	*5.6*

References

1. Hoffmann, A. A., Chown, S. L. & Clusella-Trullas, S. Upper thermal limits in terrestrial ectotherms: how constrained are they? *Functional Ecology* **27**, 934–949 (2013).
2. Huey, R. B. & Kingsolver, J. G. Evolution of thermal sensitivity of ectotherm performance. *Trends in Ecology & Evolution* **4**, 131–135 (1989).
3. Hutchison, V. H. & Maness, J. D. The Role of Behavior in Temperature Acclimation and Tolerance in Ectotherms. *American Zoologist* **19**, 367–384 (1979).
4. Briere, J. F., Pracros, P., Le Roux, A. Y. & Pierre, J. S. A novel rate model of temperature-dependent development for arthropods. *Environmental Entomology* **28**, 22–29 (1999).
5. Melero, Y. *et al.* Local adaptation to climate anomalies relates to species phylogeny. *Commun Biol* **5**, 1–9 (2022).
6. Schmucki, R. *et al.* A regionally informed abundance index for supporting integrative analyses across butterfly monitoring schemes. *Journal of Applied Ecology* **53**, 501–510 (2016).
7. Metzger, M. J. *et al.* A high-resolution bioclimate map of the world: a unifying framework for global biodiversity research and monitoring. *Global Ecology and Biogeography* **22**, 630–638 (2013).
8. Dapporto, L. *et al.* Integrating three comprehensive data sets shows that mitochondrial DNA variation is linked to species traits and paleogeographic events in European butterflies. *Molecular Ecology Resources* **19**, 1623–1636 (2019).
