# Supplementary material for: Species responses to weather anomalies depend on local adaptation and range position
Source: Commun Biol. 2025 Apr 24;8:660. doi: 10.1038/s42003-025-08032-9 (PMC12022152; doi:10.1038/s42003-025-08032-9)
Supplement: Supplementary file 2 — Reporting Summary [file 42003_2025_8032_MOESM2_ESM.pdf]

nature portfolio

Corresponding author(s): Yolanda Melero

Last updated by author(s): Mar 12, 2025

# Reporting Summary

Nature Portfolio wishes to improve the reproducibility of the work that we publish. This form provides structure for consistency and transparency in reporting. For further information on Nature Portfolio policies, see our Editorial Policies and the Editorial Policy Checklist.

## Statistics

For all statistical analyses, confirm that the following items are present in the figure legend, table legend, main text, or Methods section.

| n/a | Confirmed | |
|---|---|---|
| ☐ | ☒ | The exact sample size (*n*) for each experimental group/condition, given as a discrete number and unit of measurement |
| ☐ | ☒ | A statement on whether measurements were taken from distinct samples or whether the same sample was measured repeatedly |
| ☐ | ☒ | The statistical test(s) used AND whether they are one- or two-sided<br>*Only common tests should be described solely by name; describe more complex techniques in the Methods section.* |
| ☐ | ☒ | A description of all covariates tested |
| ☐ | ☒ | A description of any assumptions or corrections, such as tests of normality and adjustment for multiple comparisons |
| ☐ | ☒ | A full description of the statistical parameters including central tendency (e.g. means) or other basic estimates (e.g. regression coefficient) AND variation (e.g. standard deviation) or associated estimates of uncertainty (e.g. confidence intervals) |
| ☐ | ☒ | For null hypothesis testing, the test statistic (e.g. *F*, *t*, *r*) with confidence intervals, effect sizes, degrees of freedom and *P* value noted<br>*Give P values as exact values whenever suitable.* |
| ☒ | ☐ | For Bayesian analysis, information on the choice of priors and Markov chain Monte Carlo settings |
| ☒ | ☐ | For hierarchical and complex designs, identification of the appropriate level for tests and full reporting of outcomes |
| ☐ | ☒ | Estimates of effect sizes (e.g. Cohen's *d*, Pearson's *r*), indicating how they were calculated |

*Our web collection on statistics for biologists contains articles on many of the points above.*

## Software and code

Policy information about availability of computer code

| | |
|---|---|
| Data collection | Data was provided by the European Butterfly Monitor Scheme coordinated by the by the Centre for Ecology & Hydrology (.txt and . RSD formats). Climatic data was collected via ECAD. Species degree of local adaptation was extracted from Melero et al 2022 https://doi.org/10.1038/s42003-022-03088-3, and it is provided in the SM. Species bioclimatic range was calculated based on the count and climactic data, specified in Methods. |
| Data analysis | All analyses were conducted in R 4.3.2 (R Core Team, 2019), using the following packages: lme4 to fit our models and package MuMin to estimate the marginal and conditional variances. Bioclimatic range construction was also set in R via a formula explained in methods, but its R code can be added to Github if preferred by the editorial board. |

For manuscripts utilizing custom algorithms or software that are central to the research but not yet described in published literature, software must be made available to editors and reviewers. We strongly encourage code deposition in a community repository (e.g. GitHub). See the Nature Portfolio guidelines for submitting code & software for further information.

## Data

Policy information about availability of data

All manuscripts must include a data availability statement. This statement should provide the following information, where applicable:

- Accession codes, unique identifiers, or web links for publicly available datasets
- A description of any restrictions on data availability
- For clinical datasets or third party data, please ensure that the statement adheres to our policy

The data required to perform the analyses is available at Zenodo 10.5281/zenodo.15012265, with the codes available at GitHub and Zenodo 10.5281/zenodo.15065537. The raw count butterfly data that support the findings of this study is available from the European Butterfly Monitor Scheme via a signed license agreement (https://butterfly-monitoring.net/). Climatic data are available via ECAD website (https://www.ecad.eu/).

## Research involving human participants, their data, or biological material

Policy information about studies with human participants or human data. See also policy information about sex, gender (identity/presentation), and sexual orientation and race, ethnicity and racism.

| | |
|---|---|
| Reporting on sex and gender | NA |
| Reporting on race, ethnicity, or other socially relevant groupings | NA |
| Population characteristics | NA |
| Recruitment | NA |
| Ethics oversight | NA |

Note that full information on the approval of the study protocol must also be provided in the manuscript.

# Field-specific reporting

Please select the one below that is the best fit for your research. If you are not sure, read the appropriate sections before making your selection.

☐ Life sciences ☐ Behavioural & social sciences ☒ Ecological, evolutionary & environmental sciences

For a reference copy of the document with all sections, see nature.com/documents/nr-reporting-summary-flat.pdf

# Ecological, evolutionary & environmental sciences study design

All studies must disclose on these points even when the disclosure is negative.

| | |
|---|---|
| Study description | This study was designed to assess the role of local adaptation in interaction with the position of the population along the species bioclimatic range on species population responses to climatic anomalies. We found populations of locally adapted species (previously defined as the relative sensitivity to weather anomalies occurring at the population site) to respond to climatic anomalies negatively and similarly across the species range. However, we expected globally adapted species (i.e., species for which optimal conditions are those occurring at the center of their distribution) to respond positively or negatively to climate anomalies depending on the location in the species range. Consequently, we also detected variation in population trends over time for locally and globally adapted species, related to whether the population was at the trailing, center, or margin of the range. This is a real-world field experiment with long-term data at a large spatial scale, which challenges the generally accepted pattern of climatic change leading to increased population abundance at the leading margins while decreasing the abundance of populations at the trailing margins. This finding has implications for forecasting species population and distribution trends and for informing appropriate conservation actions. Despite the novelty of these findings, our results are aligned with expectations from theory and previous laboratory studies around thermal performance ecology. |
| Research sample | We used butterfly count data of 34 species observed across the 813 monitoring sites from UK, Finland and Spain, covering six European bioclimatic regions, from 1999 until 2017. A total of 97,664 site-year-species data points. The butterfly count data used are available via the European Butterfly Monitor Schemes coordinated by the Centre for Ecology & Hydrology. There is no animal manipulation. |
| Sampling strategy | The Schemes consist on fixed transects along which a network of volunteers perform visual identification and counts of butterfly species. Monitoring is done weekly during the butterfly flight season, which varies depending on the climatic zone within the range of March to end of September. We used data collected from 1999 until 2017. |
| Data collection | Data is collected by each volunteer at their fixed assigned transects following a standard methodology. Volunteers upload the data to |

| | |
|---|---|
| Data collection | their national butterfly monitoring schemes (e.g., UKBMS). The scheme coordinators review it and send it to the Centre for Ecology & Hydrology (the European coordinator). |
| Timing and spatial scale | Monitoring is done weekly by volunteers in their assigned transects, following the established Pollard Walk for butterfly monitoring. Counts are conducted during the butterfly flight season, which varies depending on the climatic zone within the range of March to end of September. Transects are typically about 2-4km long and are divided into sections corresponding to different habitat or management units. We used data from 1999 (the start of the Finish BMS) to 2017 across UK, Finland and Spain to cover six European bioclimatic regions. We have a total of 813 monitoring sites for 34 species (97,664 site-year-species data points). |
| Data exclusions | NA |
| Reproducibility | All butterfly monitor schemes follow the same methodology, counts are done via Pollard Walk methodology and are performed during the flight period of the adults along a series of fixed transects of 5m width, 5m height and ca. 2-4km of length taking between 45 minutes and two hours to walk. Transect walks are undertaken between 10.45am and 3.45pm and only when weather conditions are suitable for butterfly activity; hence, not done when the temperature is below 13°C (in northern upland areas this may be reduced to 11°C) or above 35°C , when there are less than 40% of sun, or when wind speeds are above 5 on the Beaufort scale. |
| Randomization | No randomization was required in this study. |
| Blinding | No blinding was performed in this study. |

Did the study involve field work? ☒ Yes ☐ No

## Field work, collection and transport

| | |
|---|---|
| Field conditions | Habitats, and climatic variables vary along the sites. |
| Location | Series of different locations along UK (701 sites), 59 (Spain) and Finland (53). Gathering from latitudes of ca. 65ª 30' N to 40ª 60' N and longitudes from -5º 50' W to 31º 52' W. |
| Access & import/export | Access to the field sites was done by volunteers. Transects are always accessible via foot, sometimes upon arrival by transport. All field sites are accessed in full compliance of local legislation. In case of need of permits, these are obtained each year for the whole sampling season, issued by the correspondent administration. |
| Disturbance | The field observations did not induce any relevant disturbance. |

# Reporting for specific materials, systems and methods

We require information from authors about some types of materials, experimental systems and methods used in many studies. Here, indicate whether each material, system or method listed is relevant to your study. If you are not sure if a list item applies to your research, read the appropriate section before selecting a response.

### Materials & experimental systems

| n/a | Involved in the study | |
|---|---|---|
| ☒ | ☐ | Antibodies |
| ☒ | ☐ | Eukaryotic cell lines |
| ☒ | ☐ | Palaeontology and archaeology |
| ☐ | ☒ | Animals and other organisms |
| ☒ | ☐ | Clinical data |
| ☒ | ☐ | Dual use research of concern |
| ☒ | ☐ | Plants |

### Methods

| n/a | Involved in the study | |
|---|---|---|
| ☒ | ☐ | ChIP-seq |
| ☒ | ☐ | Flow cytometry |
| ☒ | ☐ | MRI-based neuroimaging |

## Animals and other research organisms

Policy information about studies involving animals; ARRIVE guidelines recommended for reporting animal research, and Sex and Gender in Research

| | |
|---|---|
| Laboratory animals | No laboratory animals were used in this study |
| Wild animals | Butterfly individuals were counted using visual identification. No manipulation was done. |
| Reporting on sex | Sex was not considered in this study, as per many butterfly species differentiation requires laboratory work. Besides, it was out of the scope of the study. |

| | |
|---|---|
| Field-collected samples | No samples were collected in this study |
| Ethics oversight | This study has no ethical issues. |

Note that full information on the approval of the study protocol must also be provided in the manuscript.

## Plants

| | |
|---|---|
| Seed stocks | NA |
| Novel plant genotypes | NA |
| Authentication | NA |
